# Approach to the Core Structure of Signermycin B

**DOI:** 10.1002/open.202400103

**Published:** 2024-05-29

**Authors:** Khoa Linh Pham, Martin E. Maier

**Affiliations:** ^1^ Eberhard Karls Universität Tübingen Institut für Organische Chemie Auf der Morgenstelle 18 72076 Tübingen Germany

**Keywords:** signermycin B, decalin, oxy-Cope rearrangement, antibiotic, tetramic acid

## Abstract

Among the natural tetramic acids with a decalinoyl part, signermycin B is unique because it contains a *cis*‐decalin. In this paper, we demonstrate that the *cis*‐decalin section of signermycin B can be accessed by an anionic oxy‐Cope rearrangement. The substrate, a tricyclic dienol was prepared by an intramolecular Diels‐Alder reaction of a masked *ortho*‐benzoquinone, generated by oxidation of an α‐methoxyphenol in presence of *cis*‐2‐hexenol. After a superfluous bromine on the cycloadduct was removed, reaction of the tricyclic ketone with isopropenylmagnesium bromide led to the tricyclic trienol that underwent the oxy‐Cope rearrangement to a *cis*‐decalinone. While we could show, that introduction of the 4‐ethyl substituent (signermycin B numbering) is possible by enolate alkylation, the 4‐*epi*‐isomer was formed.

## Graphical abstract

A tactical sequence of an intramolecular Diels‐Alder reaction of a masked *ortho*‐benzoquine, tethered to *cis*‐2‐hexenol, led to a tricyclic ketone which was reacted with isopropenylmagnesium bromide. This led to the substrate for an anionic oxy‐Cope rearrangement, setting the *cis*‐decalin stereochemistry, required for the core structure of signermycin B.

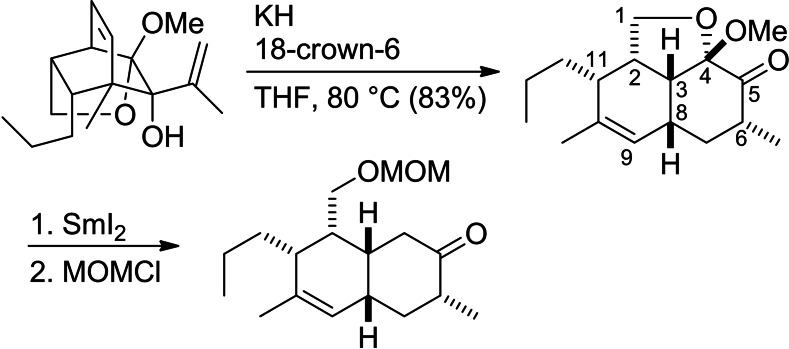

## Introduction

**Figure 1:**
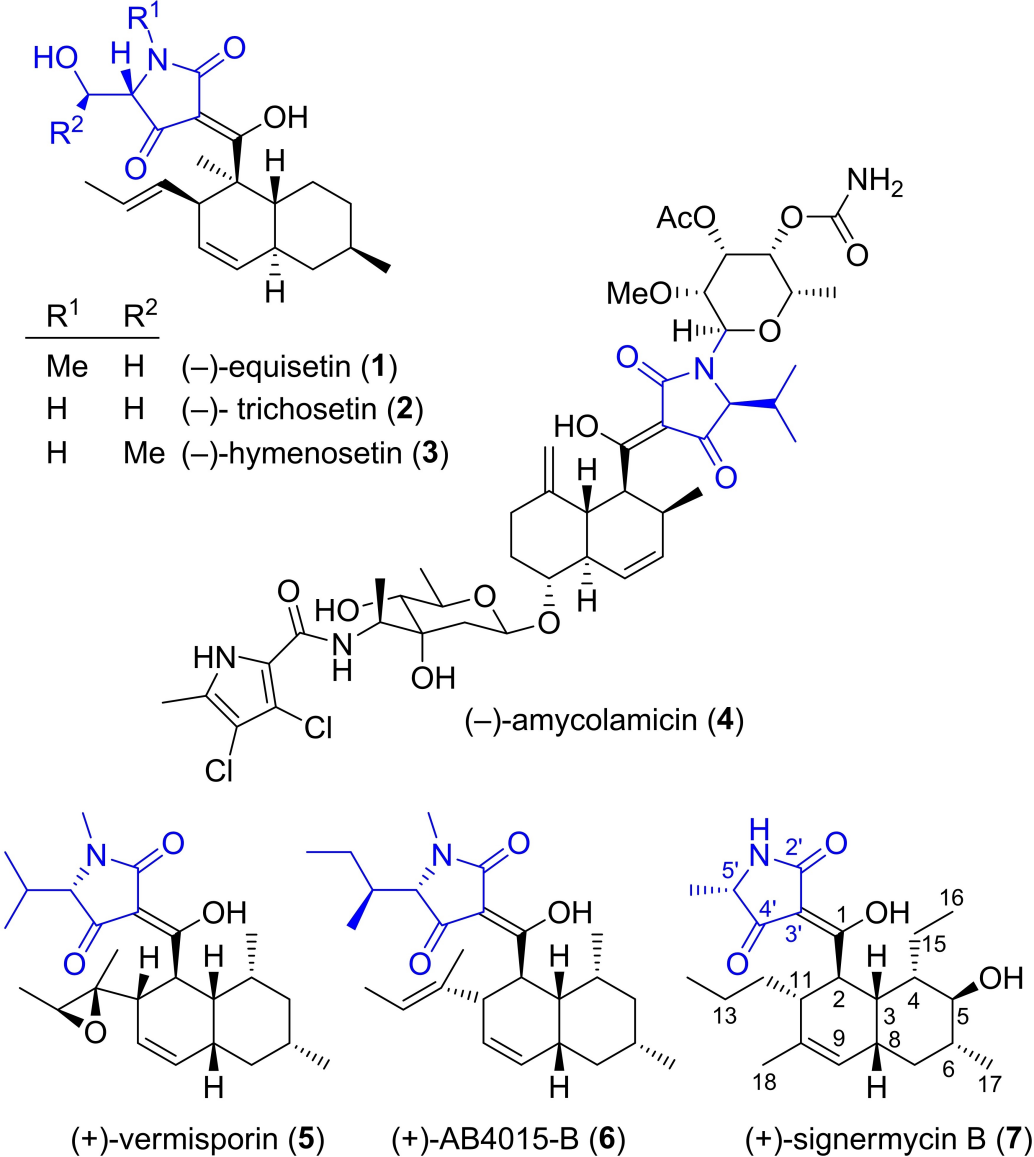
Structures of representative tetramic acids with decalinoyl substituents. The tetramic acid parts are hightlighted in blue.

**Scheme 1:**
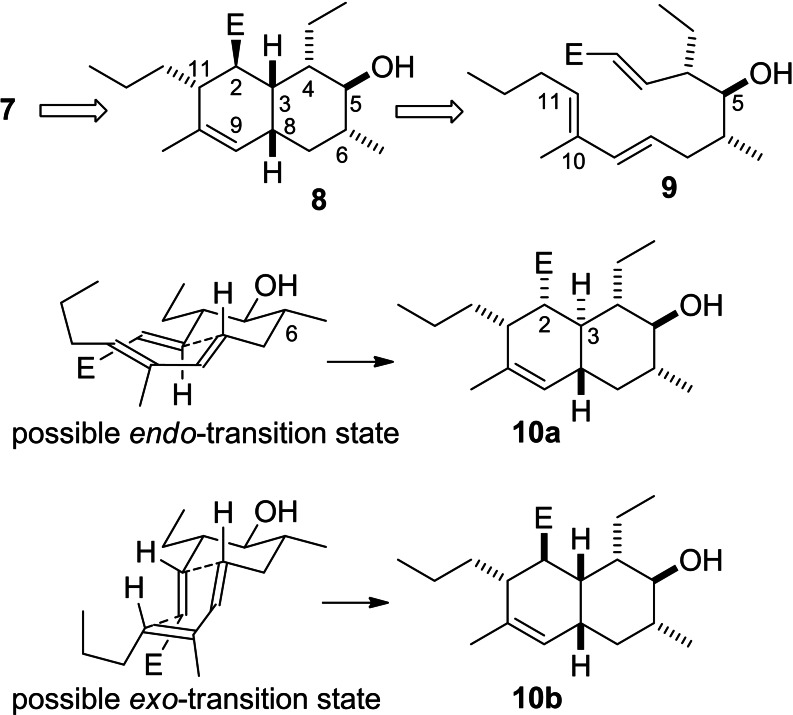
An intramolecular Diels‐Alder strategy towards the core structure of signermycin B (**7**) using trienoate **9** as a substrate would require an *exo*‐transition state in order to provide the *cis*‐decalin system.

Tetramic acids that are acylated at C3 are quite common in nature.^[1]^ They are produced by all kinds of microorganisms and their reported activities include antibiotic, anticancer and anti‐inflammatory effects. The acyl group at C3 can be a long chain polyketide like in aflastatin A,[^2^, ^3^] a chain that forms a macrocycle by binding to C5 or a decalinoyl residue. The decalin part is formed from a triene precursor by a Diels‐Alderase enzyme.^[4]^ This leads to octahydronaphthalene‐1‐carboxylic acid derivatives. In many cases the two six‐membered rings are *trans*‐fused as in (−)‐equisetin[^5^, ^6^] (**1**), (−)‐trichosetin^[7]^ (**2**), (−)‐hymenosetin[^8^, ^9^] (**3**), or amycolamicin[^10^, ^11^] (**4**) (Figure 1). Tetramic acids that feature a *cis*‐decalin system are less common. Examples of this type include (+)‐vermisporin^[12]^ (**5**), (+)‐AB4015‐B^[13]^ (**6**), and (+)‐signermycin B^[14]^ (**7**). These natural products with a *cis*‐decalin show good activities against gram‐positive bacteria. It seems that they act by chelating metal ions and by mimicking phosphate groups in substrates for kinases and phosphatases.^[1b]^

(+)‐Signermycin B (**7**), the focus of this study was described by the group of Utsumi in 2012.^[12]^ It was discovered by screening of *Streptomyces* extracts, employing an assay that detects agents that interfere with the homodimerization domain of the histidine kinase WalK. This kinase is part of a bacterial two‐component system (TCS) that regulates cell wall metabolism and growth.^[15]^ An extracellular signal causes autophosphorylation of WalK at a histidine residue and dimerization. Thereafter, a phosphate group is transferred to WalR, which is the cytosolic response regulator that will bind to a promoter. Signermycin showed antimicrobial activity against Gram‐positive bacteria with MIC values between 3.1 and 6.3 μg mL^–1^. Due to the interesting mode of action and the structural features, we embarked on a synthesis of signermycin B. As compared to other related decalin substituted tetramic acids, the hydroxyl group at C5 (signermycin numbering) is unique. Quite common are alkyl substituents at C4, C6 and C11. Occasionally, a methyl group can be found at C10 on the double bond. It has been shown that the octahydrodecalins in these natural products are formed via Diels‐Alderase catalyzed intramolecular cycloaddition reactions of acyclic triene precursors. In the case of the tetramic acid varicidin A and B, which are fungal natural products, a Diels‐Alderase was found, that catalyzes formation of the *cis*‐decalin ring.[^16^, ^17^] In the case of an *E*,*E*‐diene and an *E*‐configured dienophile thermal or Lewis acid catalyzed intramolecular Diels‐Alder reactions favor the *trans*‐annulated decalin system via *endo*‐equatorial transition states (see Scheme 1). The group of Lei recently showed that tetramic acids featuring a *cis*‐decalin system can be obtained if a keto group is at C4 and the dienophile has *Z*‐configuration.^[18]^

## Results and Discussion

Initially we had considered an approach to the signermycin B decalin part via an intramolecular Diels‐Alder reaction.^[19]^ However, consideration of possible transition states led to the conclusion that a *trans* annulated decalin derivative **10 a** via an *endo*‐transition state would be favored. In the required *exo*‐transition state, the *endo*‐stabilization would be lacking and the dienophile would need to adopt a pseudoaxial orientation (Scheme 1).

**Scheme 2:**
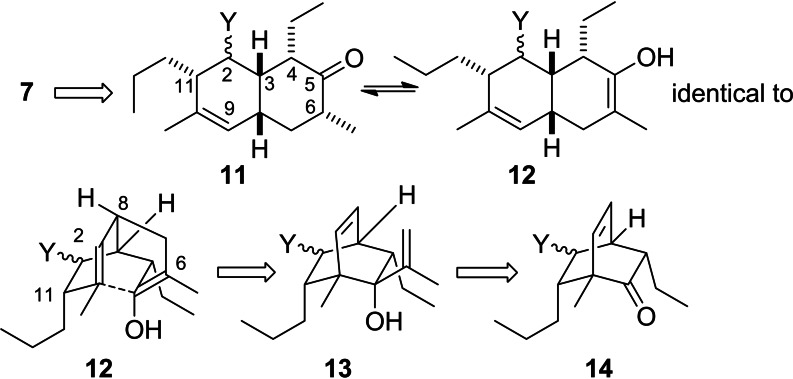
Plan for the synthesis of the decalin part of signermycin B via an oxy‐Cope rearrangement on a bicyclic substrate **13**. Here signermycin B numbering was used.

Therefore, we opted for an alternative strategy that relies on the use of a bicyclic intermediate. Here, an anionic oxy‐Cope rearrangement^[20]^ would be used to secure the desired *cis*‐fusion of the two six‐membered rings (Scheme 2). The substrate for the rearrangement would be obtained by addition of a vinylmetal species to a bicyclo[2.2.2]octenone of type **14**. This concept is known in the literature and has been applied to the synthesis of various natural products.[^21^, ^22^] Typically, the bicyclic substrates for the oxy‐Cope rearrangement are prepared via cycloaddition reactions on masked *ortho*‐benzoquinones. In recent years, the group of Liao has made significant contributions to this strategy.^[23]^ Initially, it might not be necessary to have all the substituents in the B ring in place, since it might be possible to introduce them by alkylation reactions on a C5 ketone.

**Scheme 3:**
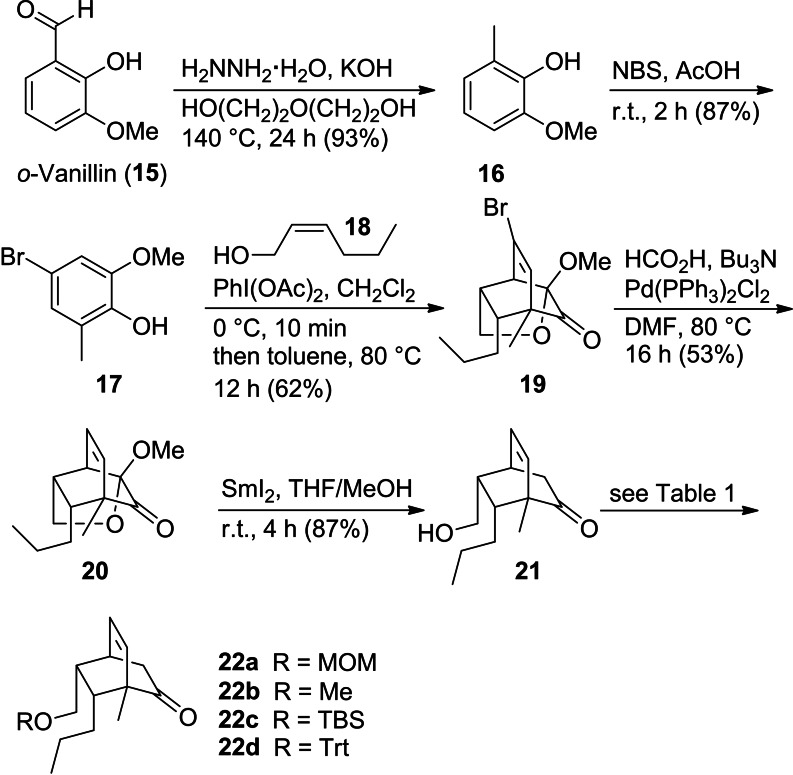
Synthesis of bromophenol **17**, its oxidative conversion to the masked *ortho*‐benzoquinone, followed by intramolecular Diels‐Alder reaction to tricyclic ketone **19**.

The execution of this plan started with the Wolff‐Kishner reduction of *ortho*‐vanillin (**15**) yielding phenol **16** in good yield.^[24]^ This was followed by bromination of the *para*‐position to give 4‐bromophenol derivative **17**.^[25]^ As has been pointed out by Liao et al., the bromination prevents unwanted side reactions during and after the oxidation of the phenol with diacetoxyiodobenzene.^[26]^ Without the bromine, the *para* position of the phenol might be acetylated and on the masked *ortho*‐benzoquinone, the bromine suppresses unwanted dimerization reactions. Upon stirring of a mixture of methoxyphenol **17** and *cis*‐2‐hexenol (**18**) in presence of diacetoxyiodobenzene (DAIB, 1.2 equiv), the intermediate masked *ortho*‐benzoquinone formed. After solvent change to toluene, heating of the mixture to 80 °C induced an intramolecular Diels‐Alder reaction to give polycycle **19** in reasonable yield (Scheme 3). Next, the bromine was removed by palladium‐catalyzed reduction in presence of formic acid leading to tricyclic ketone **20**.[^23^, ^27^] A common method for removal of alkoxy groups in α‐position to a ketone relies on samarium(II) iodide as reducing agent.^[28]^ Applying this reagent to ketone **20** with the mixed acetal in the α‐position provided bicyclic hydroxymethylketone **21**.^[21c]^ Since the subsequent addition of Grignard reagents and the ensuing anionic oxy‐Cope rearrangement might depend to some degree on the nature of the protecting group for the hydroxyl function, we prepared a series of derivatives **22 a**–**22 d** (Table 1).

**Table 1:** Protection of the primary hydroxy group of bicyclic ketone **21**.

entry	conditions	R	yield [%]	compound number
1	MOMCl, *i*Pr_2_NEt, TBAI, CH_2_Cl_2_, r.t., 4 h	MOM	60	**22 a**
2	NaH, MeI, THF, 0–r.t., 2 h	Me	63	**22 b**
3	TBSCl, imidazole, DMF, r.t., 4 h	TBS	81	**22 c**
4	TrtCl, Et_3_N, CH_2_Cl_2_, r.t., 24 h	Trt^[a]^	84	**22 d**

[a] Trt=Trityl.

**Scheme 4:**
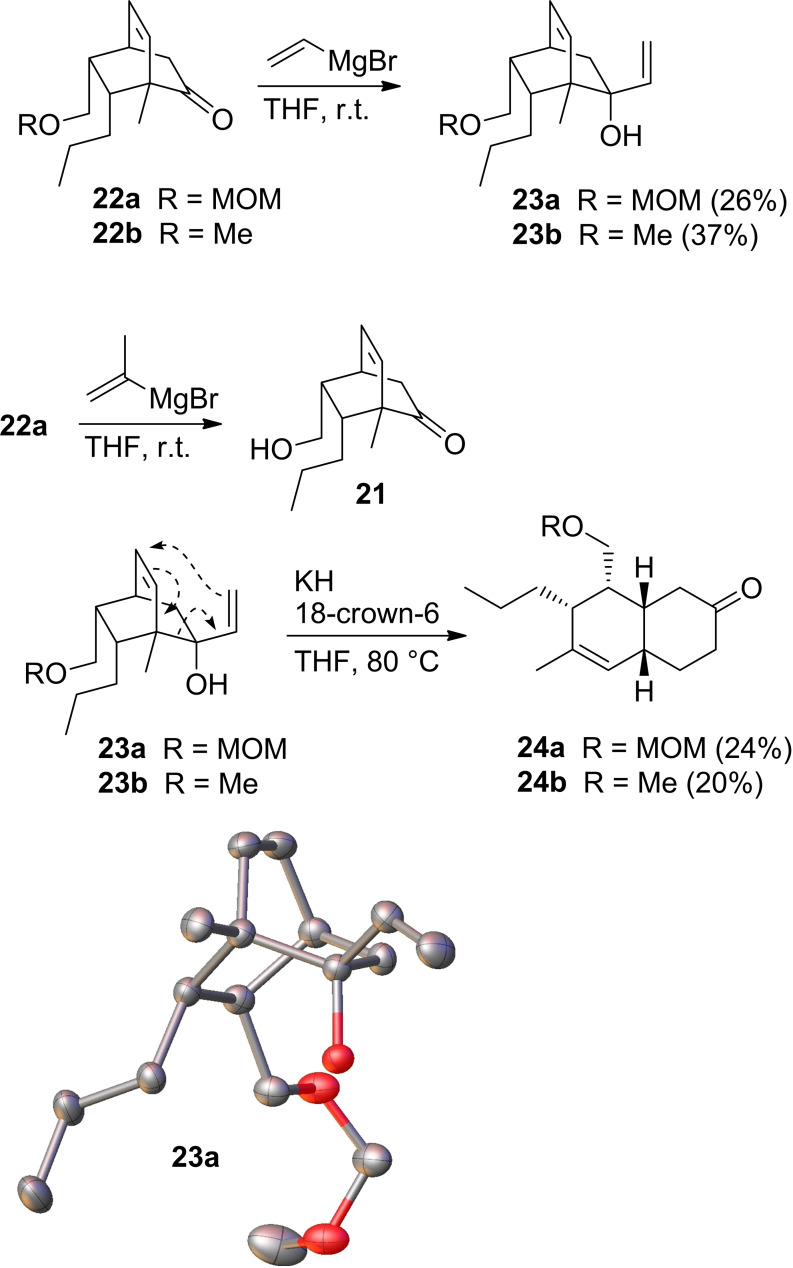
Attempts at Grignard reactions of ketones **22** with vinylmagnesium bromide and isopropenylmagnesium bromide. Oxy‐Cope rearrangement of the vinyl alcohols **23 a** and **23 b**. X‐ray structure of vinyl alcohol **23 a**. For clarity the hydrogen atoms are not shown. The picture was created with Olex2.^[29]^

With ketones **22** in hand we now focused on their reactions with vinylmagnesium bromide and isopropenylmagnesium bromide. However, as it turned out, the addition of isopropenylmagnesium bromide was not possible regardless of the ketone **22** being used. With the sterically less demanding vinylmagnesium bromide, the MOM and the Me protected hydroxymethylketone **22 a** and **22 b** reacted to the corresponding tertiary alcohols **23 a** and **23 b** in moderate yields (Scheme 4). Ketones **22 c** and **22 d** did not react at all with either one of the Grignard reagents. In both cases (**23 a** and **23 b**) only one diastereomer was formed. Namely, the one where the nucleophile had entered from the side of the C=C double bond. For **23 a**, an X‐structure analysis proved the assignment. In addition, the fact that we could successfully convert **23 a** and **23 b** to the decalinones **24 a** and **24 b**, respectively, supported the assignment. Unfortunately, the obtained yields in the oxy‐Cope rearrangement were only moderate, making a redesign of the route necessary.

**Scheme 5:**
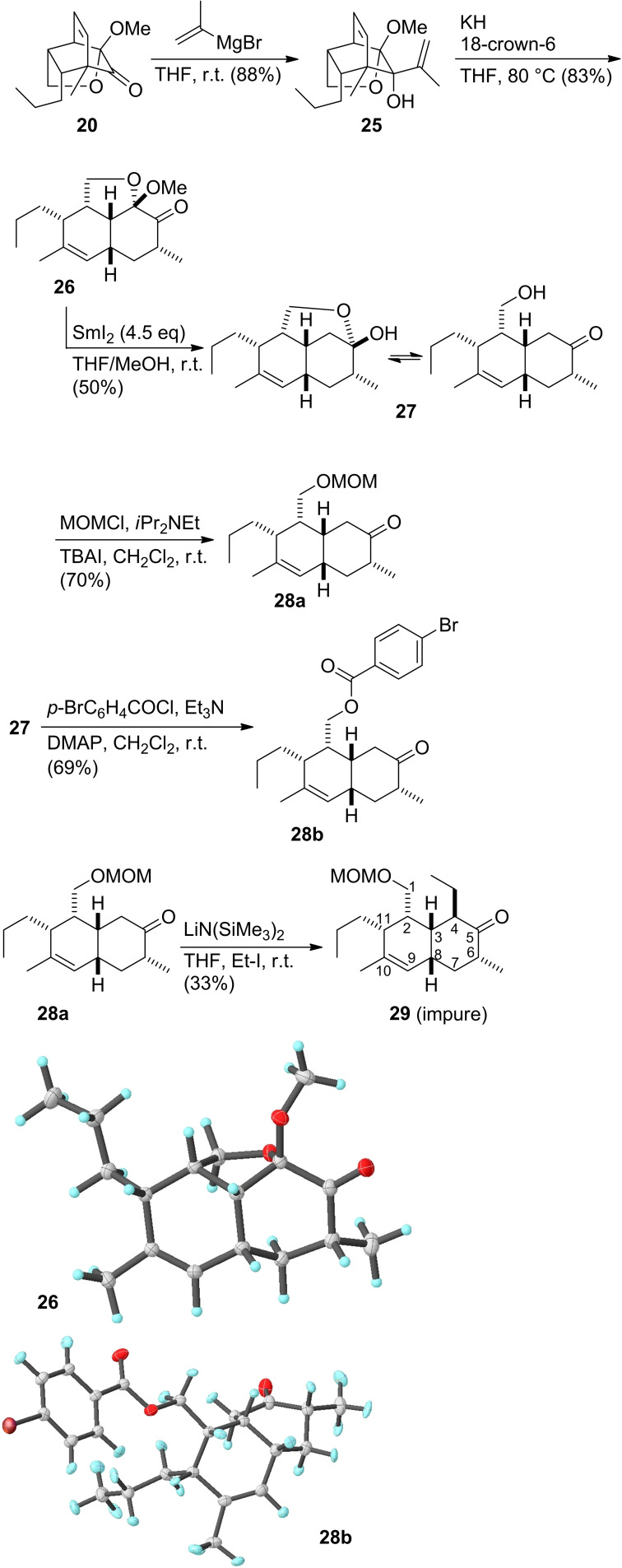
Modified strategy with Grignard addition to tricyclic ketone **20** followed by anionic oxy‐Cope rearrangement. X‐ray structures of **26** and **28 a**. Their rendering was created with Olex2.^[27]^

A simple change of the order of the steps brought about a solution to the problem. Thus, addition of isopropenylmagnesium bromide to tricyclic ketone **20** in THF proceeded in high yield, giving only the expected isomer **25** (Scheme 5). Most likely, the absence of acidic α‐methylene hydrogens contributes to the good yield in this transformation. The subsequent oxy‐Cope rearrangement using potassium hydride and 18‐crown‐6 delivered ketone **26** as a single isomer. Its structure was supported by an X‐ray analysis. The methyl group at C6 occupies a pseudo equatorial position. We now could address the crucial cleavage of the mixed acetal next to the keto function. This was possible under standard condition using an excess of samarium(II) iodide in a mixture of THF and methanol as proton source. This gave the hydroxmethyl ketone **27** in reasonable yield of 50 %. It seems that **27** is a mixture of at least two isomers. In the ^1^H and ^13^C NMR two set of signals can be observed. For example, there are two signals for 9‐H, one at 5.30 ppm, correlating with the ^13^C signal at 125.1 ppm. The other 9‐H signal resonates at δ=5.22 ppm, correlating with the 123.1 peak in the^13^C NMR. By integration a ratio of major/minor isomer of around 70 : 30 can estimated. The peak at 5.30 ppm belongs to the major isomer, most likely being the cyclic hemiacetal. In line with this, the ^13^C signal for the ketone at δ=216.2 ppm is rather small. There are also two peaks in the ^13^C of **27** at δ=63.6 (minor isomer) and 62.6 ppm (major isomer) that can be assigned to C1. The peak of the hemiacetal C is not visible in the ^13^C spectrum. Since the spectra of **27** were not completely clean, not all the peaks could be assigned unambiguously. Reaction of crude **27** with MOM chloride in presence of base provided the MOM protected hydroxymethylketone **28 a**. In a similar manner, **27** could be converted to *p*‐bromobenzoate **28 b** using *p*‐bromobenzoyl chloride and the combination of Et_3_N and DMAP. This derivative could be crystallized and examined by X‐ray analysis showing the relative stereochemistry on the hexahydronaphthalenone derivative (Scheme 5).

As a final challenge remained the selective introduction of an ethyl group at C4 (signermycin B numbering). Due to time constraints and the lack of larger amounts of substrate we tried the alkylation of **28 a** using LiN(SiMe_3_)_2_ in THF at room temperature. The obvious questions are, what is the regiochemistry of the enolate formation and what will be the stereochemistry at C4. We were able to isolate an ethylated derivative **29** in moderate yield. While **29** was not obtained completely pure, careful analysis of the 2D NMR spectra allowed for the following conclusions. Thus, the fact that the 6‐methyl group appears as a doublet (δ=1.02, *J*=6.6 Hz) indicated that enolate formation was regioselective. However, some key NOESY correlations indicate that alkylation at C4 had taken place from the β‐face of the decalin system. In particular, a correlation between 1‐H and 4‐H indicates that 4‐H is also on the α‐face of the decalinone. Moreover, the correlation 3‐H with 16‐H is in line with this assignment. Solutions to fix the stereochemistry issue might be enolization of **29** followed by protonation or aldol condensation of **28 a** with formaldehyde and reaction of the α‐methylene ketone with dimethyl cuprate.

## Conclusions

By using an intramolecular Diels‐Alder reaction of a masked *ortho*‐benzoquinone, generated in situ from phenol **17**, in presence of *cis*‐2‐hexenol (**18**), tricyclic ketone **19** was obtained. Following a palladium catalyzed reductive removal of the vinylic bromine, a Grignard reaction of ketone **20** with isopropenylmagnesium bromide led to dienol **25**. Via a key oxy‐Cope rearrangement, decalinone **26** with a *cis*‐fusion of the two six‐membered rings was obtained. The mixed acetal functionality next to the ketone of **26** was reduced to a methylene group with samarium iodide in methanol. After protection of the hydroxymethyl group as a MOM ether **28 a**, the core structure of signermycin B (**7**), was obtained. In a preliminary experiment, we could show that enolate formation on **28 a** was regioselective. However, alkylation of the enolate with ethyl iodide led to the undesired stereoisomer.

## Experimental Section

**General**. All reactions were performed under nitrogen atmosphere. All solvents used in the reactions were purified before use. The progress of the reactions was followed by TLC (POLYGRAM SIL G/UV254). Flash chromatography was performed on silica gel Silica M, 0.04–0.63 mm, from Machery‐Nagel GmbH & Co. KG, Germany. Distilled petroleum ether with a boiling range of 40–60 °C was used. Dry tetrahydrofuran and 1,4‐dioxane were distilled from sodium and benzophenone, whereas CH_2_Cl_2_ was distilled from CaH_2_. Methanol and DMF were used in HPLC grade quality. All commercially available compounds (abcr, Acros, Aldrich, Fluka, Merck and TCI) were used without purification. NMR spectra were recorded on a Bruker Avance III HD 400 (^1^H NMR: 400 MHz, ^13^C NMR: 101 MHz) and a Bruker Avance III HDX 600 (^1^H NMR: 600 MHz, ^13^C NMR: 151 MHz). CDCl_3_ was used as solvent at room temperature. The ^1^H NMR spectra were referenced to the residual signal of the non‐deuterated solvent component (CDCl_3_ 7.27 ppm) and the ^13^C NMR spectra to the signal of the deuterated solvent (CDCl_3_ 77.0 ppm). Peak assignments were made by NMR spectroscopy (^1^H, ^13^C, DEPT‐135, H,H‐COSY, HSQC, NOESY and HMBC). HRMS (ESI‐TOF) analysis were performed on a Bruker maXis 4G system.

Deposition numbers 2162972 (for **23 a**), 2224275 (for **26**) and 2208935 (for **28 b**) contain the supplementary crystallographic data for this paper. These data are provided free of charge by the joint Cambridge Crystallographic Data Centre and Fachinformationszentrum Karlsruhe.

## Conflict of Interests

The authors declare no conflict of interest.

1

## Supporting information

As a service to our authors and readers, this journal provides supporting information supplied by the authors. Such materials are peer reviewed and may be re‐organized for online delivery, but are not copy‐edited or typeset. Technical support issues arising from supporting information (other than missing files) should be addressed to the authors.

Supporting Information

## Data Availability

The data that support the findings of this study are available in the supplementary material of this article.
