# Supplementary material for: Approach to the Core Structure of Signermycin B
Source: ChemistryOpen. 2024 May 29;13(10):e202400103. doi: 10.1002/open.202400103 (PMC12056922; doi:10.1002/open.202400103)
Supplement: Supplementary file 1 — Supporting Information [file OPEN-13-e202400103-s001.pdf]

ChemistryOpen

Supporting Information

Approach to the Core Structure of Signermycin B

Khoa Linh Pham and Martin E. Maier*

Contents

General S2

Experimental procedures S2

NMR-Spectra S13

General

The numbering of the carbon framework of the compounds follows the IUPAC recommendations. For the decalinones, the numbering of the carbon framework follows that of the natural product.[Si1] **General**. All reactions were performed under nitrogen atmosphere. All solvents used in the reactions were purified before use. The progress of the reactions was followed by TLC (POLYGRAM SIL G/UV254). Flash chromatography was performed on silica gel Silica M, 0.04–0.63 mm, from Machery-Nagel GmbH & Co. KG, Germany. Distilled petroleum ether with a boiling range of 40−60 °C was used. Dry tetrahydrofuran and 1,4-dioxane were distilled from sodium and benzophenone, whereas CH_2Cl_2 was distilled from CaH_2. Methanol and DMF were used in HPLC grade quality. All commercially available compounds (abcr, Acros, Aldrich, Fluka, Merck and TCI) were used without purification. NMR spectra were recorded on a Bruker Avance III HD 400 (^{1}H NMR: 400 MHz, ^{13}C NMR: 101 MHz) and a Bruker Avance III HDX 600 (^{1}H NMR: 600 MHz, ^{13}C NMR: 151 MHz). $CDCl_3$ was used as solvent at room temperature. The ^{1}H NMR spectra were referenced to the residual signal of the non-deuterated solvent component ($CDCl_3$ 7.27 ppm) and the ^{13}C NMR spectra to the signal of the deuterated solvent ($CDCl_3$ 77.0 ppm). Peak assignments were made by NMR spectroscopy (^{1}H, ^{13}C, DEPT-135, H,H−COSY, HSQC, NOESY and HMBC). HRMS (ESI-TOF) analysis were performed on a Bruker maXis 4G system.

Experimental procedures

2-Methoxy-6-methylphenol[Si2] (**16**)

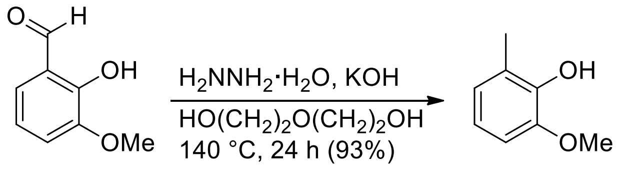

o-vanillin (**15**) **16**

As suspension of *o*-vanillin (10.00 g, 65.73 mmol, 1 equiv) in diethylene glycol (50 mL), was treated with hydrazine hydrate (6.58 g, 131.5 mmol, 2.0 equiv) and the mixture was heated to 110 °C. Now, KOH (22.13 g, 0.39 mol, 6.0 equiv) was added carefully and the orange reaction mixture brought to 140 °C and stirred at this temperature for 24 h. Thereafter, HCl (1N, 20 mL) was added and the cooled mixture extracted with CH_2Cl_2 (3 × 100 mL). The combined organic layers were dried over Na_2SO_4, filtered and concentrated in vacuo. The crude cresol derivative **16** (8.45 g, 61.23 mmol, 93%) was used in the next step without further purification. R_f = 0.63 (petroleum ether/ethyl acetate, 2:1); ^{1}H NMR (400 MHz, $CDCl_3$): δ = 2.28 (s, 3H, CH_3), 3.89 (s, 3H, OCH_3), 6.67–6.84 (m, 3H, Ar-H) ppm; ^{13}C-NMR (100 MHz, $CDCl_3$): δ = 15.4 (CH_3), 56.0 (OCH_3), 108.1, 119.1, 123.1, 123.9, 143.7, 146.2 (aryl C) ppm.

4-Brom-2-methoxy-6-methylphenol[Si3] (**17**)

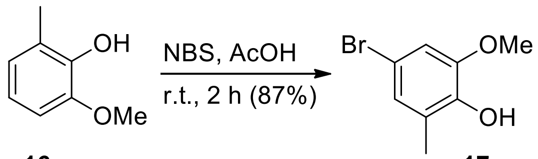

16 **17**

Cresol derivative **16** (10.28 g, 74.40 mmol, 1.0 equiv) was dissolved in AcOH (100 mL) and treated with NBS (13.90 g, 78.12 mmol, 1.05 equiv). The brown reaction mixture was stirred at room temperature for 2 h, then carefully treated with saturated $NaHCO_3$ solution (100 mL) followed by separation of the layers. The aqueous layer was extracted with CH_2Cl_2 (2 × 100mL). The combined organic layers were dried over Na_2SO_4, filtered and concentrated in vacuo. Aryl bromide **17** (14.11 g, 65.00 mmol, 87%) was obtained as an orange-

colored oil, which was used without further purification. R_f = 0.60 (petroleum ether/ethyl acetate, 2:1); ^{1}H NMR (400 MHz, $CDCl_3$): δ = 2.23 (s, 3H, CH_3), 3.87 (s, 3H, OCH_3),5.61 (s, 1H, OH), 6.82–6.85 (m, 1H, Ar-H), 6.88–6.92 (m, 1H, Ar-H) ppm; ^{13}C NMR (100 MHz, $CDCl_3$): δ = 15.2 (CH_3), 56.2 (OCH_3), 110.7, 111.6, 125.5, 125.7, 142.8, 146.7 (aryl C) ppm.

4-Bromo-7a-methoxy-6-methyl-8-propyl-3,3a,6,7a-tetrahydro-3,6-methanobenzofuran-7(2*H*)-one (**19**)

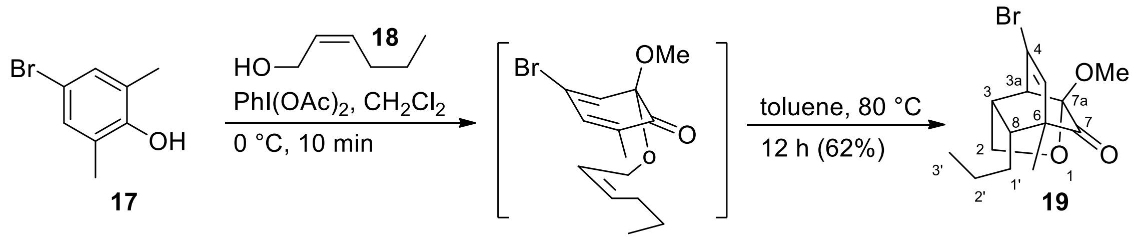

Diacetoxyiodobenzene (DAIB, 5.45 g, 16.91 mmol, 1.2 equiv) and *cis*-hexen-1-ol (**18**) (8.40 mL, 70.44 mmol, 5.0 equiv) were dissolved in dry CH_2Cl_2 (40 mL) and cooled to 0 °C. A solution of phenol **17** (3.06 g, 14.09 mmol, 1.0 equiv) in dry CH_2Cl_2 (20 mL) was added dropwise to the reaction mixture. A dark orange solution was formed. The reaction mixture was stirred for 10 min before the solvent was removed in vacuo. The concentrated mixture was redissolved in dry toluene (80 mL) and this mixture was stirred at 80 °C for 12 h. After cooling to room temperature, the mixture was washed with saturated $NaHCO_3$ solution (50 mL) and the layers were separated. The organic layer was dried over Na_2SO_4, filtered, and concentrated in vacuo. The crude cycloadduct was purified by flash chromatography (petroleum ether/ethyl acetate, 3:1) to give tricycle **19** (2.767 g, 8.78 mmol, 62%) as a colorless oil. R_f = 0.46 (petroleum ether/ethyl acetate, 3:1); ^{1}H NMR (400 MHz, $CDCl_3$): δ = 0.93 (t, *J* = 7.3 Hz, 2H, 3'-H), 0.99–1.08 (m, 1H, 1'-H), 1.15–1.24 (m, 1H, 2'-H), 1.23 (s, 3H, 6-CH_3), 1.29–1.35 (m, 1H, 2'-H), 1.50–1.57 (m, 1H, 1'-H), 1.69–1.73 (m, 1H, 8-H), 2.69–2.79 (m, 1H, 3-H), 3.55 (s, 3H, OCH_3), 3.57 (dd, *J* = 4.7, 2.7 Hz, 3a-H), 3.85–3.91 (m, 1H, 2-H), 3.99 (dd, *J* = 9.1, 3.9 Hz, 1H, 2-H), 6.14 (d, *J* = 2.7 Hz, 1H, 5-H) ppm; ^{13}C NMR (100 MHz, $CDCl_3$): δ = 14.1 (C-3'), 15.4 (6-CH_3), 21.0 (C-2'), 28.3 (C-1'), 38.3 (C-3), 43.6 (C-8), 51.6 (OCH_3), 51.9 (C-3a), 52.9 (C-6), 66.8 (C-2), 100.8 (C-7a), 118.1 (C-4), 136.5 (C-5), 200.8 (C-7) ppm; HRMS (ESI-TOF): calcd. for $C_{14}H_{19}BrO_3Na$ 337.04098 $[M+Na]^+$, found 337.04097.

7a-Methoxy-6-methyl-8-propyl-3,3a,6,7a-tetrahydro-3,6-methanobenzofuran-7(2*H*)-one (**20**)

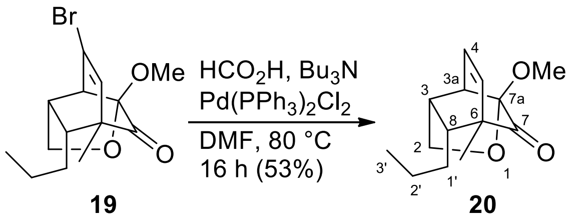

To a solution of bromoalkene **19** (4.92 g, 15.52 mmol, 1 equiv) in dry DMF (70 mL) were added sequentially Bu_3N (11.06 mL, 45.55 mmol, 3 equiv), HCO_2H (1.17 mL, 31.03 mmol, 2.0 equiv) and catalyst $Pd(PPh_3)_2Cl_2$ (544.53 mg, 775.79 µmol, 0.05 equiv). The reaction mixture was stirred at 80 °C for 16 h. After cooling, the reaction mixture was poured onto saturated $NaHCO_3$ solution (100 mL). The mixture was extracted with ethyl acetate (100 mL). The layers were separated and the organic layer was washed with saturated NaCl solution (100 mL). The organic layer was dried over Na_2SO_4, filtered, and concentrated in vacuo. The crude product was purified by flash chromatography (petroleum ether/ethyl acetate, 3:1) to give alkenone **20** (2.08 g, 8.28 mmol, 53%) as a colorless oil. R_f = 0.35 (petroleum ether/ethyl acetate, 3:1); ^{1}H NMR (400 MHz, $CDCl_3$): δ = 0.93 (t, *J* = 7.1 Hz, 3H, 3'-H), 1.01–1.11 (m, 1H, 1'H), 1.15–1.22 (m, 1H, 2'-H), 1.23 (s, 3H, 6-CH_3), 1.30–1.36 (m, 1H, 2'-H), 1.52–1.64 (m, 2H, 1'-H, 8-H), 2.46–2.56 (m, 1H, 3-H), 3.32–3.40 (m, 1H, 3a-H),

3.52 (s, 3H, OCH_3), 3.89 (d, *J* = 8.8 Hz, 1H, 2-H), 3.99 (dd, *J* = 3.9, 8.9 Hz, 1H, 2-H), 5.98 (dd, *J* = 1.9, 8.1 Hz, 1H, 5-H), 6.14 (dd, *J* = 6.6, 7.9 Hz, 1H, 4-H) ppm; ^{13}C NMR (100 MHz, $CDCl_3$): δ = 14.1 (C-3'), 15.4 (6-CH_3), 21.2 (C-2'), 28.5 (C-1'), 38.2 (C-3), 42.6 (C-3a), 43.7 (C-8), 50.6 (C-6), 51.3 (OCH_3), 67.4 (C-2), 100.9 (C-7a), 127.7 (C-4), 138.6 (C-5), 202.9 (C-7) ppm; HRMS (ESI-TOF): calcd. for $C_{14}H_{20}O_3Na$ 259.13047 $[M+Na]^+$, found 259.13084.

rel-(1*S*,4*R*,7*R*,8*R*)-8-(Hydroxymethyl)-1-methyl-7-propylbicyclo[2.2.2]oct-5-en-2-one (**21**)

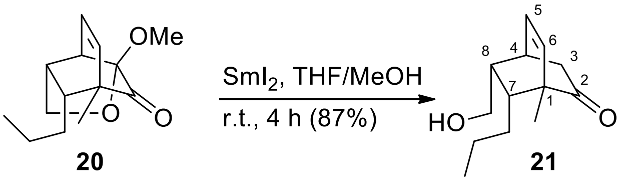

A solution of mixed acetal **20** (1.29 g, 5.45 mmol, 1 equiv) in dry MeOH (2.20 mL, 54.38 mmol, 10.0 equiv) was treated at room temperature with a solution of SmI_2 in dry THF[Si4] (0.1M, 245 mL, 4.5 equiv) followed by stirring the mixture at room temperature for 4 h. Most of the solvents from the brown reaction mixture were removed on the rotary evaporator and the remainder acidified with concentrated HCl (3 portions from a Pasteur pipette). The mixture was taken up with CH_2Cl_2 (200 mL), washed with saturated $NaHCO_3$ solution, saturated Na_2SO_3 solution and saturated NaCl solution (each 100 mL). The organic layer was dried over Na_2SO_4, filtered and concentrated in vacuo. Purification by flash chromatography (petroleum ether/ethyl acetate, 1:1) gave hydroxymethylketone **21** (996 mg, 4.79 mmol, 87%) as a colorless oil. R_f = 0.41 (petroleum ether/ethyl acetate, 1:1); ^1H NMR (400 MHz, $CDCl_3$): δ = 0.86 (t, *J* =7.3 Hz, 3H, 3'-H), 0.95–1.04 (m, 1H, 1'-H), 1.07–1.16 (m, 1H, 1'-H), 1.22 (s, 3H, 1-CH_3), 1.23–1.30 (m, 1H, 2'-H), 1.35–1.44 (m, 1H, 2'-H), 1.63–1.70 (m, 1H, 7-H), 1.97–2.05 (m, 1H, 3-H), 2.06–2.14 (m, 1H, 8-H), 2.19 (dd, *J* = 2.0, 18.6 Hz, 1H, 3-H), 3.06–3.10 (m, 1H, 4-H), 3.55–3.62 (m, 1H, CH_2OH), 3.94–3.98 (m, 1H, CH_2OH), 5.81 (dd, *J* = 1.2, 7.8 Hz, 1H, 6-H), 6.59 (dd, *J* = 7.5, 7.5 Hz, 1H, 5-H) ppm; ^{13}C NMR (100 MHz, $CDCl_3$): δ = 14.1 (C-3'), 16.3 (1-CH_3), 24.9 (C-2'), 28.1 (C-1'), 33.2 (C-4), 34.9 (C-3), 42.0 (C-7), 42.4 (C-8), 54.1 (C-1), 61.7 (CH_2OH), 134.4 (C-6), 138.5 (C-5), 212.8 (C-2) ppm; HRMS (ESI-TOF): calcd. for $C_{13}H_{20}O_2Na$ 231.13555 $[M+Na]^+$, found 231.13549.

rel-(1*S*,4*R*,7*R*,8*R*)-8-((Methoxymethoxy)methyl)-1-methyl-7-propylbicyclo[2.2.2]oct-5-en-2-one (**22a**)

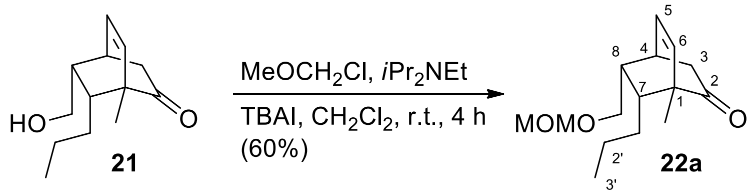

To a solution of alcohol **21** (1.71 g, 8.19 mmol, 1.0 equiv) in dry CH_2Cl_2 (10 mL) were added at 0 °C DIPEA (2.78 mL, 16.37 mmol, 2.0 equiv), TBAI (302.30 mg, 0.82 mmol, 0.1 equiv) and MOMCl (0.93 mL, 12.28 mmol, 1.50 equiv) one after the other followed by stirring of the mixture at room temperature for 4 h. The reaction mixture was quenched with saturated $NaHCO_3$ solution (10 mL) and extracted with diethyl ether (3 × 10 mL). The combined organic layers were washed with saturated NaCl solution (30 mL), dried over Na_2SO_4, filtered and concentrated in vacuo. After purification by flash chromatography (petroleum ether/ethyl acetate, 2:1), MOM ether **22a** (1.24 g, 4.92 mmol, 60%) was obtained as a colorless oil. R_f = 0.63 (petroleum ether/ethyl acetate, 1:1); ^1H NMR (400 MHz, $CDCl_3$): δ = 0.87 (t, *J* = 7.3 Hz, 3H, 3'-H), 1.02–1.14 (m, 2H, 1'-H), 1.23 (s, 3H, 1-CH_3), 1.21–1.28 (m, 1H, 2'-H), 1.37–1.44 (m, 1H, 2'-H), 1.64–1.71 (m 1H, 7-H), 1.97–2.04 (m, 1H, 3-H), 2.16–2.24 (m, 2H, 3-H, 8-H), 2.98–3.05 (m, 1H, 4-H), 3.38 (s, 3H, OCH_3), 3.50 (dd, *J* = 7.3, 12.6 Hz, 1H, CH_2OMOM), 3.75 (dd, *J* = 5.0, 9.7 Hz, 1H, CH_2OMOM), 4.63–4.68 (m, 2H, CH_2OCH_2OMe), 5.78–5.83 (dd, *J* = 8.0, 1.7 Hz, 1H, 6-H), 6.58 (dd, *J* = 7.0, 6.9 Hz, 1H, 5-H) ppm; ^{13}C NMR (100 MHz, $CDCl_3$): δ

= 14.1 (C-3'), 16.3 (1-CH_3), 25.0 (C-2'), 28.9 (C-1'), 33.9 (C-4), 34.9 (C-3), 39.8 (C-8), 42.0 (C-7), 54.0 (C-1), 55.3 (OCH_3), 66.9 (CH_2OMOM), 96.6 (CH_2OCH_2OMe), 134.4 (C-6), 138.5 (C-5), 212.6 (C-2) ppm; HRMS (ESI-TOF): calcd. for $C_{15}H_{24}O_3Na$ 275.16177 $[M+Na]^+$, found 275.16193.

rel-(1*S*,4*R*,7*R*,8*R*)-8-(Methoxymethyl)-1-methyl-7-propylbicyclo[2.2.2]oct-5-en-2-one (**22b**)

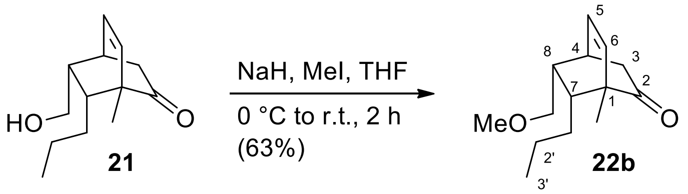

To a suspension of NaH (60%, in mineral oil, 100.23 mg, 4.18 mmol, 1.2 equiv) was added a solution of alcohol **21** (725.00 mg, 3.48 mmol, 1.0 equiv) in dry THF (5 mL) at 0 °C. Thereafter, MeI (0.21 mL, 3.31 mmol, 0.95 equiv) was added, followed by stirring of the mixture at 0 °C for 30 min and for a further 1.5 h at room temperature. The mixture was carefully treated with saturated NH_4Cl solution (5 mL). The layers were separated and the aqueous layer extracted with ethyl acetate (2 x 5 mL). The combined organic layers were dried over Na_2SO_4, filtered and concentrated in vacuo. After purification by flash chromatography (petroleum ether/ethyl acetate, 5:1), methyl ether **22b** (486.00 mg, 2.19 mmol, 63%) was obtained as a colorless oil. R_f = 0.40 (petroleum ether/ethyl acetate, 5:1); ^1H NMR (400 MHz, $CDCl_3$): δ = 0.87 (t, *J* = 7.2 Hz, 3H, 3'-H), 0.97–1.17 (m, 2H, 1'-H), 1.22 (s, 3H, 1-CH_3), 1.24–1.30 (m, 1H, 2'-H), 1.36–1.46 (m, 1H, 2'-H), 1.64–1.69 (m, 1H, 7-H), 1.94–2.04 (m 1H, 3-H), 2.11–2.22 (m, 2H, 3-H, 8-H), 2.95–3.00 (m, 1H, 4-H), 3.33 (dd, *J* = 8.8, 8.8 Hz, 1H, CH_2OMe), 3.37 (s, 3H, OCH_3), 3.57 (dd, *J* = 5.1, 9.3 Hz, 1H, CH_2OMe), 5.79 (dd, *J* = 8.0, 1.7 Hz, 1H, 6-H), 6.57 (dd, *J* = 6.9, 7.7 Hz, 1H, 5-H) ppm; ^{13}C NMR (100 MHz, $CDCl_3$): δ = 14.2 (C-3'), 16.3 (1-CH_3), 25.0 (C-2'), 28.8 (C-1'), 33.8 (C-4), 35.1 (C-3), 39.8 (C-8), 41.9 (C-7), 54.0 (C-1), 59.0 (OCH_3), 72.0 (CH_2OMe), 134.1 (C-6), 138.6 (C-5), 213.1 (C-2) ppm; HRMS (ESI-TOF): calcd. for $C_{14}H_{22}O_2Na$ 245.15120 $[M+Na]^+$, found 245.15145.

rel-(1*S*,4*R*,7*R*,8*R*)-8-(((*tert*-Butyldimethylsilyl)oxy)methyl)-1-methyl-7-propylbicyclo[2.2.2]oct-5-en-2-one (**22c**)

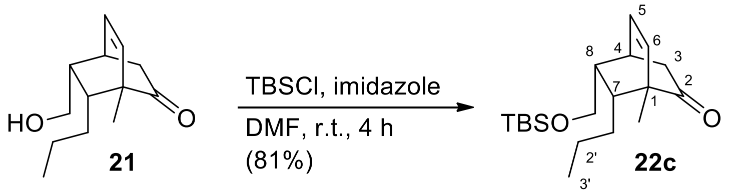

To a solution of alcohol **21** (854.00 mg, 4.10 mmol, 1.0 equiv) in dry DMF (10 mL) were added sequentially imidazole (32.68 mg, 480.07 µmol, 1.0 equiv) and TBSCl (79.59 mg, 528.08 µmol, 1.1 equiv) at room temperature followed by stirring of the mixture for 4 h at ambient temperature. The reaction mixture was quenched by adding water (20 mL) and extracted with ethyl acetate (2 x 20 mL). The combined organic layers were dried over Na_2SO_4, filtered and concentrated in vacuo. After purification by flash chromatography (petroleum ether/ethyl acetate, 9:1), silyl ether **22c** (1.066 g, 3.31 mmol, 81%) was obtained as a colorless oil. R_f = 0.37 (petroleum ether/ethyl acetate, 9:1); ^1H NMR (400 MHz, $CDCl_3$): δ = 0.07 (s, 3H, $Si(CH_3)_2$), 0.87 (t, *J* = 7.2 Hz, 3'-H), 0.90–0.92 (s, 9H, $SiC(CH_3)_3$), 1.01–1.13 (m, 2H, 1'-H), 1.21 (s, 3H, 1-CH_3), 1.25–1.43 (m, 2H, 2'-H), 1.61–1.67 (m, 1H, 7-H), 1.94–2.02 (m, 1H, 3-H), 2.03–2.10 (m,1H, 8-H), 2.19 (dd, *J* = 18.7, 2.0 Hz, 1H, 3-H), 3.01–3.05 (m, 1H, 4-H), 3.54 (dd, *J* = 10.0, 10.0 Hz, 1H, CH_2OTBS), 3.86 (dd, *J* = 10.3, 5.1 Hz, 1H, CH_2OTBS), 5.79 (dd, *J* = 7.6, 1.6 Hz, 1H, 6-H), 6.58 (dd, *J* = 7.6, 7.0 Hz, 1H, 5-H) ppm;

^{13}C NMR (100 MHz, $CDCl_3$): δ = –5.4 ($Si(CH_3)_2$), 14.1 (C-3'), 16.3 (1-CH_3), 18.3 (Si*C*$(CH_3)_3$), 25.1 (C-2'), 25.9 (SiC(*C*$H_3)_3$), 28.6 (C-1'), 33.5 (C-4), 35.1 (C-3), 42.1 (C-8), 42.2 (C-7), 54.1 (C-1), 62.1 (CH_2OTBS), 134.4 (C-6), 138.8 (C-5), 213.2 (C-2) ppm; HRMS (ESI-TOF): calcd. for $C_{19}H_{34}O_2SiNa$ 345.22203 $[M+Na]^+$, found 345.22173.

rel-(1*S*,4*R*,7*R*,8*R*)-1-Methyl-7-propyl-8-((trityloxy)methyl)bicyclo[2.2.2]oct-5-en-2-one (**22d**)

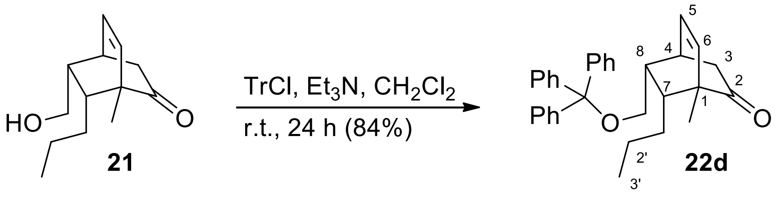

To a solution of alcohol **21** (866.00 mg, 4.16 mmol, 1.0 equiv) in dry CH_2Cl_2 (10 mL) were added NEt_3 (0.70 mL, 4.99 mmol, 1.2 equiv) and TrtCl (1.16 g, 4.16 mmol, 1.0 equiv). The reaction mixture was stirred at room temperature for 24 h and then poured into water (10 mL). The layers were separated and the aqueous layer was extracted with CH_2Cl_2 (2 x 10 mL). The combined organic layers were dried over Na_2SO_4, filtered, and concentrated in vacuo. The crude product was purified by flash chromatography (petroleum ether/ethyl acetate, 5:1) to give trityl ether **22d** as a colorless oil (1.57 g, 3.49 mmol, 84%). R_f = 0.57 (petroleum ether/ethyl acetate, 5:1); ^1H NMR (400 MHz, $CDCl_3$): δ = 0.56 (t, *J* =7.2 Hz, 3H, 3'-H), 0.77–1.00 (m, 4H, 1'-H, 2'-H), 1.08 (s, 3H, 1-CH_3), 1.48–1.54 (m, 1H, 7-H), 1.81–1.86 (m, 2H, 3-H), 2.18–2.19 (m, 1H, 8-H), 2.86–2.94 (m, 1H, 4-H), 3.16–3.19 (m, 1H, CH_2OTrt), 3.21–3.26 (m, 1H, 4-H), 5.70–5.74 (m, 1H, 6-H), 6.53–6.59 (m, 1H, 5-H), 7.21–7.25(m, 6H, Ar-H), 7.35–7.38 (m, 7H, Ar-H) ppm; ^{13}C NMR (100 MHz, $CDCl_3$): δ = 14.0 (C-3'), 16.3 (1-CH_3), 24.7 (C-2'), 28.7 (C-1'), 34.0 (C-4), 35.0 (C-3), 39.9 (C-8), 42.4 (C-7), 53.9 (C-1), 62.4 (CH_2OTrt), 86.7 (OC$(Ph)_3$), 127.0, 127.8, 128.6 (aryl C), 134.5 (C-6), 138.5 (C-5), 144.1 (aryl C), 212.9 (C-2).

rel-(1*S*,2*R*,4*R*,7*R*,8*R*)-8-((Methoxymethoxy)methyl)-1-methyl-7-propyl-2-vinylbicyclo[2.2.2]oct-5-en-2-ol (**23a**)

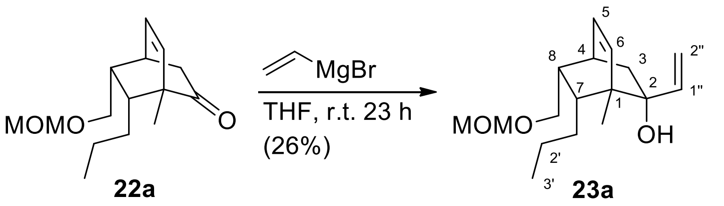

Vinylmagnesium bromide (1.90 mL, 1M in THF, 1.90 mmol, 3.05 equiv) was slowly added at 0 °C to a solution of ketone **22a** (155.0 mg, 0.614 mmol, 1.0 equiv) in dry THF (10 mL). The yellow reaction mixture was stirred at room temperature for 23 h before it was quenched with saturated NH_4Cl solution (100 mL) and extracted with ethyl acetate (100 mL). The organic layer was dried over Na_2SO_4, filtered, and concentrated in vacuo. After purification by flash chromatography (petroleum ether/ethyl acetate, 3:1), allyl alcohol **23a** (44.00 mg, 0.160 mmol, 26%) was obtained as colorless crystals. R_f = 0.39 (petroleum ether/ethyl acetate, 3:1); ^1H NMR (400 MHz, $CDCl_3$): δ = 0.90 (t, *J* = 6.9 Hz, 3H, 3'-H), 0.99 (s, 3H, 1-CH_3), 1.36–1.56 (m, 6H, 3-H, 1'-H, 2'-H), 1.88–1.94 (m, 1H, 7-H), 2.74–2.80 (m,1H, 4-H), 3.39 (s, 3H, OCH_3), 3.71–3.79 (m, 1H, CH_2OMOM), 3.88–3.95 (m, 1H,CH_2OMOM), 4.64–4.70 (m, 2H, OCH_2OMe), 4.94 (dd, *J* = 10.6, 1.4 Hz, 1H, 2''-H_{cis}), 5.08 (dd, *J* = 17.3, 1.3 Hz, 1H, 2''-H_{trans}), 5.70 (dd, *J* = 17.2, 10.6 Hz, 1H, 1''-H), 5.94 (dd, *J* = 8.1, 1.2 Hz, 1H, 6-H), 6.23–6.31 (m, 1H, 5-H) ppm; ^{13}C NMR (100 MHz, $CDCl_3$): δ = 14.3 (C-3'), 18.2 (1-CH_3), 25.0 (C-2'), 29.3 (C-1'), 32.5 (C-4), 35.2 (C-8), 38.1 (C-3), 44.1 (C-1), 45.1 (C-7), 55.4 (OCH_3), 67.4

(CH2OMOM), 78.6 (C-2), 96.7 (OCH2OMe), 109.2 (C-2''), 134.1 (C-5), 141.4 (C-6), 147.1 (C-1'') ppm; HRMS (ESI-TOF): calcd. for $C_{17}H_{28}O_3Na$ 303.19307 $[M+Na]^+$, found 303.19350.

rel-(1*S*,2*R*,4*R*,7*R*,8*R*)-8-(Methoxymethyl)-1-methyl-7-propyl-2-vinylbicyclo[2.2.2]oct-5-en-2-ol (**23b**)

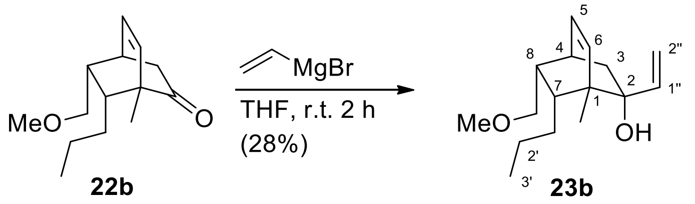

Vinylmagnesium bromide (7.21 mL, 1M in THF, 7.21 mmol, 3.0 equiv) was slowly added at 0 °C to a solution of ketone **22b** (527.00 mg, 2.37 mmol, 1.0 equiv) in dry THF (50 mL). The yellow reaction mixture was stirred at room temperature for 2 h before it was quenched with saturated NH_4Cl solution (30 mL) and extracted with ethyl acetate (30 mL). The organic layer was dried over Na_2SO_4, filtered, and concentrated in vacuo. After purification by flash chromatography (petroleum ether/ethyl acetate, 5:1), allyl alcohol **23b** (163.0 mg, 652 µmmol, 28%) was obtained as a colorless oil. R_f = 0.37 (petroleum ether/ethyl acetate, 5:1); ^{1}H NMR (400 MHz, $CDCl_3$): δ = 0.90 (t, *J* = 7.0 Hz, 3H, 3'-H), 0.98 (s, 3H, 1-CH_3), 1.34–1.56 (m, 5H, 1'-H, 2'-H, 3-H), 1.92 (dd, *J* = 14.1, 2.8 Hz 1H, 7-H), 2.02–2.14 (m, 2H, 3-H, 8-H), 2.60–2.75 (m, 1H, 4-H), 3.38 (s, 3H, OCH_3), 3.56–3.63 (m, 1H, CH_2OMe), 3.71–3.79 (m, 1H, 1''-H), 4.92 (dd, *J* = 10.8, 1.6 Hz, 1H, 2''-H), 5.09 (dd, *J* = 17.2, 1.5 Hz, 1H, 2''-H), 5.67 (dd, *J* = 17.2, 10.6 Hz, 1H, 1''-H), 5.93 (dd, *J* = 8.1, 1.2 Hz, 1H, 6-H), 6.21–6.29 (m, 1H, 5-H) ppm; ^{13}C NMR (100 MHz, $CDCl_3$): δ = 14.4 (C-3'), 18.2 (1-CH_3), 25.1 (C-2'), 29.4 (C-1'), 33.0 (C-4), 35.3 (C-8), 38.5 (C-3), 44.2 (C-1), 45.2 (C-7), 58.7 (OCH_3), 72.8 (CH_2OMe), 78.4 (C-2), 109.1 (C-2''), 134.3 (C-6), 141.3 (C-5), 147.0 (C-1'') ppm; HRMS (ESI-TOF): calcd. for $C_{16}H_{26}O_2Na$ 273.18250 $[M+Na]^+$, found 273.18250.

Hexahydronaphthalenone **24a**

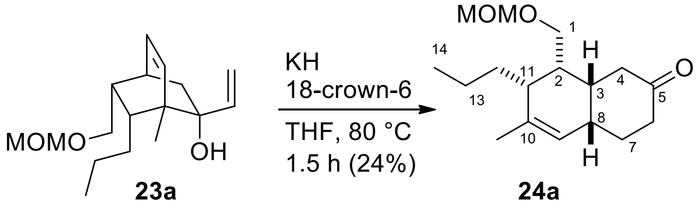

To a mixture of KH (30%, in mineral oil, 11.85 mg, 0.30 mmol, 2.0 equiv) and 18-crown-6 (78.12 mg, 0.3 mmol, 2.0 equiv) was added a solution of allyl alcohol **23a** (37.00 mg, 0.148 µmmol, 1.0 equiv) in dry THF (5 mL). The resulting light brown reaction mixture was heated to 80 °C for 1.5 h and then carefully poured into a saturated NH_4Cl solution (5 mL). The mixture was extracted with ethyl acetate (3x 5 mL) and the combined organic layers were dried over Na_2SO_4, filtered and concentrated in vacuo. After purification by flash chromatography (petroleum ether/ethyl acetate, 3:1), decalinone **24a** (10.00 mg, 35.00 µmol, 24%) was obtained as a colorless oil. R_f = 0.37 (petroleum ether/ethyl acetate, 3:1); ^{1}H NMR (400 MHz, $CDCl_3$): δ = 0.89 (t, *J* =7.0 Hz, 3H, 14-H), 1.13–1.44 (m, 4H, 12-H, 13-H), 1.79 (s, 3H, 10-CH_3), 1.89–1.96 (m, 2H, 7-H), 2.00–2.05 (m, 1H, 11-H), 2.18–2.21 (m, 2H, 4-H, 6-H), 2.27–2.37 (m, 4H, 2-H, 3-H, 4-H, 6-H), 2.49–2.55 (m, 1H, 8-H), 3.37 (s, 3H, OCH_3), 3.55–3.61 (m, 2H, 1-H), 4.59-4.70 (m, 2H, OCH_2OMe), 5.15–5.25 (m, 1H, 9-H) ppm; ^{13}C NMR (100 MHz, $CDCl_3$): δ = 14.4 (C-14), 22.5 (10-CH_3), 26.1 (C-13), 30.8 (C-7), 33.2 (C-12), 36.0 (C-8), 36.2 (C-2), 37.8 (C-6), 39.8 (C-4), 40.2 (C-11), 42.3 (C-3), 55.3 (OCH_3), 67.4 (C-1), 96.7 (OCH_2OMe), 122.8 (C-9), 139.8 (C-10), 212.6 (C-5) ppm; HRMS (ESI-TOF): calcd. for $C_{17}H_{28}O_3Na$ 303.19307 $[M+Na]^+$, found 303.19316.

Hexahydronaphthalenone **24b**

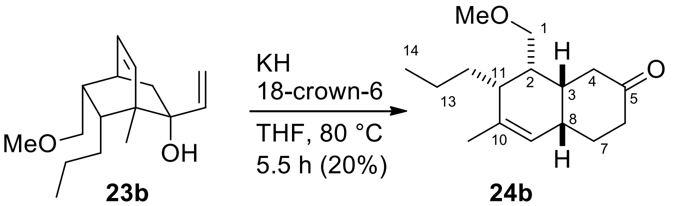

To a mixture of KH (30%, in mineral oil, 49.0 mg, 1.22 mmol, 2.0 equiv) and 18-crown-6 (323 mg, 1.22 mmol, 2.0 equiv) was added a solution of allyl alcohol **23b** (153.00 mg, 0.61 mmol, 1.0 equiv) in dry THF (8 mL). The resulting light brown reaction mixture was heated to 80 °C for 5.5 h and then carefully poured into a saturated NH_4Cl solution (10 mL). The mixture was extracted with ethyl acetate (3 x 10 mL) and the combined organic layers were dried over Na_2SO_4, filtered and concentrated in vacuo. After purification by flash chromatography (petroleum ether/ethyl acetate, 5:1), decalinone **24b** (30.00 mg, 0.12 mmol, 20%) was obtained as a colorless oil. R_f = 0.40 (petroleum ether/ethyl acetate, 5:1); ^{1}H NMR (400 MHz, $CDCl_3$): δ = 0.90 (t, *J* =7.0 Hz, 3H, 14-H), 1.18–1.27 (m, 1H, 12-H), 1.38–1.43 (m, 3H, 12-H, 13-H), 1.78 (s, 3H, 10-CH_3), 1.83–1.97 (m, 2H, 7-H), 2.00–2.05 (m, 1H, 11-H), 2.14–2.21 (m, 2H, 4-H, 6-H), 2.28–2.36 (m, 4H, 2-H, 3-H, 4-H, 6-H), 2.48–2.54 (m, 1H, 8-H), 3.34 (s, 3H, OCH_3), 3.37–3.45 (m, 2H, 1-H), 5.16–5.20 (s, 1H, 9-H) ppm; ^{13}C NMR (100 MHz, $CDCl_3$): δ = 14.4 (C-14), 22.5 (10-CH_3), 25.8 (C-13), 30.7 (C-7), 33.0 (C-12), 35.9 (C-8), 36.5 (C-2), 37.8 (C-6), 40.2 (C-11), 40.3 (C-4), 42.3 (C-3), 58.8 (OCH_3), 72.5 (C-1), 122.8 (C-9), 139.8 (C-10), 212.7 (C-5) ppm; HRMS (ESI-TOF): calcd. for $C_{16}H_{26}O_2Na$ 273.18250 $[M+Na]^+$, found 273.18252.

7a-Methoxy-6-methyl-7-(prop-1-en-2-yl)-8-propyl-2,3,3a,6,7,7a-hexahydro-3,6-methanobenzofuran-7-ol (**25**)

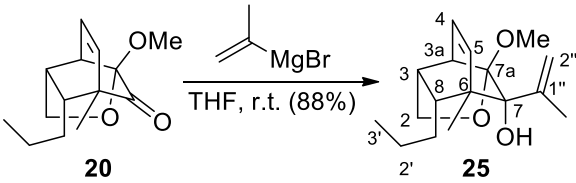

Propenylmagnesium bromide (49.5 mL, 0.5M in THF, 24.78 mmol, 3.0 equiv) was added dropwise at 0 °C to a solution of ketone **20** (1.95 g, 8.26 mmol, 1.0 equiv) in dry THF (100 mL) followed by stirring of the yellow reaction mixture at room temperature for 2 h. The reaction mixture was quenched by pouring it into a saturated aqueous NH_4Cl solution (50 mL). The mixture was extracted with ethyl acetate (2 × 50 mL). The combined organic layers were washed with saturated NaCl solution (100 mL), dried over Na_2SO_4, filtered and concentrated in vacuo. The brown crude product was purified by flash chromatography (petroleum ether/ethyl acetate, 5:1) to give allyl alcohol **25** (2.03 g, 7.31 mmol, 88%) as colorless crystals. R_f = 0.39 (petroleum ether/ethyl acetate, 5:1); ^{1}H NMR (400 MHz, $CDCl_3$): δ = 0.90 (t, *J* = 7.1Hz, 3H, 3'-H), 0.94–1.04 (m, 4H, 6-CH_3, 2'-H), 1.17–1.32 (m, 2H, 2'-H, 8-H), 1.63–1.71 (m, 5H, 1'-H, 1''-CH_3), 2.20–2.25 (m, 1H, 3-H), 3.22–3.27 (m, 1H, 3a-H), 3.34 (s, 3H, OCH_3), 3.93–4.02 (m, 2H, 2-H), 4.89–4.95 (m, 1H, 2''-H), 5.30 (bs, 1H, 2''-H), 5.81 (dd, *J* = 8.1, 6.6Hz, 1H, 4-H), 6.09 (dd, *J* = 8.2, 1.6 Hz, 1H, 5-H) ppm; ^{13}C NMR (100 MHz, $CDCl_3$): δ = 14.3 (C-3'), 17.0 (6-CH_3), 22.2 (C-2'), 25.6 (1''-CH_3), 29.5 (C-1'), 37.1 (C-3), 41.6 (C-3a), 44.9 (C-6), 47.4 (C-8), 51.1 (OCH_3), 66.8 (C-2), 84.8 (C-7), 111.4 (C-7a), 115.1 (C-2''), 121.1 (C-4), 145.2 (C-5), 146.5 (C-1'') ppm; HRMS (ESI-TOF): calcd. for $C_{17}H_{26}O_3Na$ 301.17742 $[M+Na]^+$, found 301.17801.

Decalinone **26**

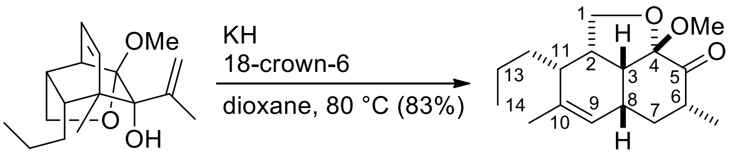

25 **26**

To a mixture of KH (30%, in mineral oil, 1.52 g, 38.02 mmol, 3.0 equiv) and 18-crown-6 (10.05 g, 38.02 mmol, 3.0 equiv) was added allyl alcohol **25** (3.53 g, 12.67 mmol, 1.0 equiv), dissolved in dry 1,4-dioxane (60 mL). The light brown reaction mixture was stirred at 80 °C for 2.5 h and then carefully poured into a saturated aqueous NH_4Cl solution (50 mL). The mixture was extracted with ethyl acetate (3 x 50 mL). The combined organic layers were dried over Na_2SO_4, filtered, and concentrated in vacuo. After purification by flash chromatography (petroleum ether/ethyl acetate, 8:1), decalinone **26** (2.91 g, 10.47 mmol, 83%) was obtained as colorless crystals. R_f = 0.46 (petroleum ether/ethyl acetate, 8:1); ^{1}H NMR (300 MHz, $CDCl_3$): δ = 0.97 (t, *J* = 7.3 Hz, 3H, 14-H), 0.97–1.02 (m, 1H, 12-H), 1.21 (d, *J* = 7.1 Hz, 3H, 6-CH_3), 1.26–1.44 (m, 2H, 13-H), 1.46–1.61 (m, 2H, 7-H, 13-H), 1.72 (d, *J* = 1.5 Hz, 3H, 10-CH_3), 1.75–1.80 (m, 1H, 12-H), 1.82–1.91 (m, 1H, 7-H), 2.25–2.48 (m, 4H, 3-H, 6-H, 8-H, 11-H), 3.03–3.13 (m, 1H, 2-H), 3.41 (s, 3H, OCH_3), 3.76 (dd, *J* = 11.2, 8.1 Hz, 1H, 1-H), 4.08 (t, *J* = 8.3 Hz, 1H, 1-H), 5.31 (bs, 1H, 9-H) ppm; ^{13}C NMR (100 MHz, $CDCl_3$): δ = 14.4 (C-14), 17.7 (6-CH_3), 21.0 (C-13), 21.7 (10-CH_3), 30.8 (C-8), 32.9 (C-12), 36.4 (C-6), 37.0 (C-7), 38.4 (C-2), 42.5 (C-11), 47.1 (C-3), 51.7 (OCH_3), 70.6 (C-1), 106.2 (C-4), 124.5 (C-9), 133.5 (C-10), 210.0 (C-5) ppm; HRMS (ESI-TOF): calcd. for $C_{17}H_{26}O_3Na$ 301.17742 $[M+Na]^+$, found 301.17784.

Hemiacetal **27**

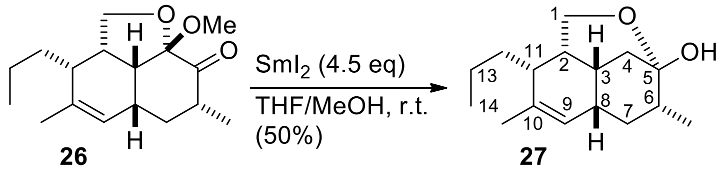

26 **27**

To a solution of acetal **26** (2.91 g, 10.46 mmol, 1.0 equiv) in dry MeOH (5.0 mL) was added a solution of SmI_2 (470.00 mL, 0.1M in THF, 47.00 mmol, 4.5 equiv)[Si4] followed by stirring the reaction mixture at room temperature for 23 h. The brown reaction mixture was then concentrated, and the residue taken up with CH_2Cl_2 (100 mL). This organic layer was then washed with water (100 mL), concentrated HCl (5 mL), saturated Na_2SO_3 solution (100 mL), saturated NaCl solution (100 mL), and dried over Na_2SO_4. After filtration and concentration in vacuo, the brown residue was purified by flash chromatography (petroleum ether/ethyl acetate, 2:1) to give hemicacetal **27** (1.316 g, 5.26 mmol, 50%) as a colorless oil. R_f = 0.40 (petroleum ether/ethyl acetate, 2:1); ^{1}H NMR (400 MHz, $CDCl_3$, major signals are listed): δ = 0.91 (t, *J* =7.0 Hz, 3H, 14-H), 1.04 (d, *J* = 6.7 Hz, 3H, 6-CH_3), 1.29–1.44 (m, 4H, 12-H, 13-H), 1.70 (s, 3H, 10-CH_3), 2.03–2.18 (m, 2H, 7-H), 2.32–2.38 (m, 1H, 7-H), 2.54–2.61 (m, 1H, 8-H), 3.72–3.79 (m, 2H, 13-H), 5.30 (s, 1H, 9-H) ppm; ^{13}C NMR (100 MHz, $CDCl_3$, major signals are listed): δ = 14.4 (C-14), 14.7 (6-CH_3), 22.3 (10-CH_3),26.4 (C-13), 33.1 (C-12), 34.1 (C-3), 37.4 (C-6, C-2), 39.7 (C-6, C-2), 39.8 (C-7), 40.6 (C-8), 40.8 (C-4), 45.2 (C-11), 62.6 (s, C-1), 63.6 (C-1), 123.1 (C-9), 125.1 (C-10) ppm; HRMS (ESI-TOF): calcd. for $C_{16}H_{26}O_2Na$ 273.18250 $[M+Na]^+$, found 273.18238.

Decalinone **28a**

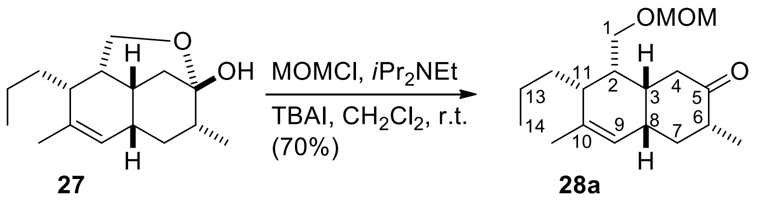

To a solution of alcohol **27** (446.00 mg, 1.78 mmol, 1.0 equiv) in dry CH_2Cl_2 (10 mL) were added at 0 °C DIPEA (0.60 mL, 3.56 mmol, 2.0 equiv), TBAI (65.80 mg, 0.18 mmol, 0.1 equiv) and MOMCl (0.20 mL, 2.67 mmol, 1.5 equiv) followed by stirring of the reaction mixture room temperature for 24 h. Thereafter, saturated $NaHCO_3$ solution (10 mL) was added and the mixture extracted with CH_2Cl_2 (3 x 10 mL). The combined organic layers were dried over Na_2SO_4, filtered, and concentrated in vacuo. After purification of the residue by flash chromatography (petroleum ether/ethyl acetate, 2:1), MOM ether **28a** (349.40 mg, 1.26 mmol, 70%) was obtained as a colorless oil. R_f = 0.60 (petroleum ether/ethyl acetate, 2:1); ^{1}H NMR (400 MHz, $CDCl_3$): δ = 0.90 (t, *J* = 7.3 Hz, 3H, 14-H), 1.06 (d, *J* = 6.7 Hz, 3H, 6-CH_3), 1.22–1.43 (m, 5H, 7-H, 12-H, 13-H), 1.73 (s, 3H, 10-CH_3), 1.99–2.08 (m, 2H, 7-H, 11-H), 2.17–2.25 (m, 1H, 2-H), 2.33–2.46 (m, 4H, 3-H, 4-H, 6-H), 2.63–2.72 (m, 1H, 8-H), 3.55 (s, 3H, OCH_3), 3.48–3.62 (m, 2H, 1-H), 4.54–4-61 (m, 2H, OCH_2OMe), 5.19 (bs, 1H, 9-H) ppm; ^{13}C NMR (100 MHz, $CDCl_3$): δ = 14.4 (C-14), 15.0 (6-CH_3), 22.6 (10-CH_3), 24.2 (C-13), 32.9 (C-12), 33.2 (C-6), 34.5 (C-8), 36.1 (C-7), 39.9 (C-4), 40.6 (C-2), 41.1 (C-11), 41.6 (C-3), 55.3 (OCH_3), 67.3 (C-1), 96.4 (OCH_2OMe), 124.2 (C-9), 136.6 (C-10), 214.6 (C-5) ppm; HRMS (ESI-TOF): calcd. for $C_{18}H_{30}O_3Na$ 317.20872 $[M+Na]^+$, found 317.20915.

Benzoate **28b**

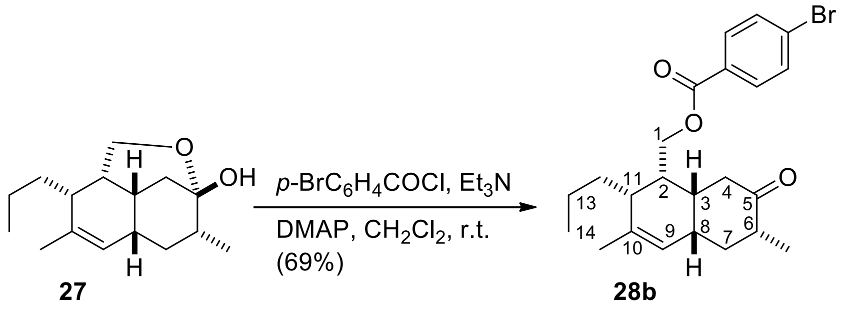

To a solution of alcohol **27** (215.00 mg, 0.859 mmol, 1.0 equiv) in dry CH_2Cl_2 (10 mL), DMAP (104.91 mg, 858.69 µmol, 1.0 equiv), NEt_3 (120.0 µL, 0.859 mmol, 1.0 equiv) and 4-bromobenzoyl chloride (226.12 mg, 1.03 mmol, 1.2 equiv) were added and the yellow reaction mixture was stirred at room temperature. After 2 h, the reaction mixture was quenched by adding water (10 mL), and extracted with CH_2Cl_2 (2 × 10 mL). The combined organic layers were dried over Na_2SO_4, filtered, and concentrated in vacuo. The yellow crude product was purified by flash chromatography (petroleum ether/ethyl acetate, 7:1) and benzoate **28b** (255.00 mg, 0.577 mmol, 69%) was obtained as a colorless oil, which crystallized from diethyl ether after slow evaporation of the solvent. R_f = 0.34 (petroleum ether/ethyl acetate, 7:1); ^{1}H NMR (400 MHz, $CDCl_3$): δ = 0.88 (t, *J* = 7.2 Hz, 3H, 14-H), 1.04 (d, *J* = 6.9 Hz, 3H, 6-CH_3), 1.26–1.48 (m, 5H, 7-H, 12-H, 13-H), 1.76 (s, 3H, 10-CH_3), 2.05–2.16 (m, 2H, 7-H, 11-H), 2.37–2.50 (m, 5H, 2-H, 3-H, 4-H, 6-H), 2.65–2.79 (m, 1H, 8-H), 4.28–4.44 (m, 2H, 1-H), 5.21 (s, 1H, 9-H), 7.53–7.64 (m, 2H, Ar-H), 7.81–7.95 (m, 2H, Ar-H) ppm; ^{13}C NMR (100 MHz, $CDCl_3$): δ = 14.4 (C-14), 15.0 (6-CH_3), 22.6 (10-CH_3), 24.5 (C-13), 32.9 (C-12), 33.4 (C-2), 34.4 (C-8), 36.0 (C-7), 39.4 (C-4), 40.1 (C-3), 40.7 (C-11), 41.5 (C-6), 64.9 (C-1), 124.1 (C-9), 128.1, 129.0, 131.1, 131.7 (aryl C), 136.4 (C-10), 165.7 (ArC=O), 214.5 (C-5) ppm; HRMS (ESI-TOF): calcd. for $C_{23}H_{29}BrO_3Na$ 455.11923 $[M+Na]^+$, found 455.11908.

Decalinone **29**

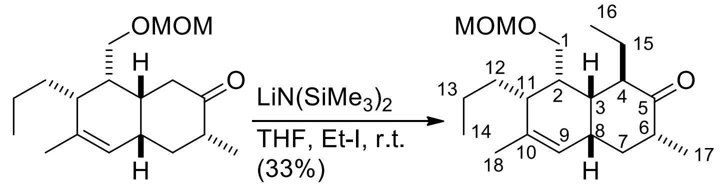

28a **29** (impure)

To a solution of ketone **28a** (112.00 mg, 402.25µmol, 1.00 equiv) in dry THF (2 mL) was added a solution of LiHMDS (1.0M in THF, 0.44 mL, 442.47 µmol, 1.10 equiv) dropwise at –78 °C. The yellow reaction mixture was stirred for 30 min at –78 °C before ethyl iodide (97.02 µL, 1.21 mmol, 3.00 equiv) was added and the mixture stirred for 30 min at –78 °C. Then, the mixture was warmed to room temperature and stirred at room temperature for 1.5 h. The reaction mixture was quenched by adding saturated NH_4Cl solution (2mL). The layers were separated and the aqueous layer was extracted with ethyl acetate (3 × 5 mL). The combined organic layers were dried over Na_2SO_4, filtered, and concentrated in vacuo. After purification by flash chromatography, alkylation product **29** (41.00 mg, 0.134 mmol, 33%) was obtained as a colorless oil. There were still slight impurities, possibly an isomer, that could not be separated off. R_f = 0.53 (petroleum ether/ethyl acetate, 4:1); ^{1}H NMR (600 MHz, $CDCl_3$): δ = 0.86–0.93 (m, 6H, 14-H, 16-H), 1.02 (d, *J* = 6.6 Hz, 17-H), 1.22–1.27 (m, 2H, 12-H, 13-H), 1.36–1.44 (m, 1H, 7-H), 1.51–1.61 (m, 3H, 12-H, 13-H, 15-H), 1.66–1.72 (m, 4H, 18-H, 15-H), 1.87–1.92 (m, 1H, 7-H), 2.13–2.21 (m, 2H, 2-H, 3-H), 2.17–2.20 (m, 1H, 3-H), 2.25–2.32 (m, 1H, 11-H), 2.37–2.44 (m, 2H, 4-H, 6-H), 2.57–2.65 (m, 1H, 8-H), 3.33 (s, 3H, OCH_3), 3.36–3.44 (m, 2H, 1-H), 4.43–4.50 (m, 2H, OCH_2OMe), 5.34–5.39 (m, 1H, 9-H) ppm; ^{13}C NMR (150 MHz, $CDCl_3$): δ = 12.4 (C-16), 14.4 (C-14), 15.1 (C-17), 20.8 (C-13), 21.7 (C-18), 27.0 (C-15), 31.3 (C-12), 33.5 (C-8), 38.5 (C-7), 40.2 (C-2), 41.1 (C-6), 43.0 (C-11), 45.1 (C-3), 55.3 (OCH_3), 55.7 (C-4), 65.7 (C-1), 96.1 (OCH_2OMe), 125.6 (C-9), 135.1 (C-10), 215.6 (C-5) ppm; HRMS (ESI-TOF): calcd. for $C_{20}H_{34}O_3Na$ 345.24002 $[M+Na]^+$, found 345.23995.

References

[Si1] T. Watanabe, M. Igarashi, T. Okajima, E. Ishii, H. Kino, M. Hatano, R. Sawa, M. Umekita, T. Kimura, S. Okamoto, Y. Eguchi, Y. Akamatsu, R. Utsumi, *Antimicrob. Agents Chemother.* **2012**, *56*, 3657-3663.

[Si2] R. V. Stevens, S. R. Angle, K. Kloc, K. F. Mak, Y. X. Liu, K. N. Trueblood, *J. Org. Chem.* **1986**, *51*, 4347-4353.

[Si3] C.-H. Lai, Y.-L. Shen, M.-N. Wang, N. S. Kameswara Rao, C.-C. Liao, *J. Org. Chem.* **2002**, *67*, 6493-6502.

[Si4] a) P. Girard, J. L. Namy, H. B. Kagan, *J. Am. Chem. Soc.* **1980**, *102*, 2693-2698; b) M. Szostak, M. Spain, D. J. Procter, *J. Org. Chem.* **2012**, *77*, 3049-3059.

NMR-Spectra

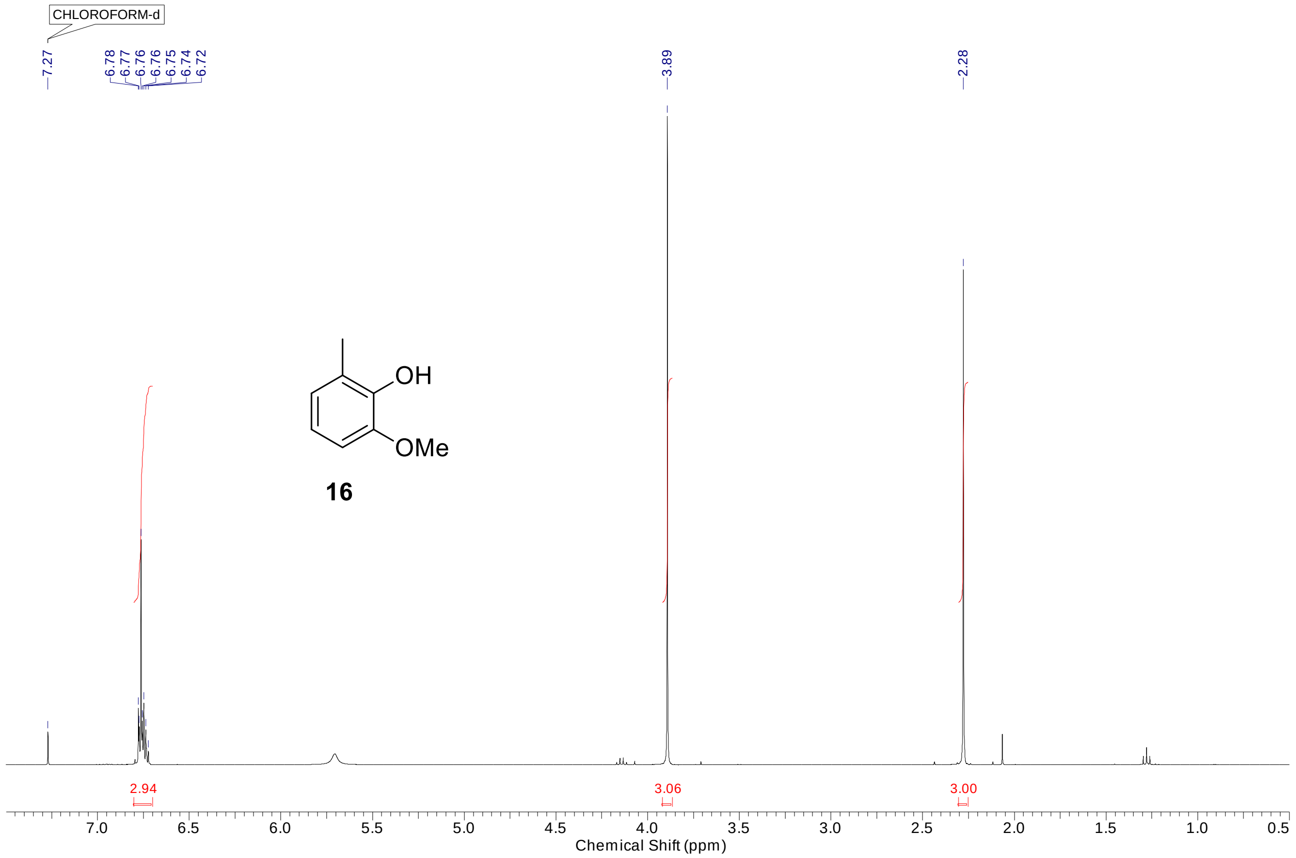

^{1}H NMR (400 MHz) spectrum of phenol **16** in $CDCl_3$ (0.5 – 7.5 ppm)

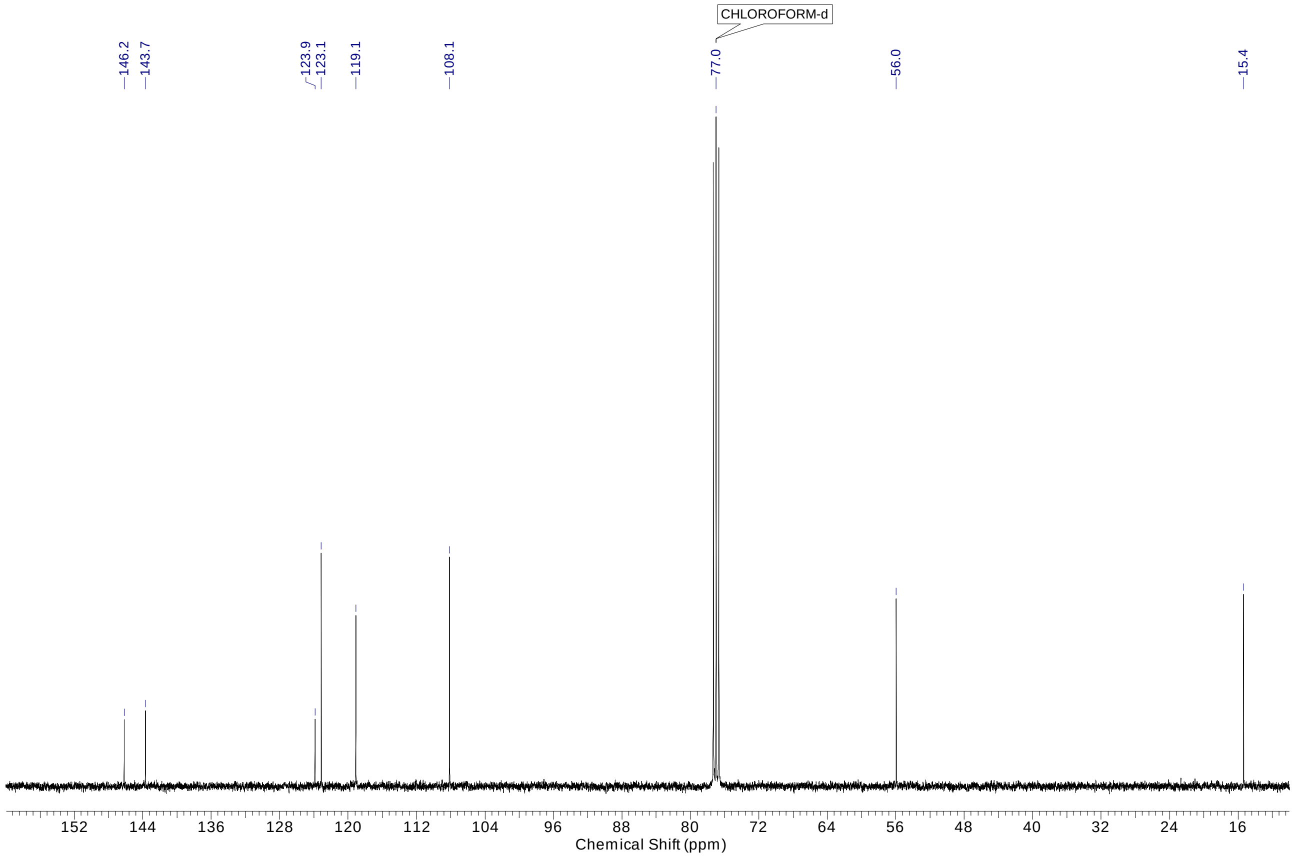

^{13}C NMR (100 MHz) spectrum of phenol **16** in $CDCl_3$ (10 – 160 ppm)

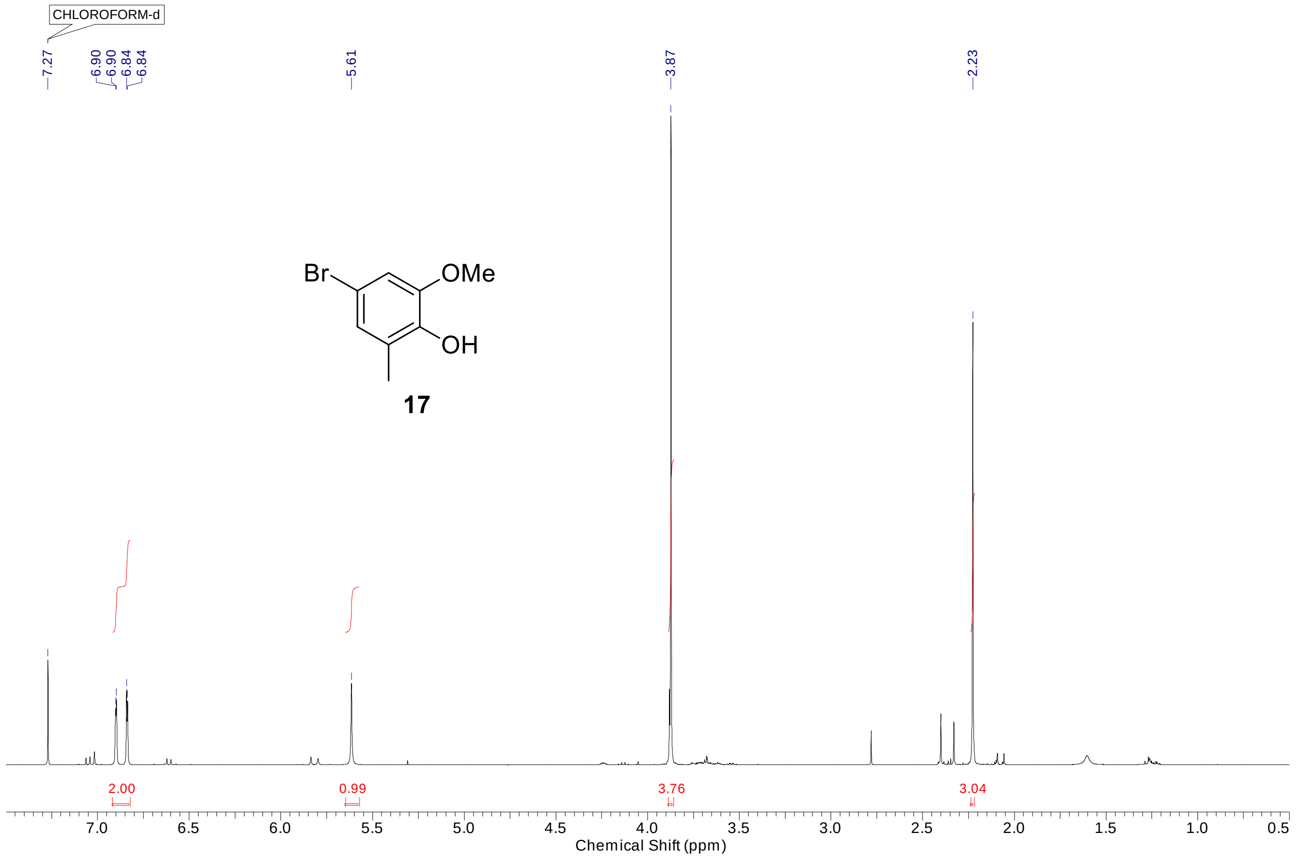

^{1}H NMR (400 MHz) spectrum of 4-bromophenol **17** in $CDCl_3$ (0.5 – 7.5 ppm)

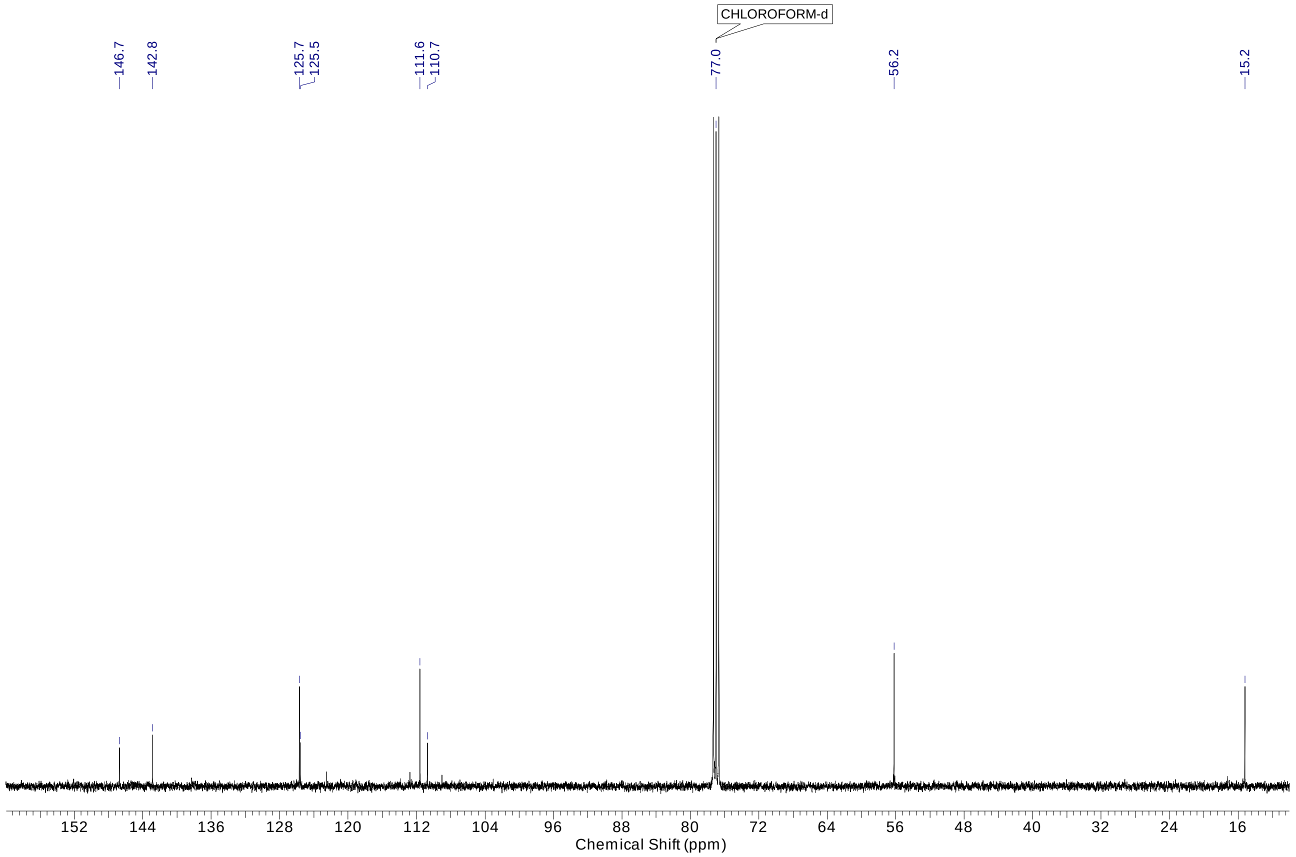

^{13}C NMR (100 MHz) spectrum of bromophenol **17** in $CDCl_3$ (10 – 160 ppm)

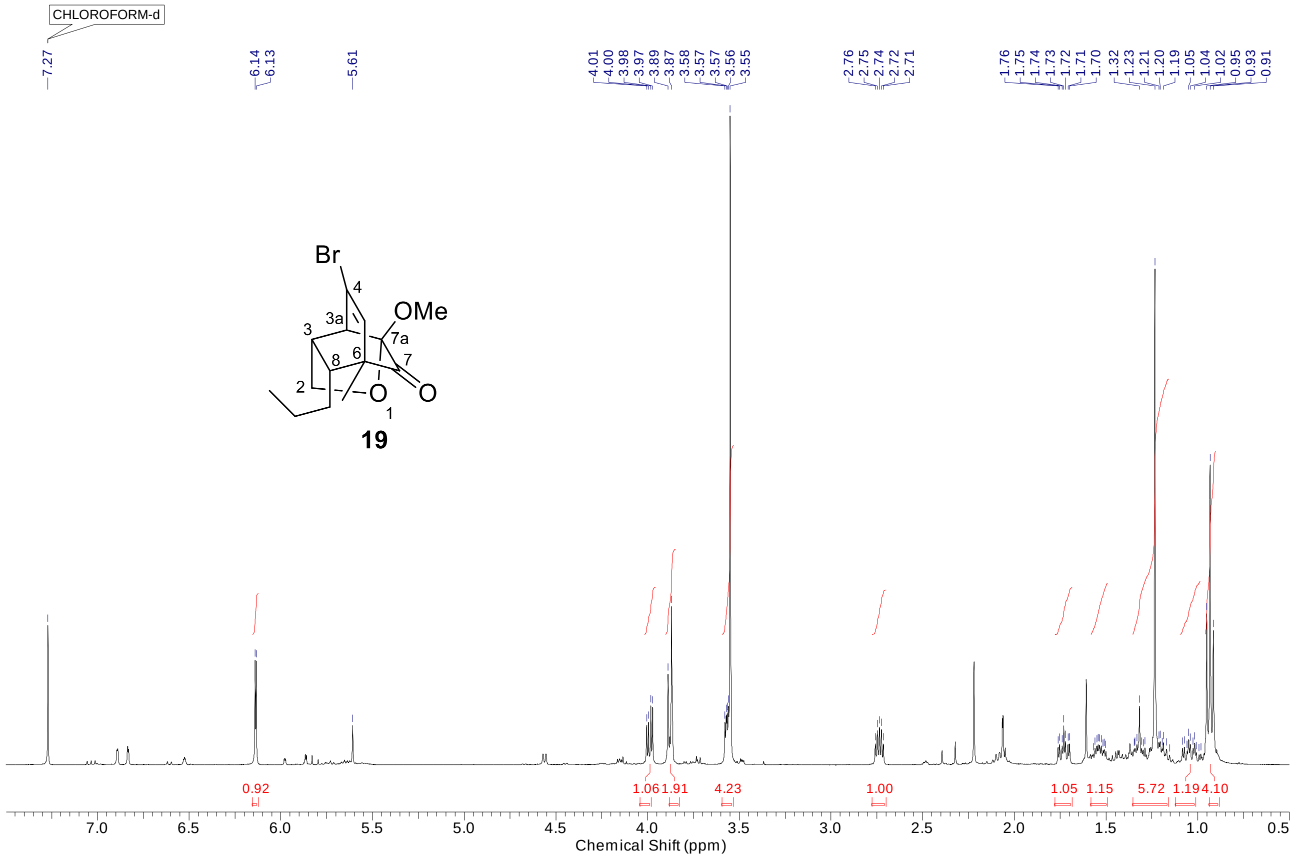

^{1}H NMR (400 MHz) spectrum of cycloadduct **19** in $CDCl_3$ (0.5 – 7.5 ppm)

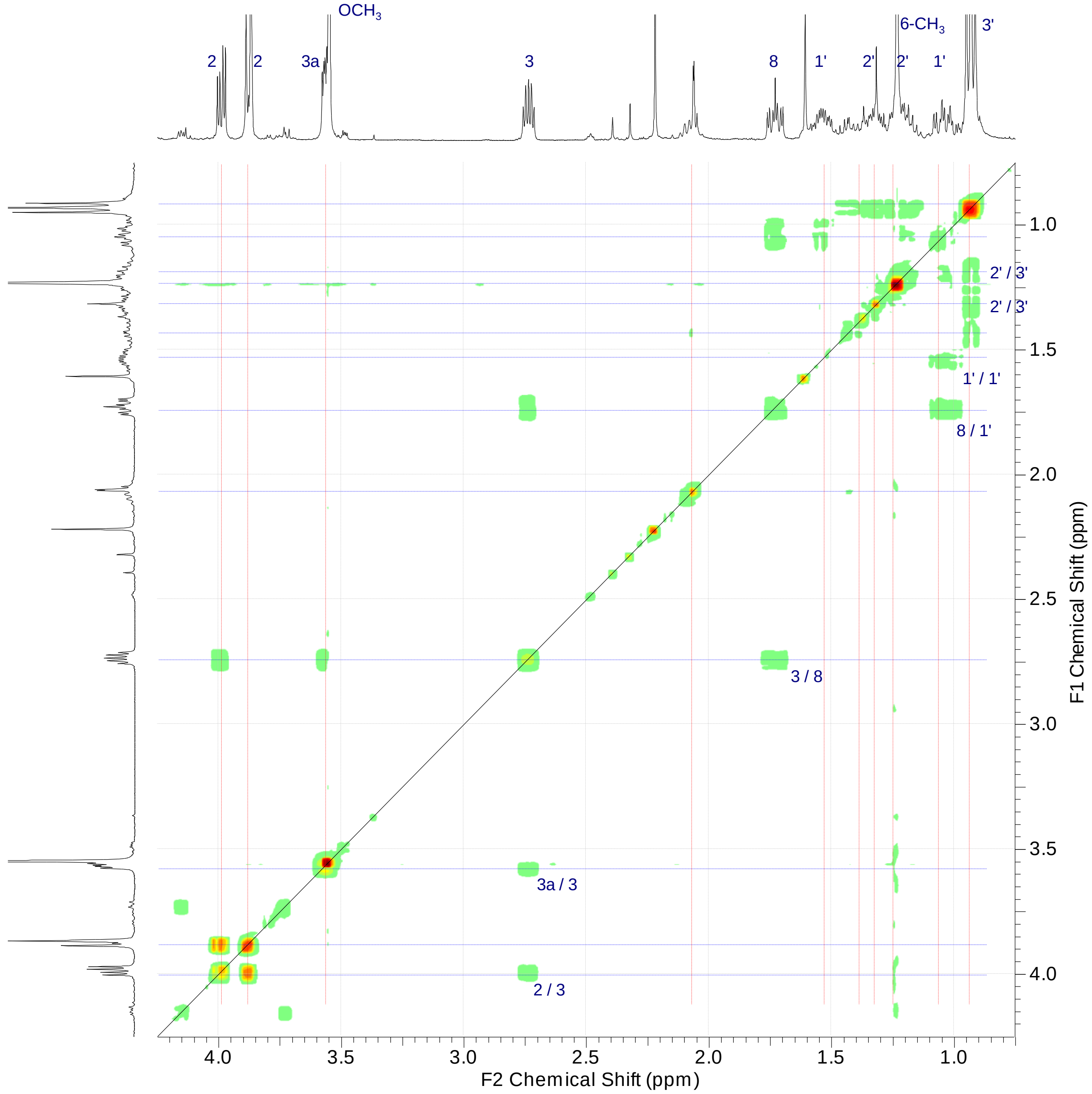

COSY spectrum (400 MHz) of cycloadduct **19** in $CDCl_3$ (0.75 – 4.25 ppm)

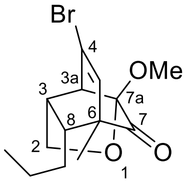

19

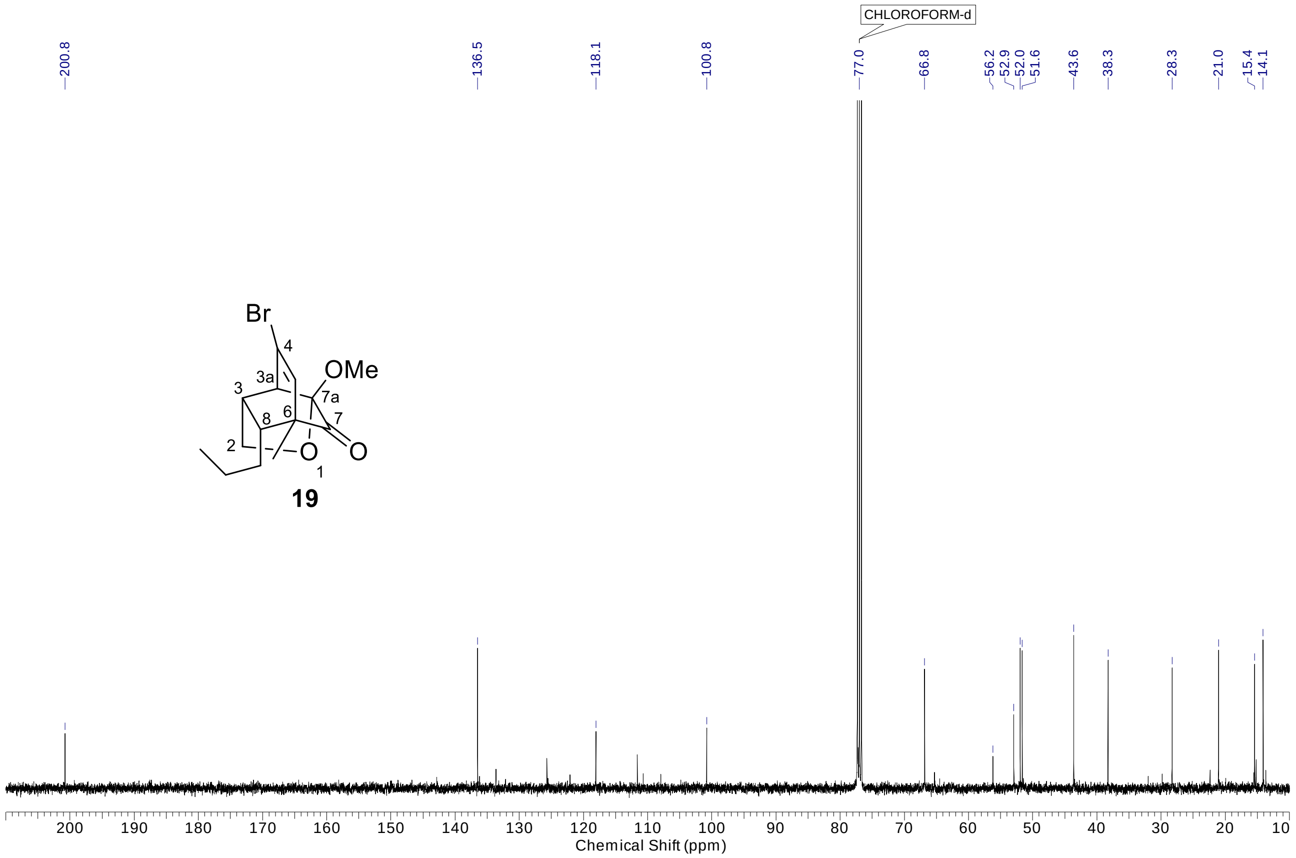

^{13}C NMR (100 MHz) spectrum of cycloadduct **19** in $CDCl_3$ (10 – 210 ppm)

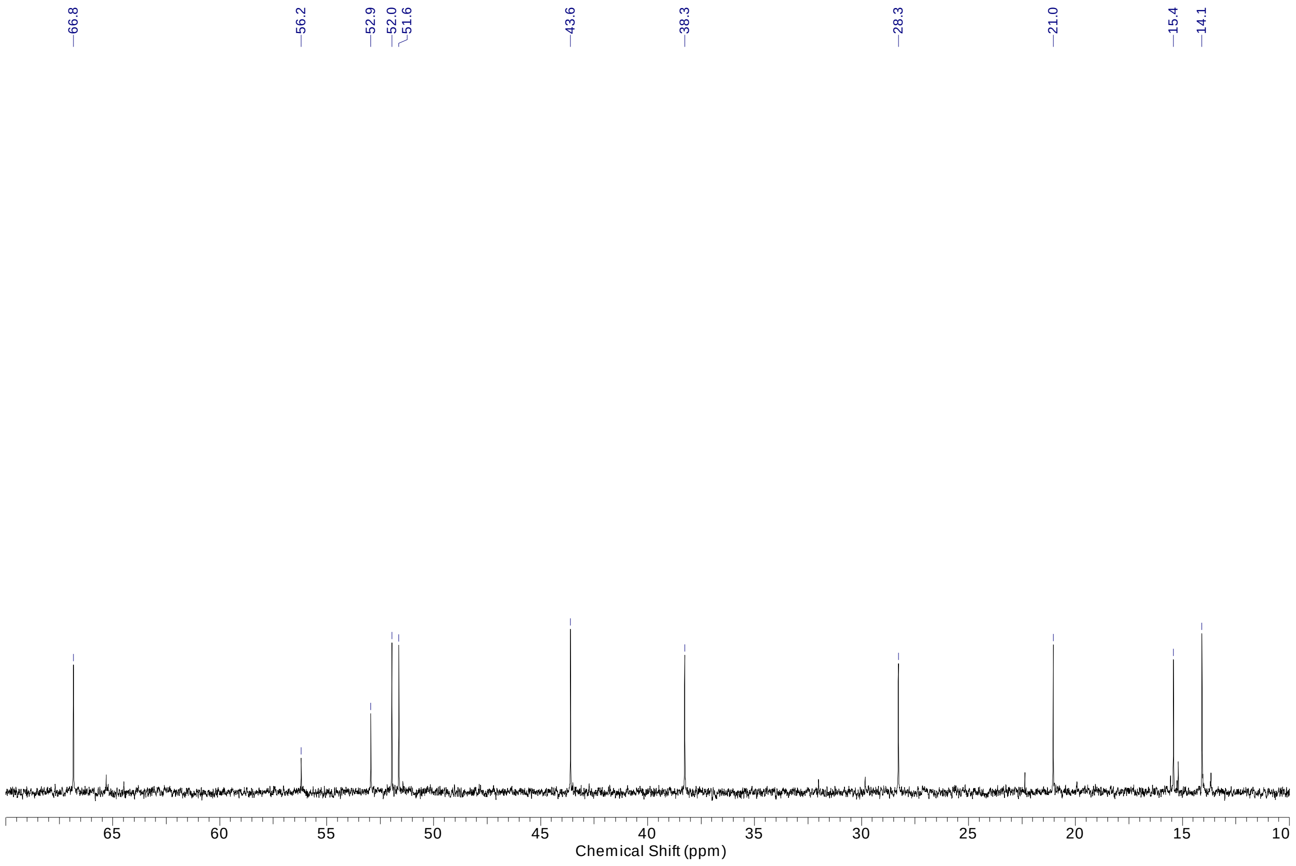

^{13}C NMR (100 MHz) spectrum of cycloadduct **19** in $CDCl_3$ (10 – 70 ppm)

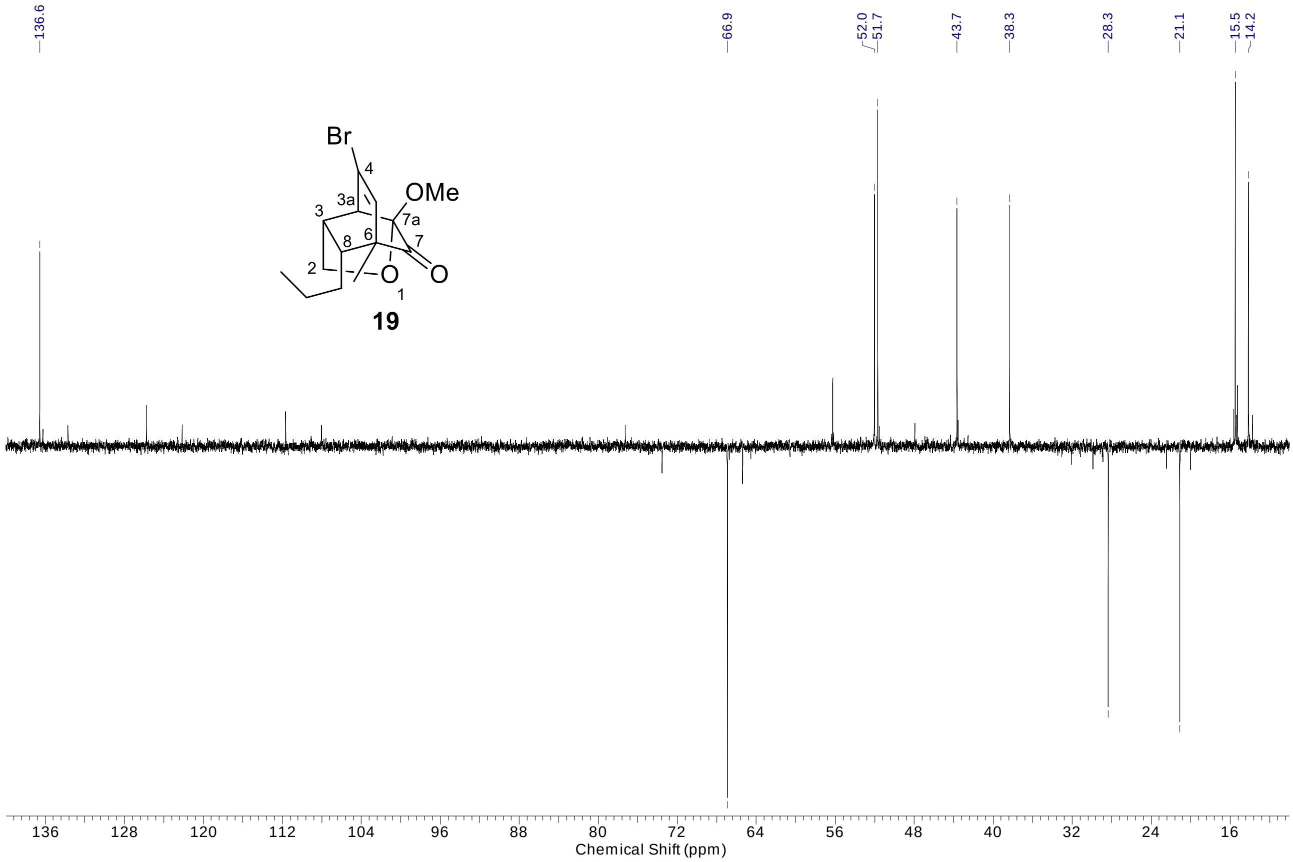

DEPT (100 MHz) spectrum of cycloadduct **19** in $CDCl_3$ (10 – 70 ppm)

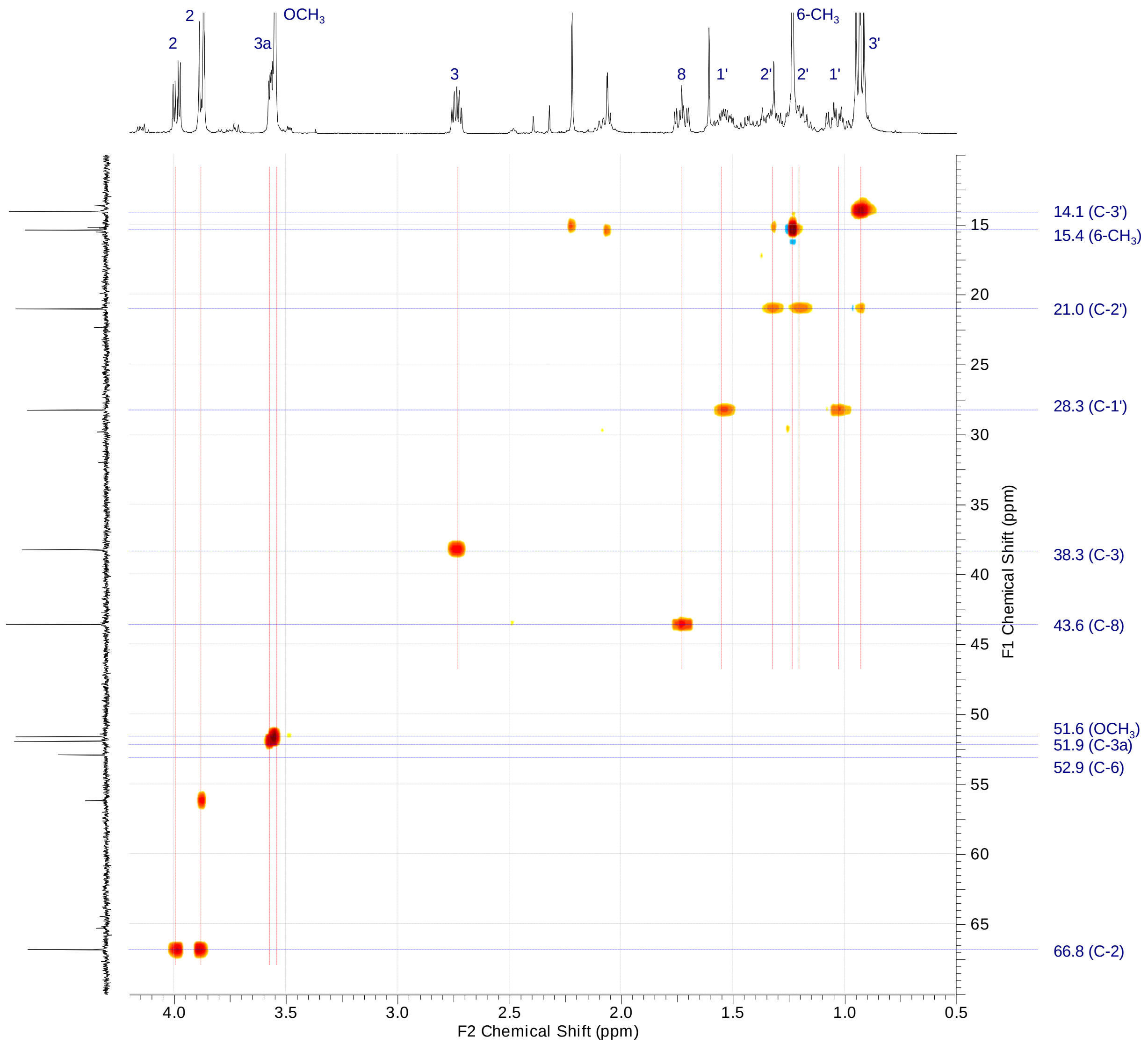

HSQC spectrum of cycloadduct **19** in $CDCl_3$ (0.5 – 4.2, 10 – 70 ppm)

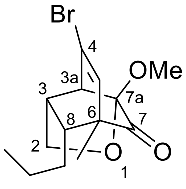

19

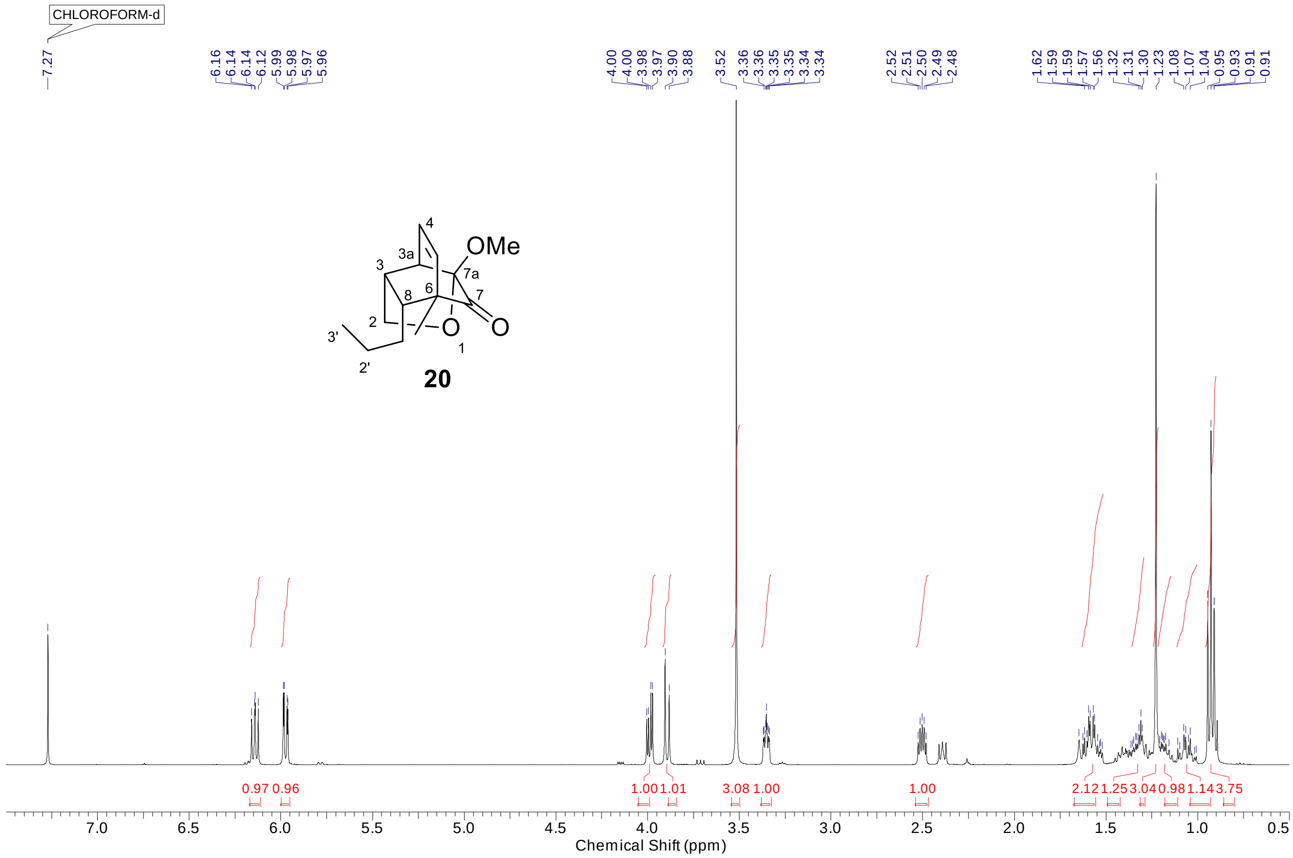

^{1}H NMR (400 MHz) spectrum of polycycle **20** in $CDCl_3$ (0.5 – 7.5 ppm)

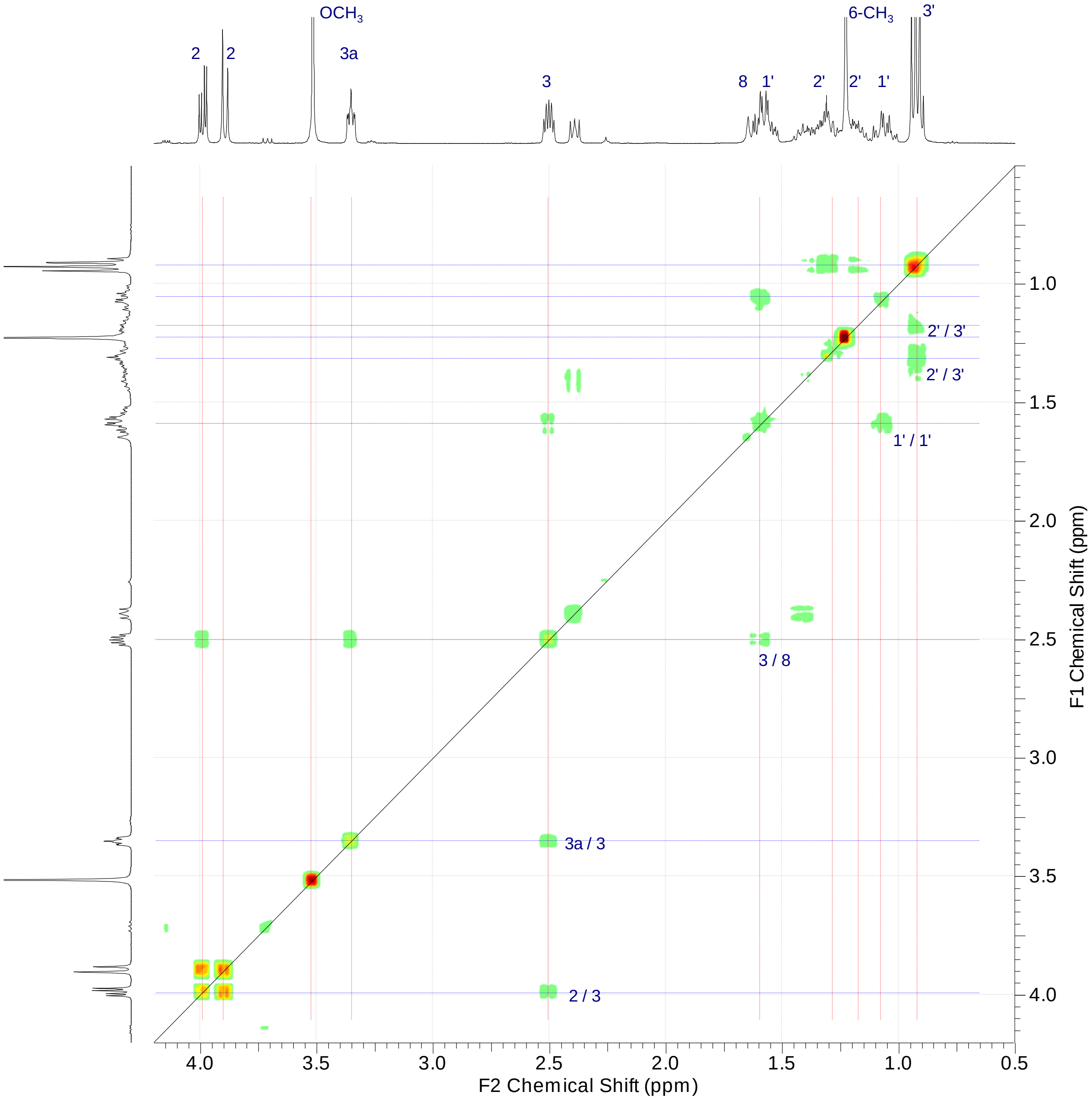

COSY spectrum (400 MHz) of polycycle **20** in $CDCl_3$ (0.5 – 4.2 ppm)

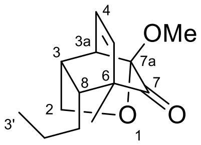

20

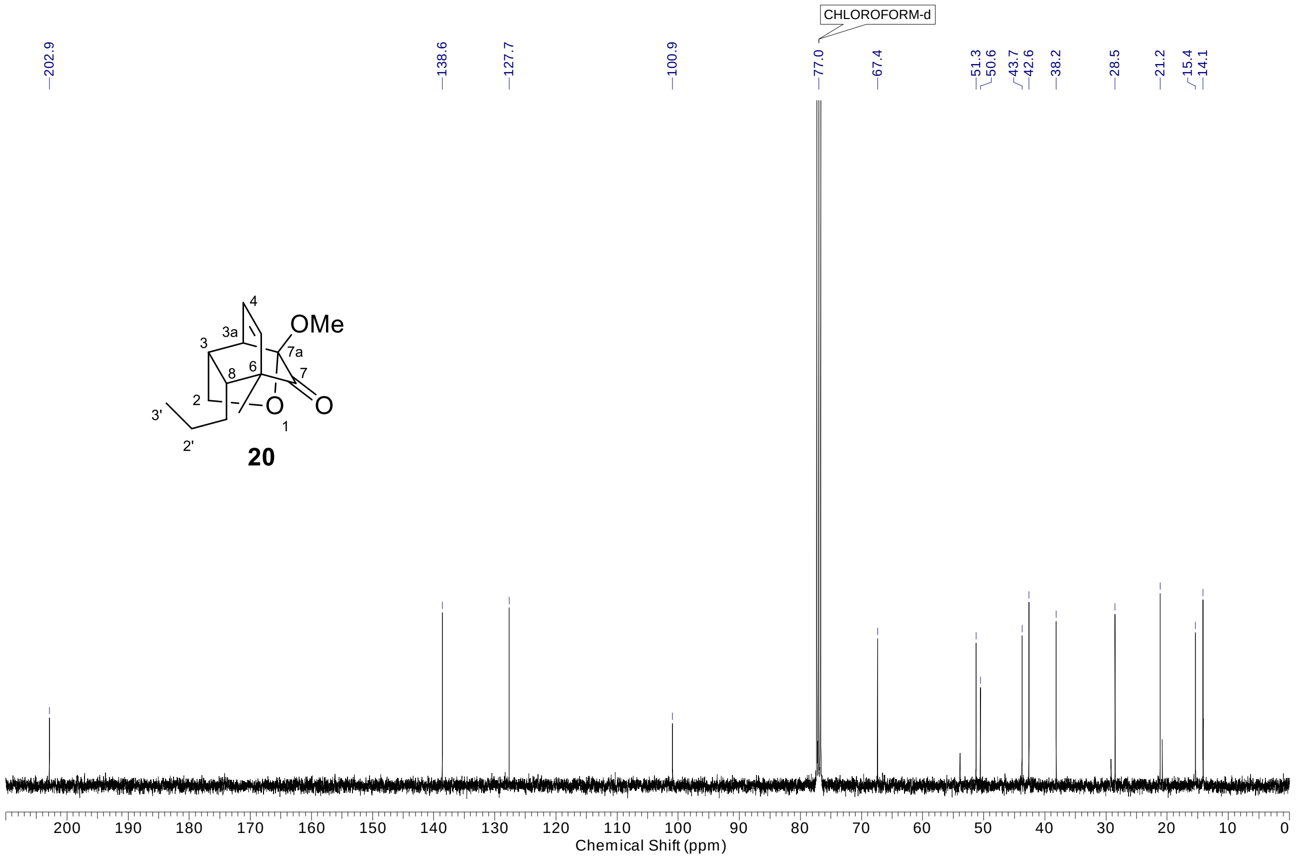

^{13}C NMR (100 MHz) spectrum of polycycle **20** in $CDCl_3$ (0 – 210 ppm)

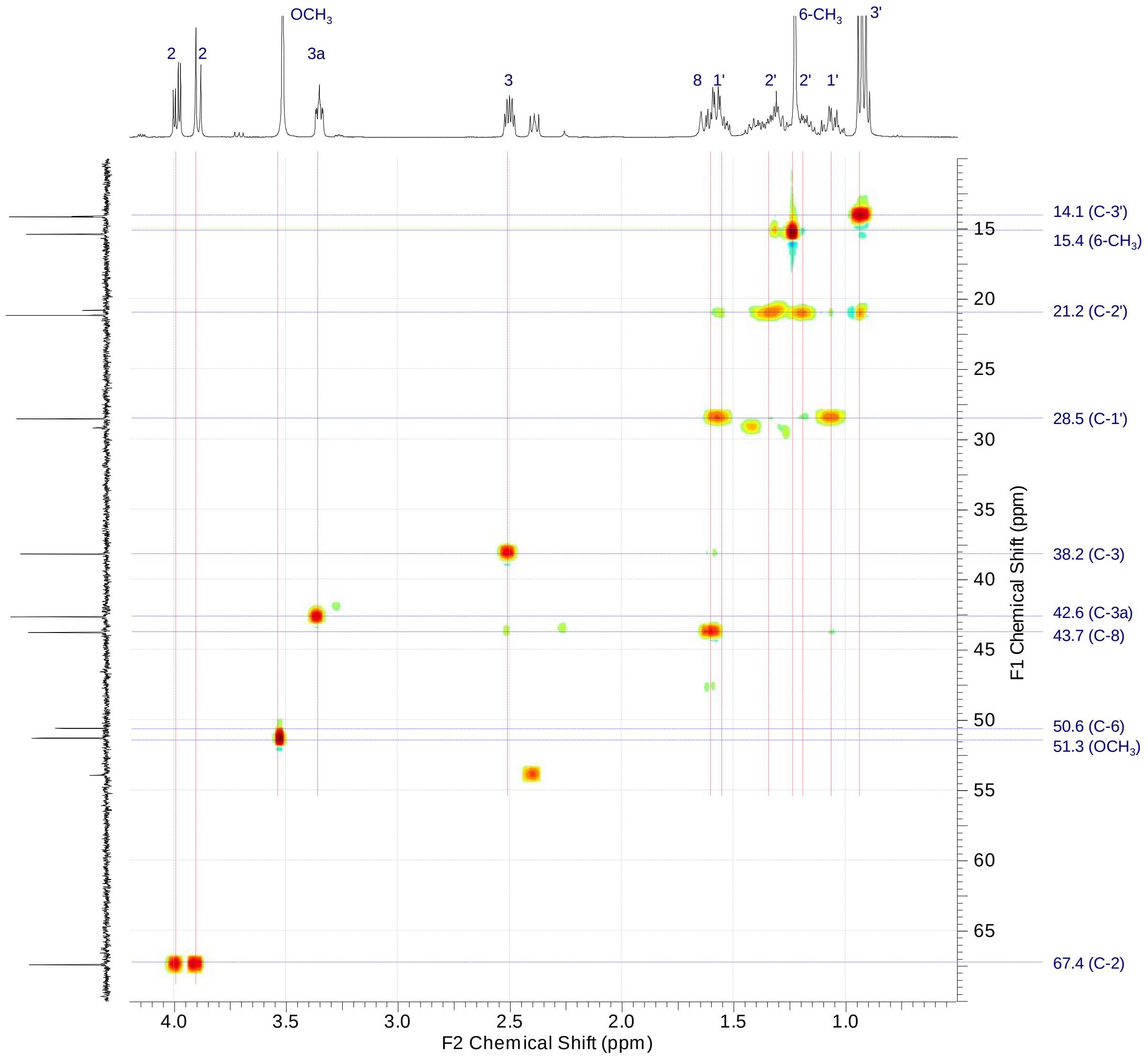

HSQC spectrum of polycycle **20** in $CDCl_3$ (0.5 – 4.2, 10 – 70 ppm)

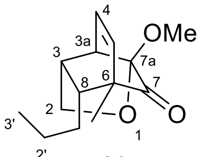

20

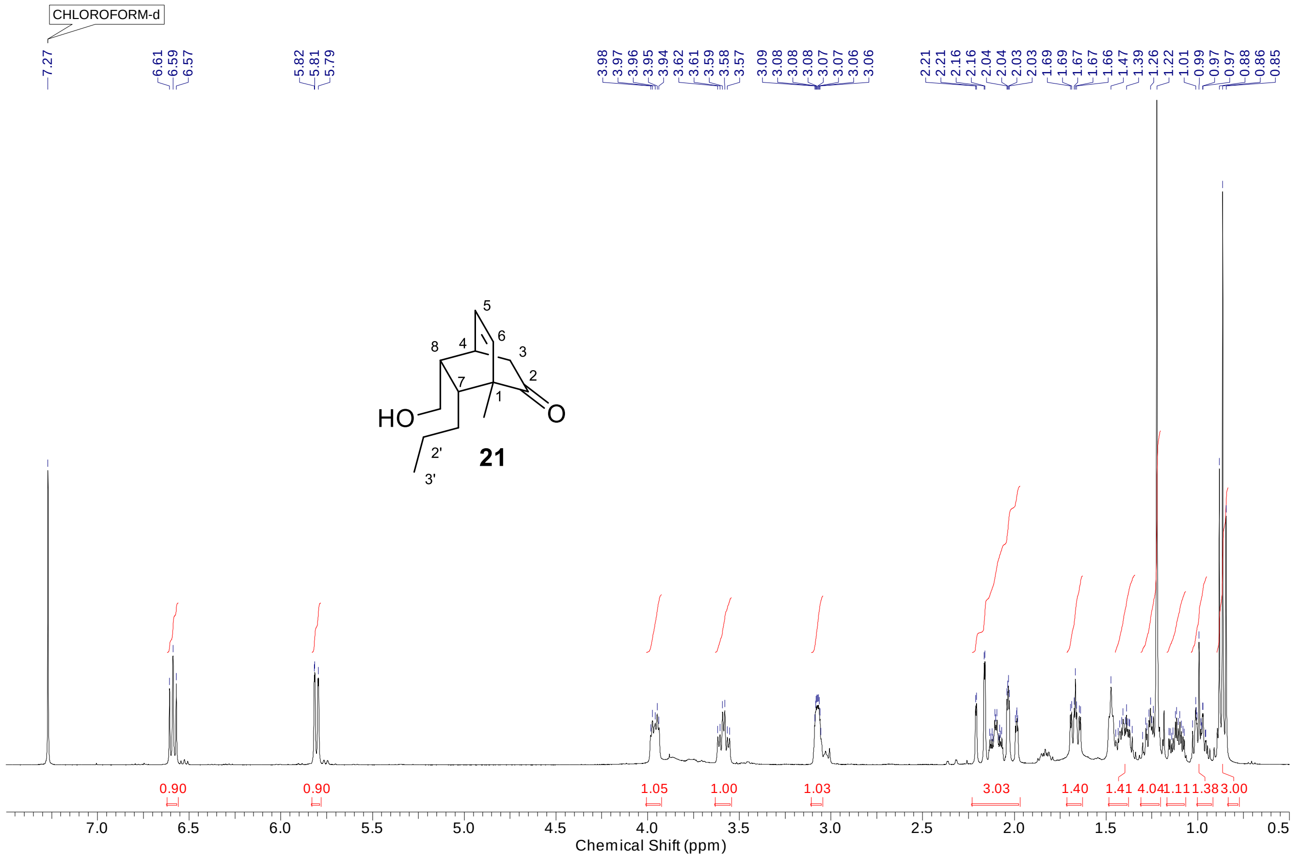

^{1}H NMR (400 MHz) spectrum of hydroxymethylketone **21** in $CDCl_3$ (0.5 – 7.5 ppm)

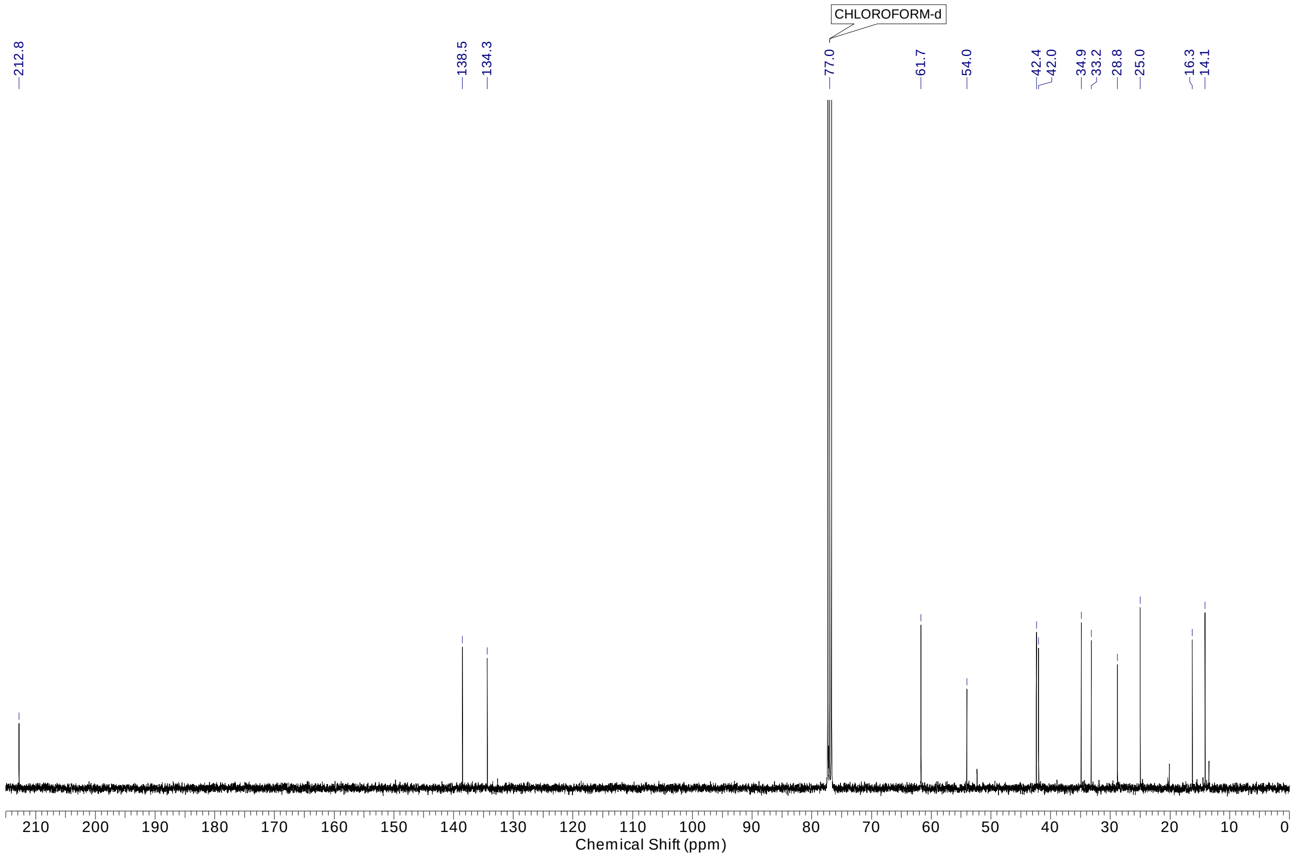

^{13}C NMR (100 MHz) spectrum of hydroxymethylketone **21** in $CDCl_3$ (0 – 215 ppm)

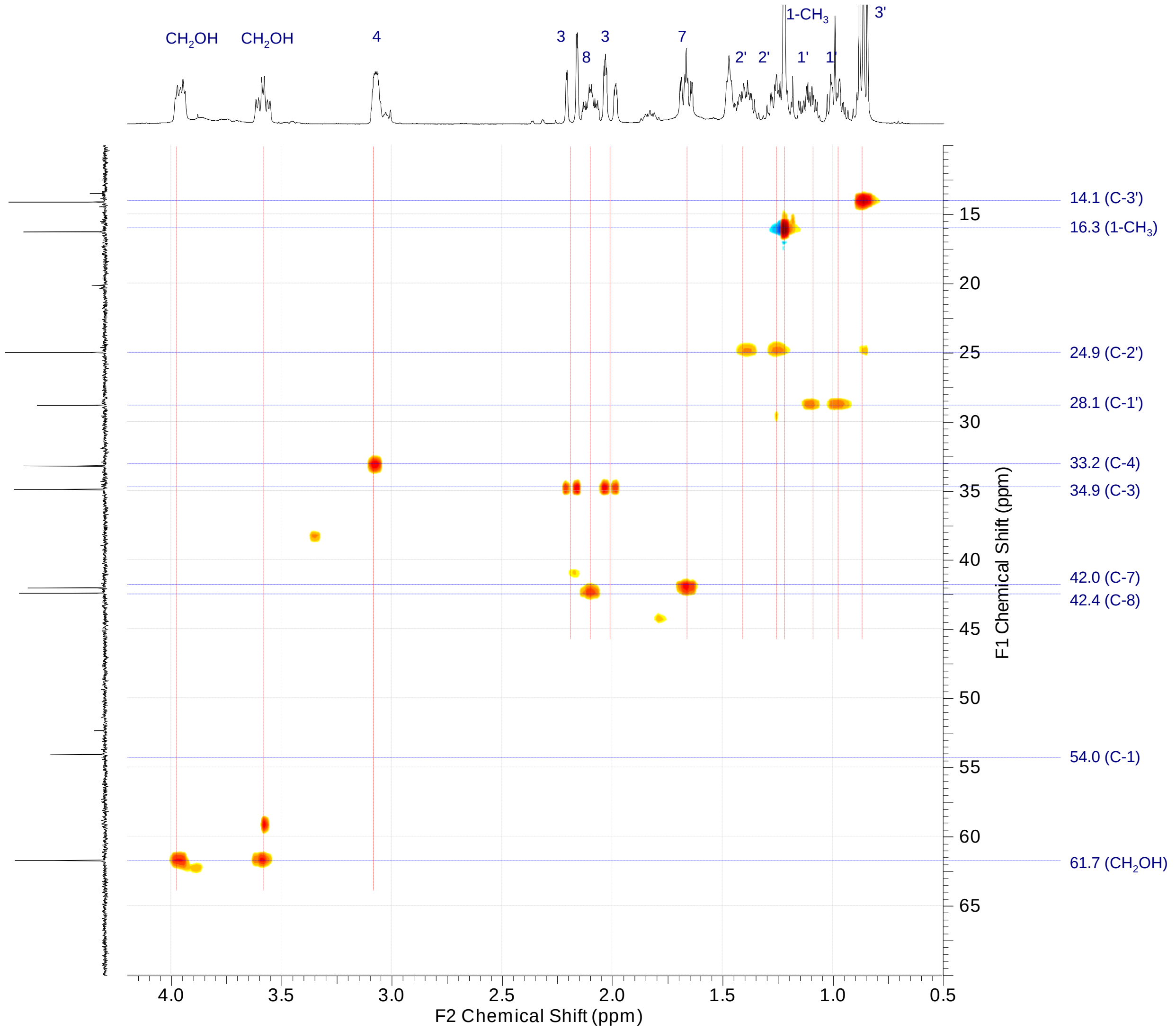

HSQC spectrum of hydroxymethylketone **21** in $CDCl_3$ (0.5 – 4.2, 10 – 70 ppm)

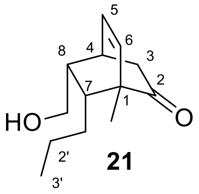

21

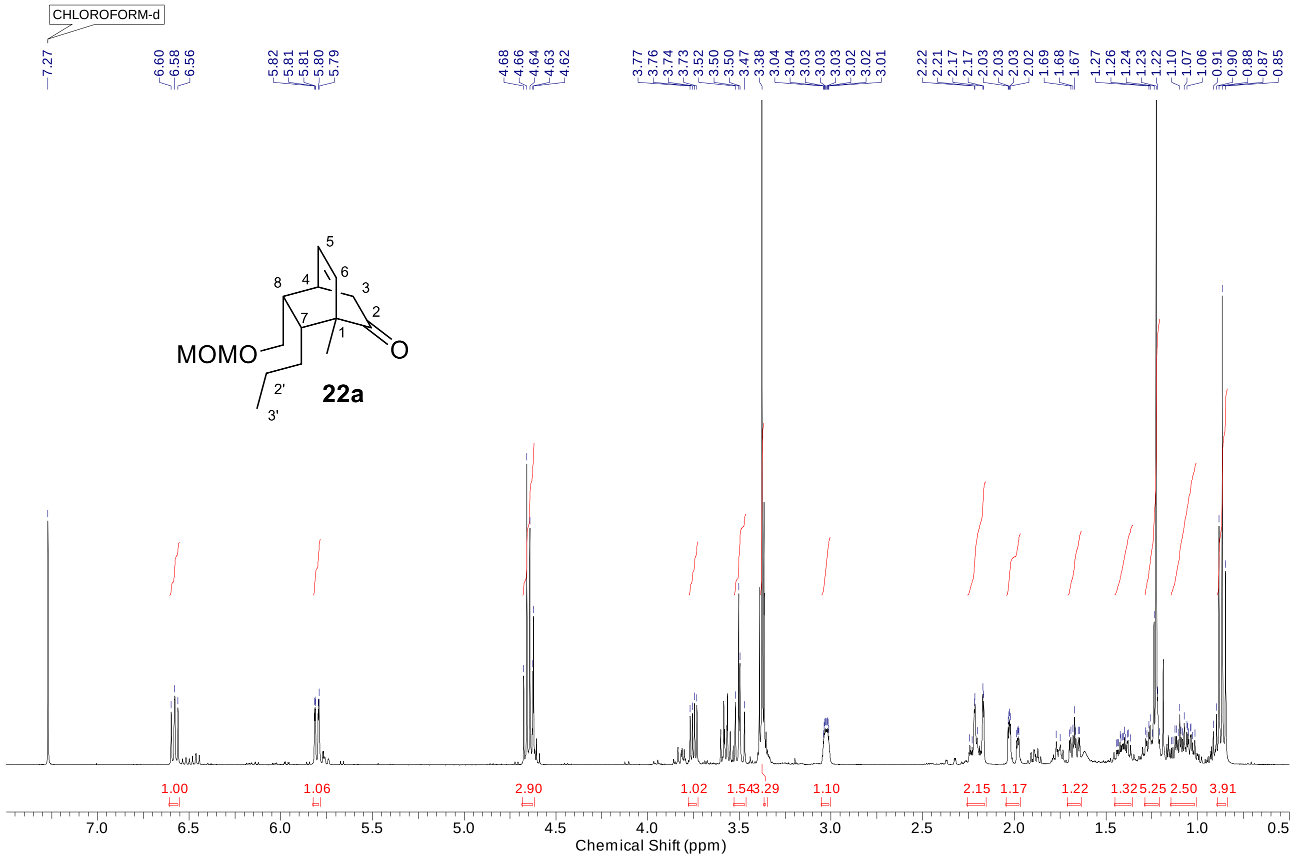

^{1}H NMR (400 MHz) spectrum of bicyclo[2.2.2]octenone **22a** in $CDCl_3$ (0.5 – 7.5 ppm)

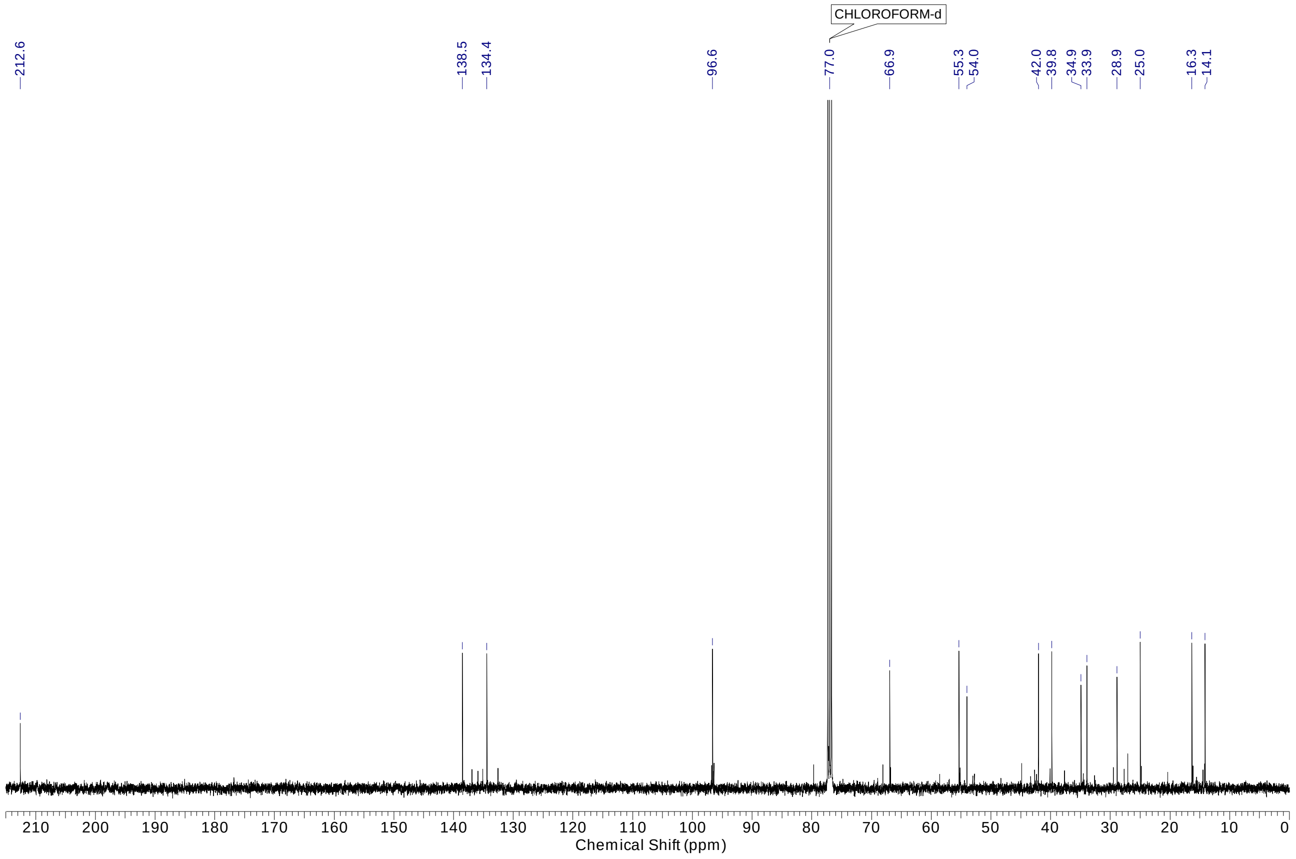

^{13}C NMR (100 MHz) spectrum of bicyclo[2.2.2]octenone **22a** in $CDCl_3$ (0 – 215 ppm)

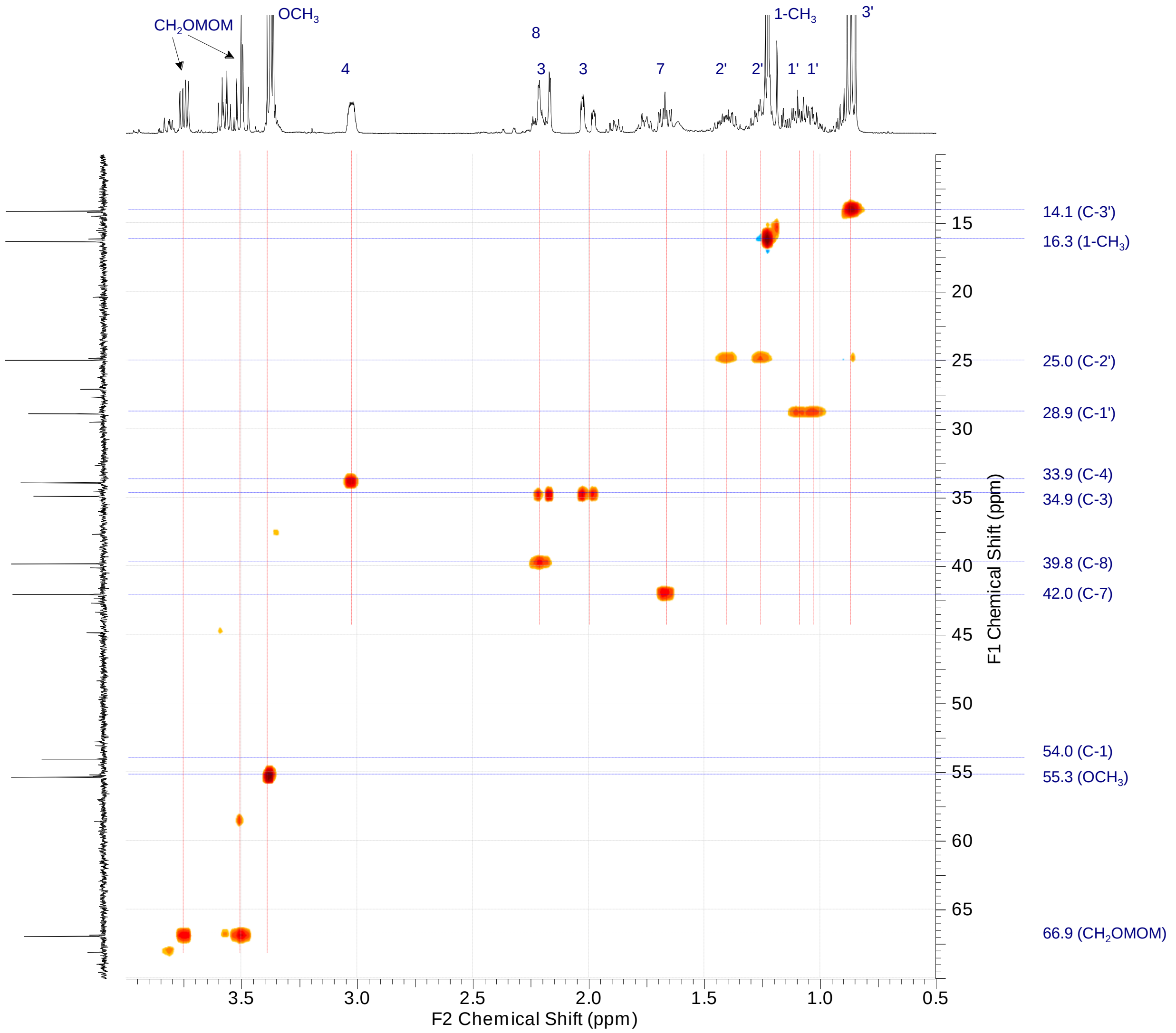

HSQC spectrum of bicyclo[2.2.2]octenone **22a** in $CDCl_3$ (0.5 – 4.0, 10 – 70 ppm)

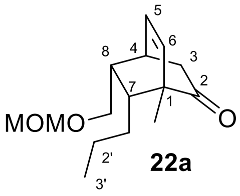

22a

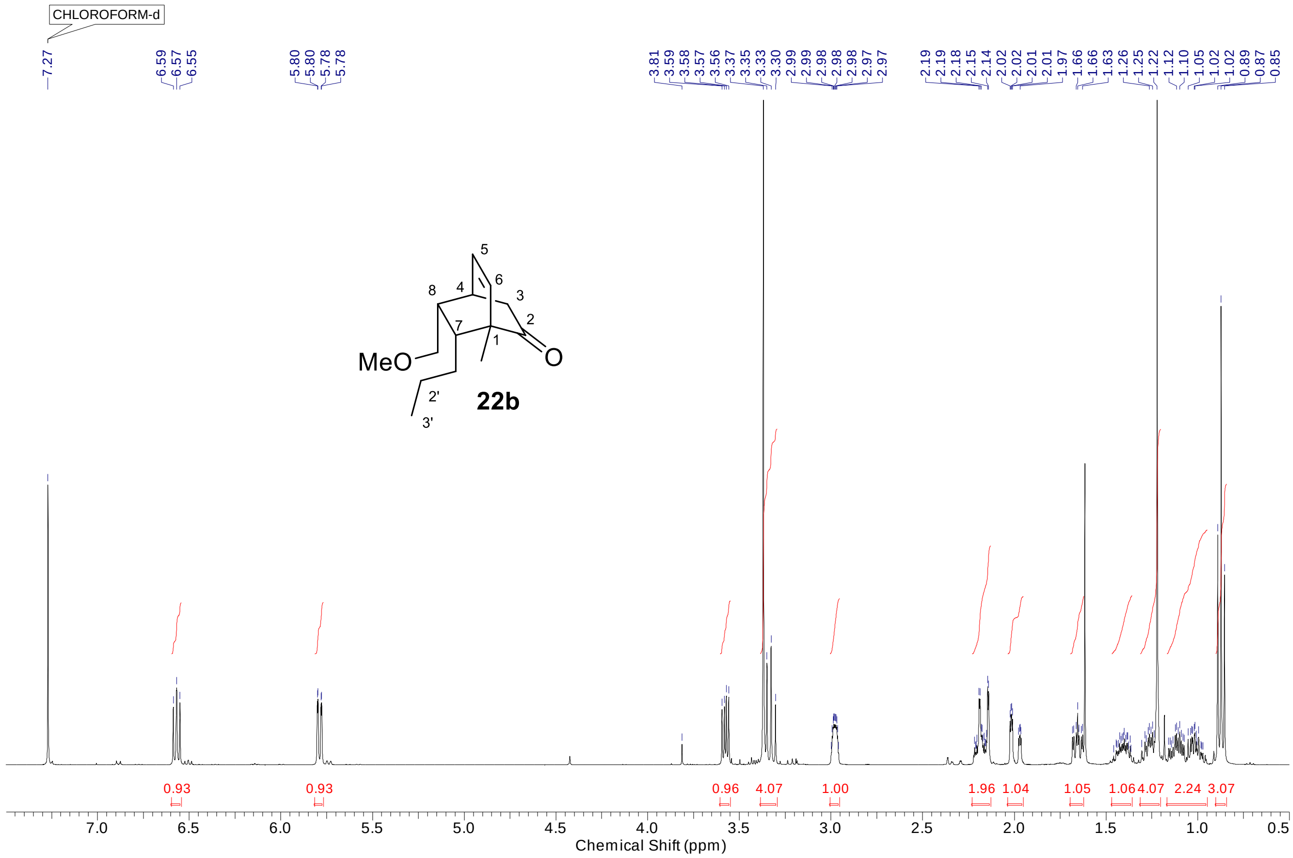

^{1}H NMR (400 MHz) spectrum of bicyclo[2.2.2]octenone **22b** in $CDCl_3$ (0.5 – 7.5 ppm)

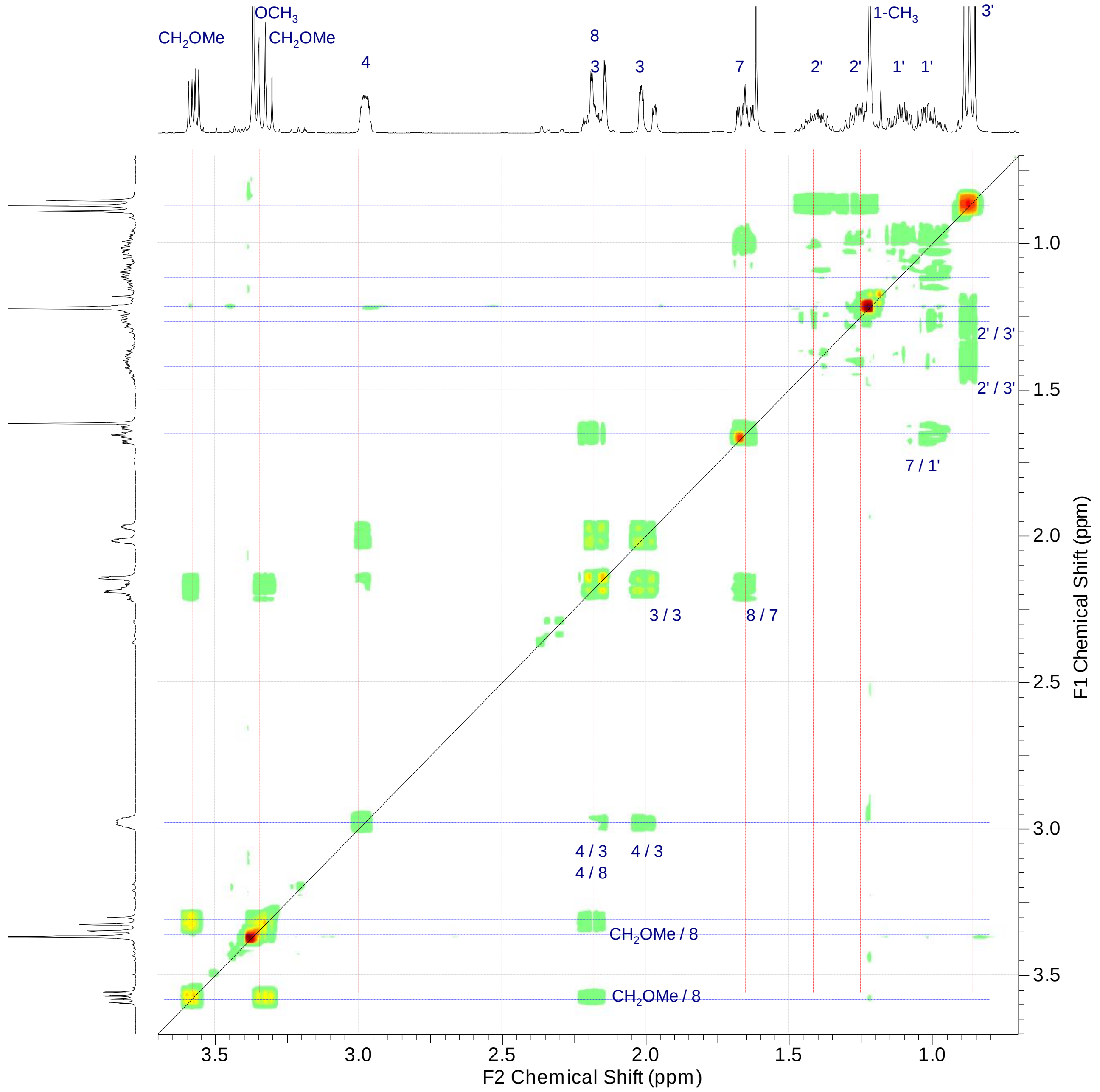

COSY spectrum of bicyclo[2.2.2]octenone **22b** in $CDCl_3$ (0.7 – 3.7 ppm)

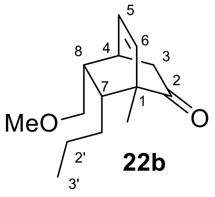

22b

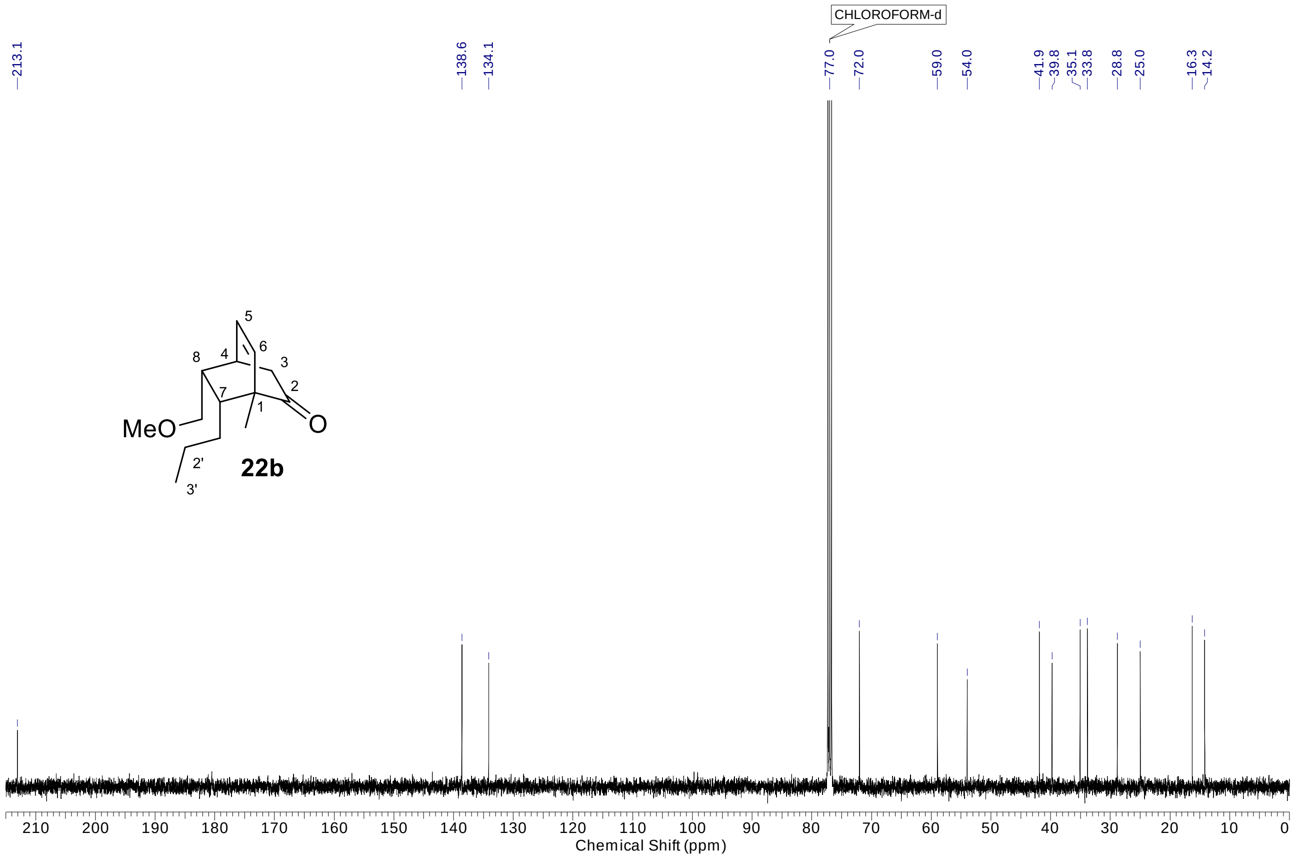

^{13}C NMR (100 MHz) spectrum of bicyclo[2.2.2]octenone **22b** in $CDCl_3$ (0 – 215 ppm)

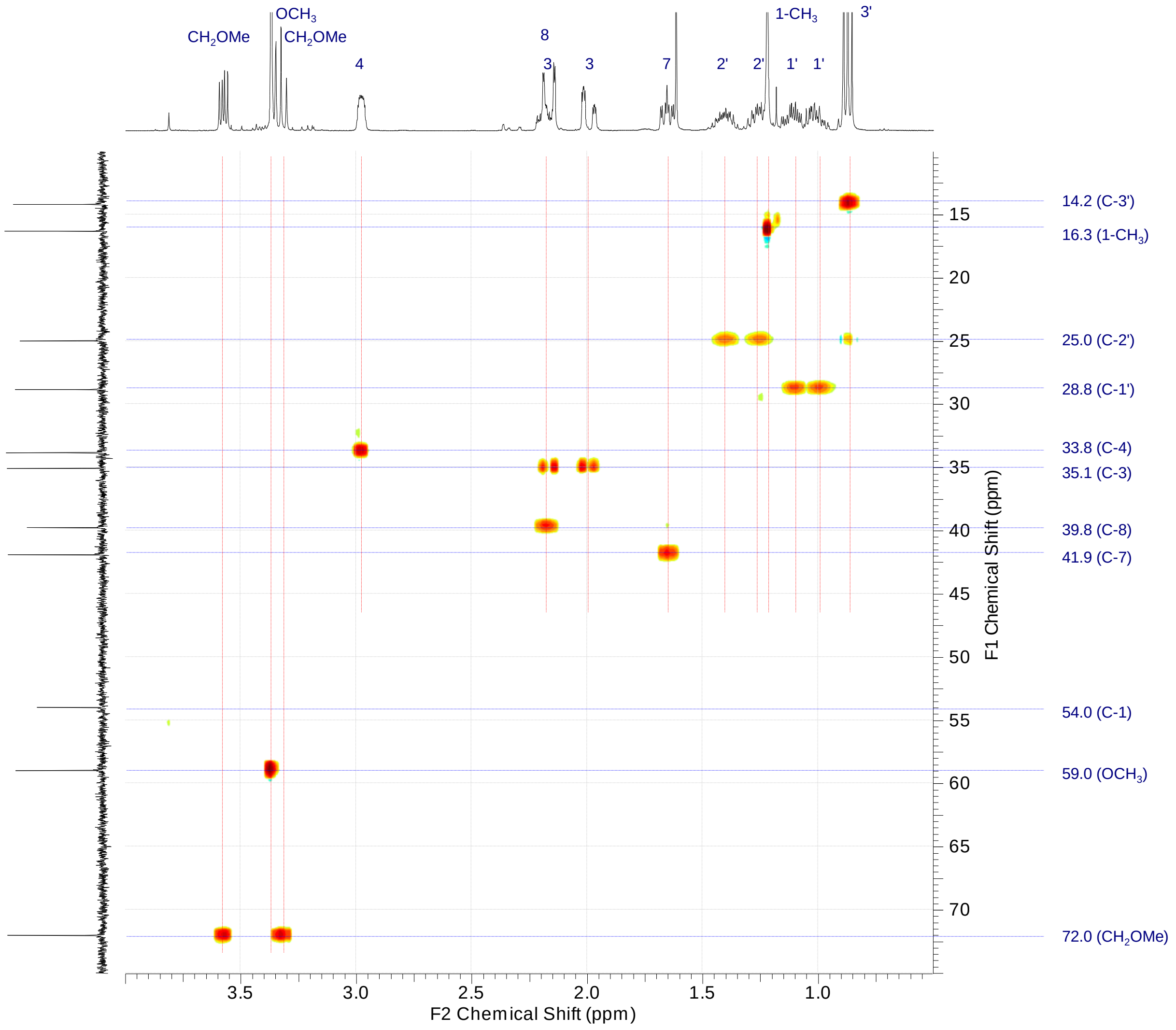

HSQC spectrum of bicyclo[2.2.2]octenone **22b** in $CDCl_3$ (0.5 – 4.0, 10 – 75 ppm)

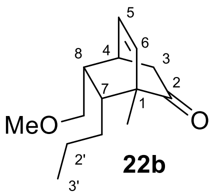

22b

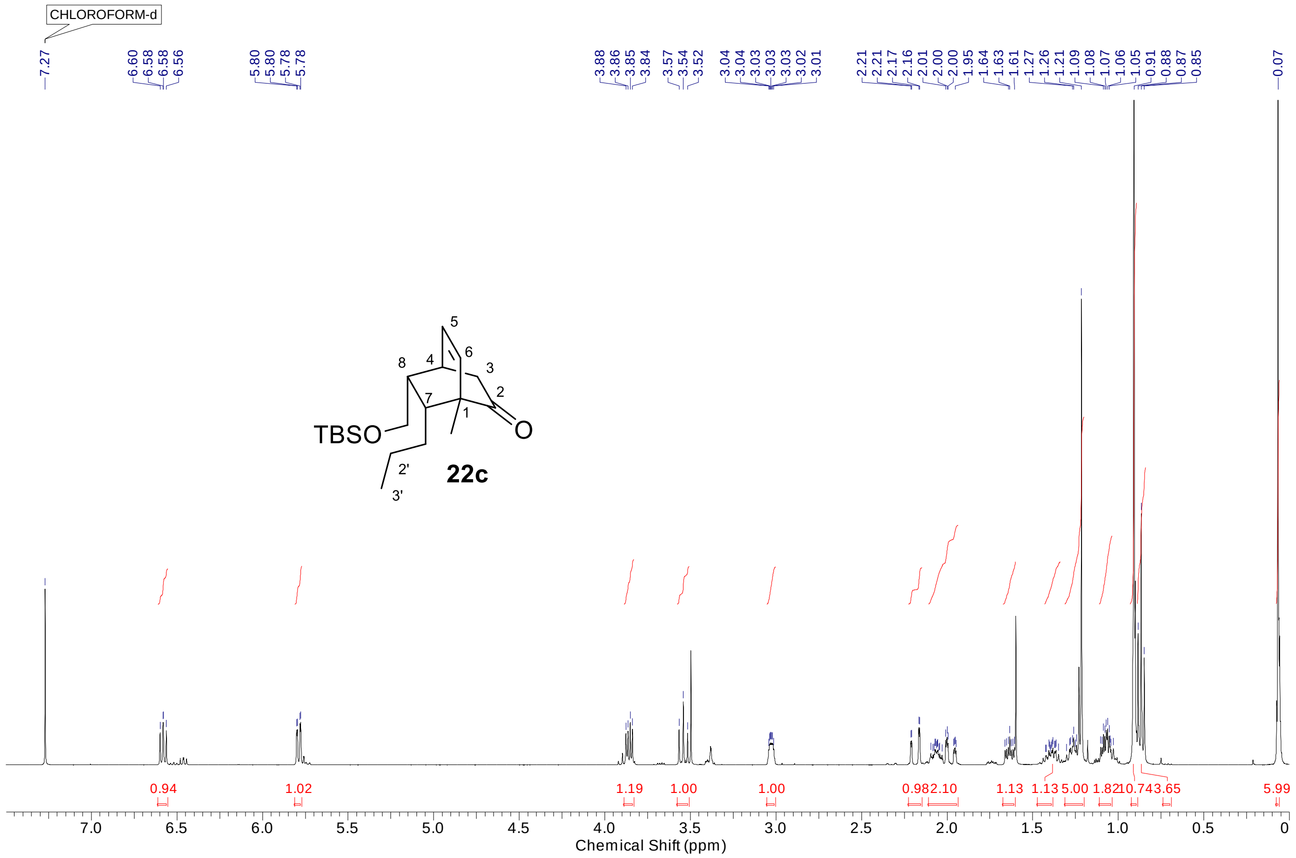

^{1}H NMR (400 MHz) spectrum of bicyclo[2.2.2]octenone **22c** in $CDCl_3$ (0.0 – 7.5 ppm)

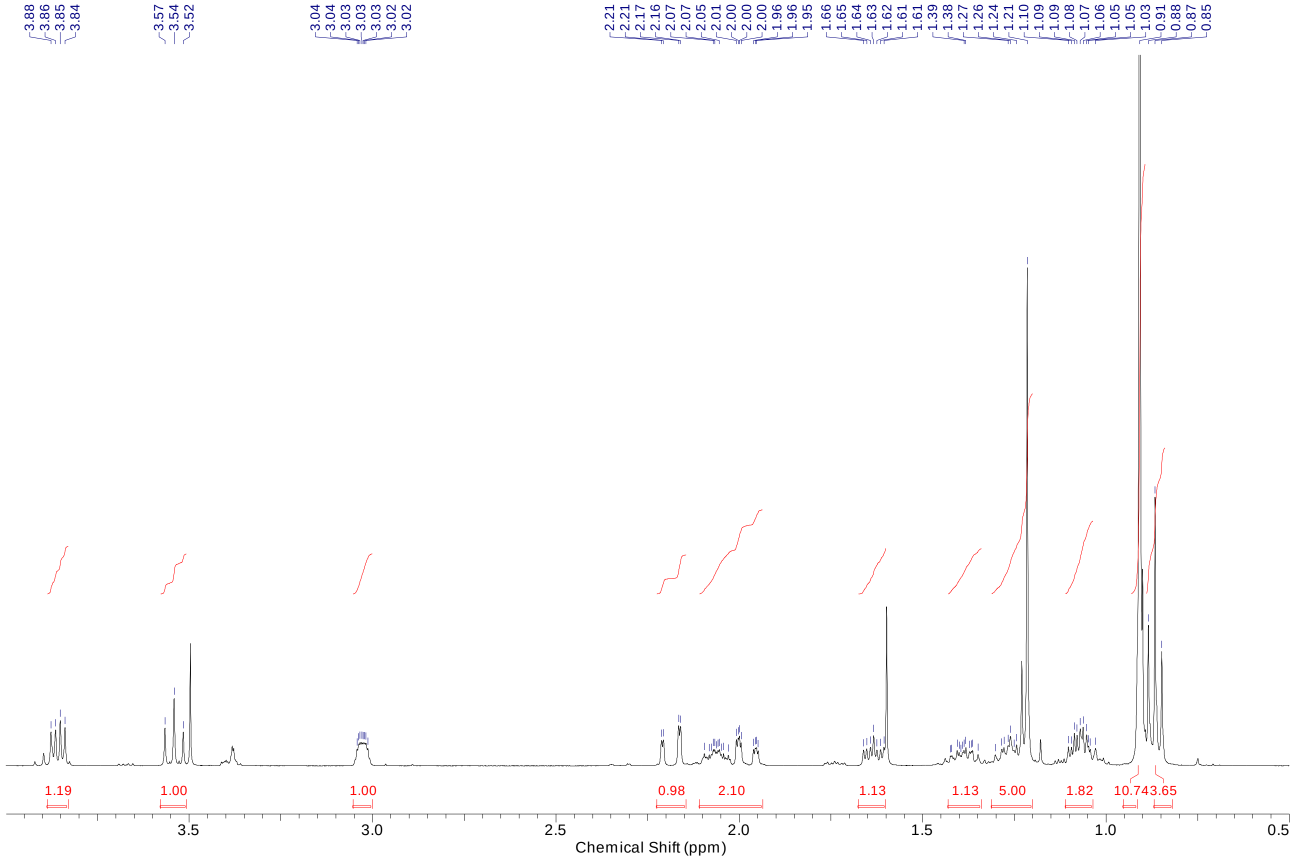

^{1}H NMR (400 MHz) spectrum of bicyclo[2.2.2]octenone **22c** in $CDCl_3$ (0.5 – 4.0 ppm)

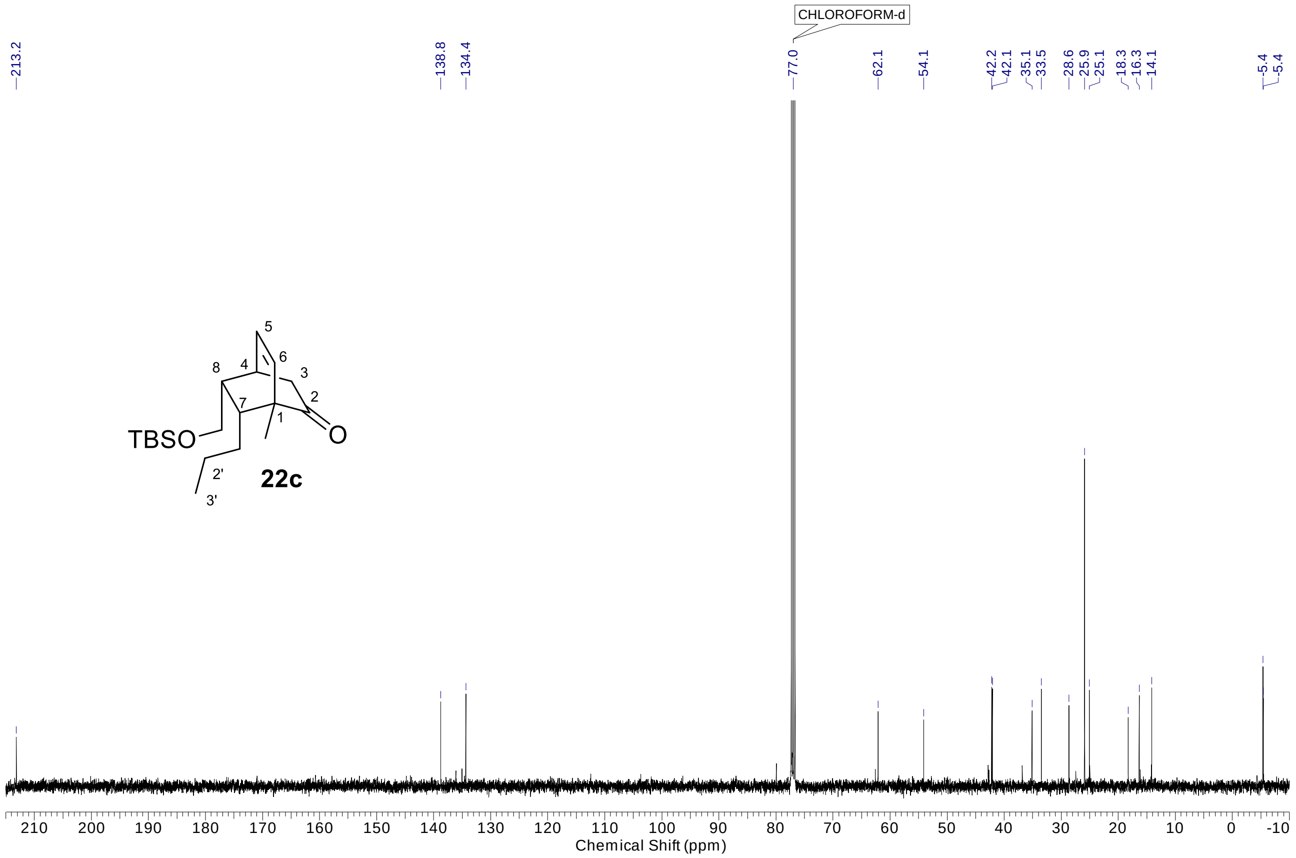

^{13}C NMR (100 MHz) spectrum of bicyclo[2.2.2]octenone **22c** in $CDCl_3$ (–10 – 215 ppm)

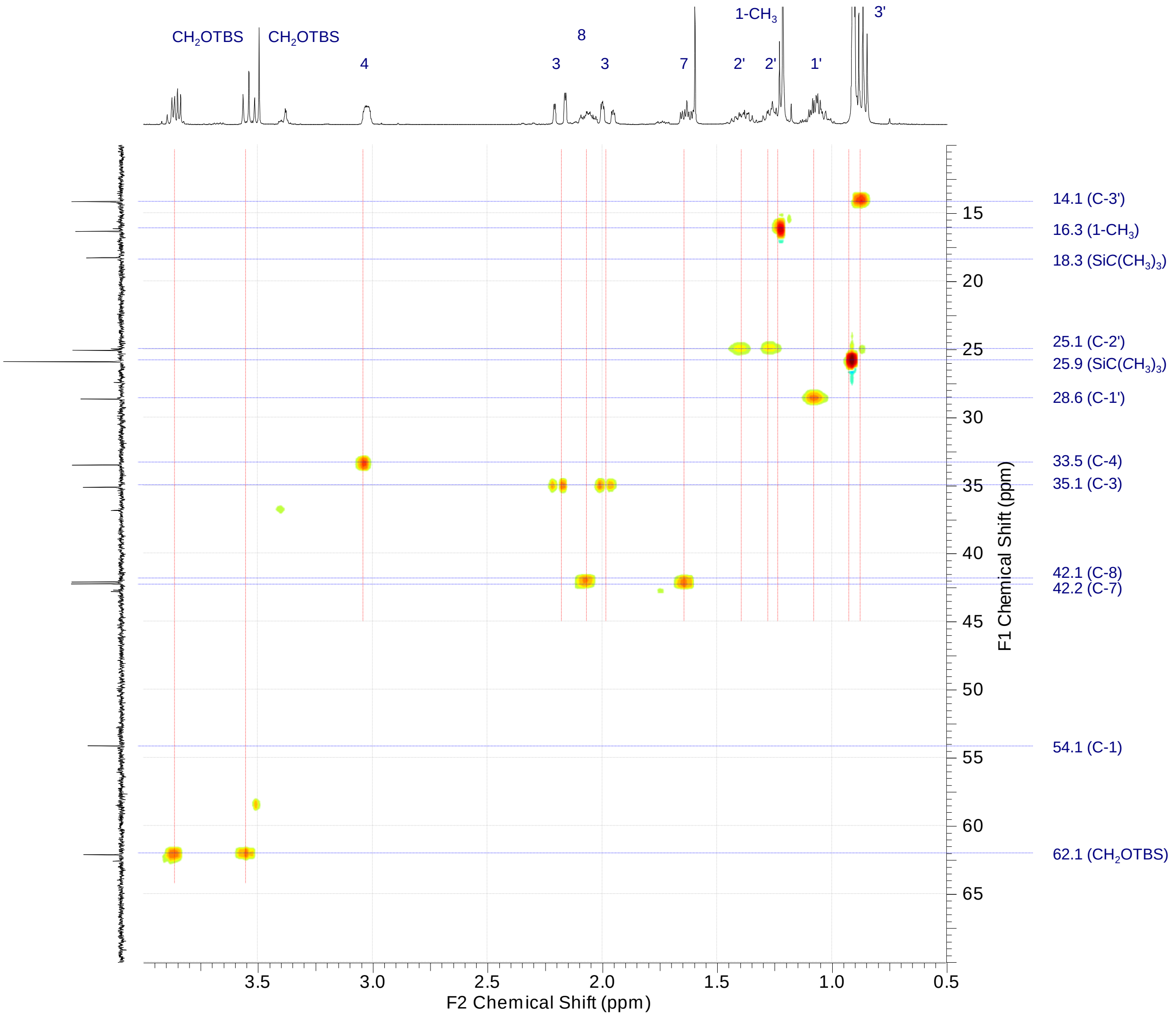

HSQC spectrum of bicyclo[2.2.2]octenone **22c** in $CDCl_3$ (0.5 – 4.0, 10 – 70 ppm)

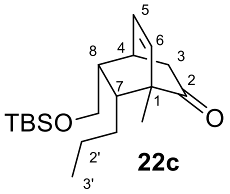

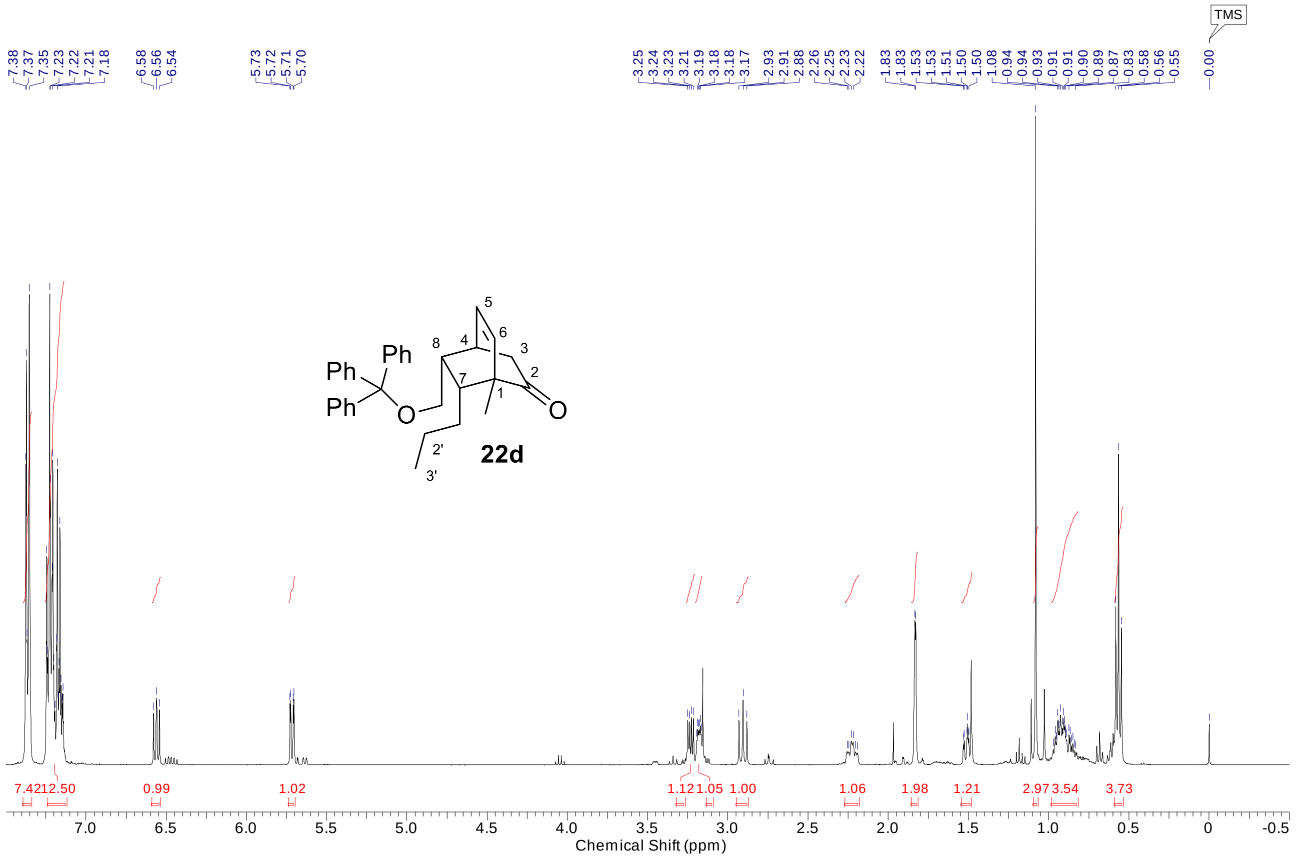

^{1}H NMR (400 MHz) spectrum of bicyclo[2.2.2]octenone **22d** in $CDCl_3$ (–0.5 – 7.5 ppm)

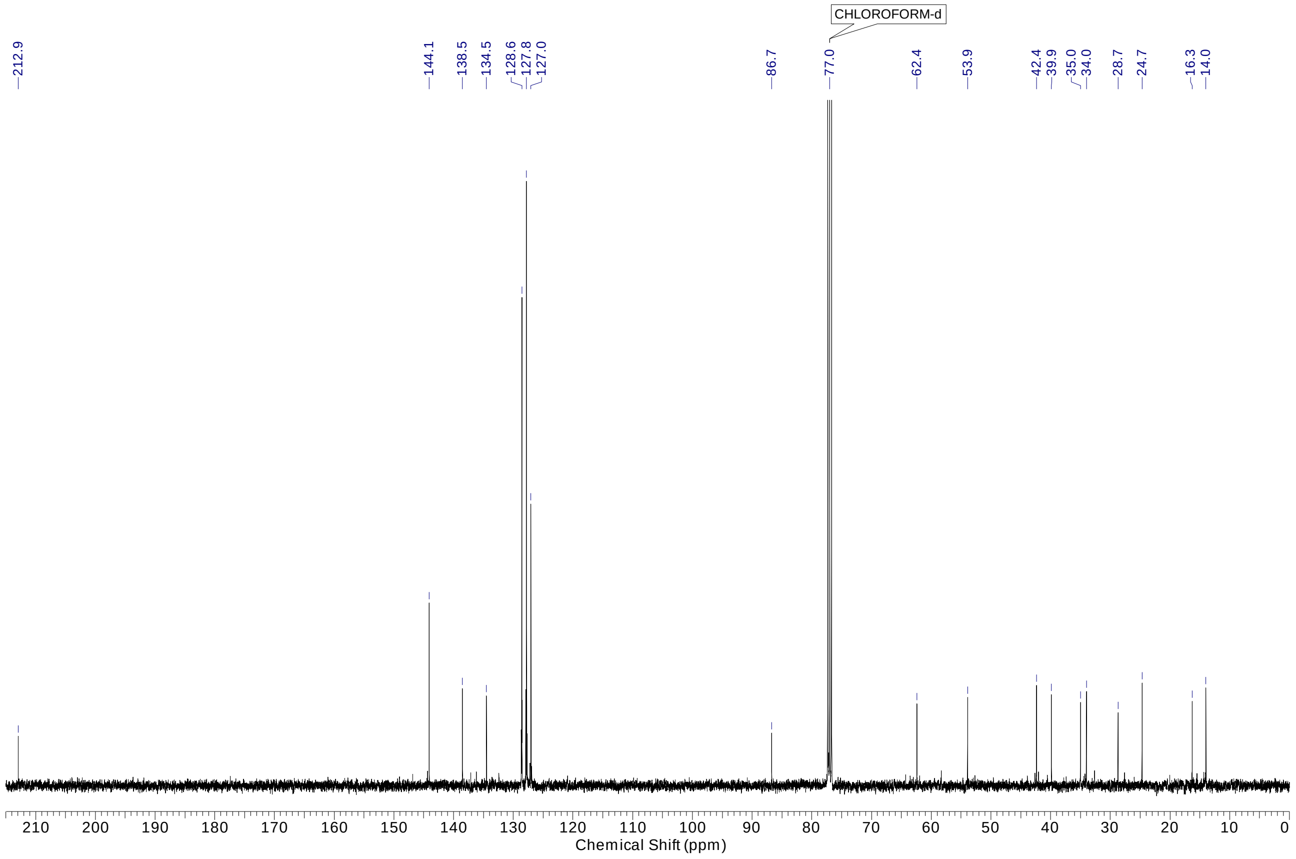

^{13}C NMR (100 MHz) spectrum of bicyclo[2.2.2]octenone **22d** in $CDCl_3$ (0 – 215 ppm)

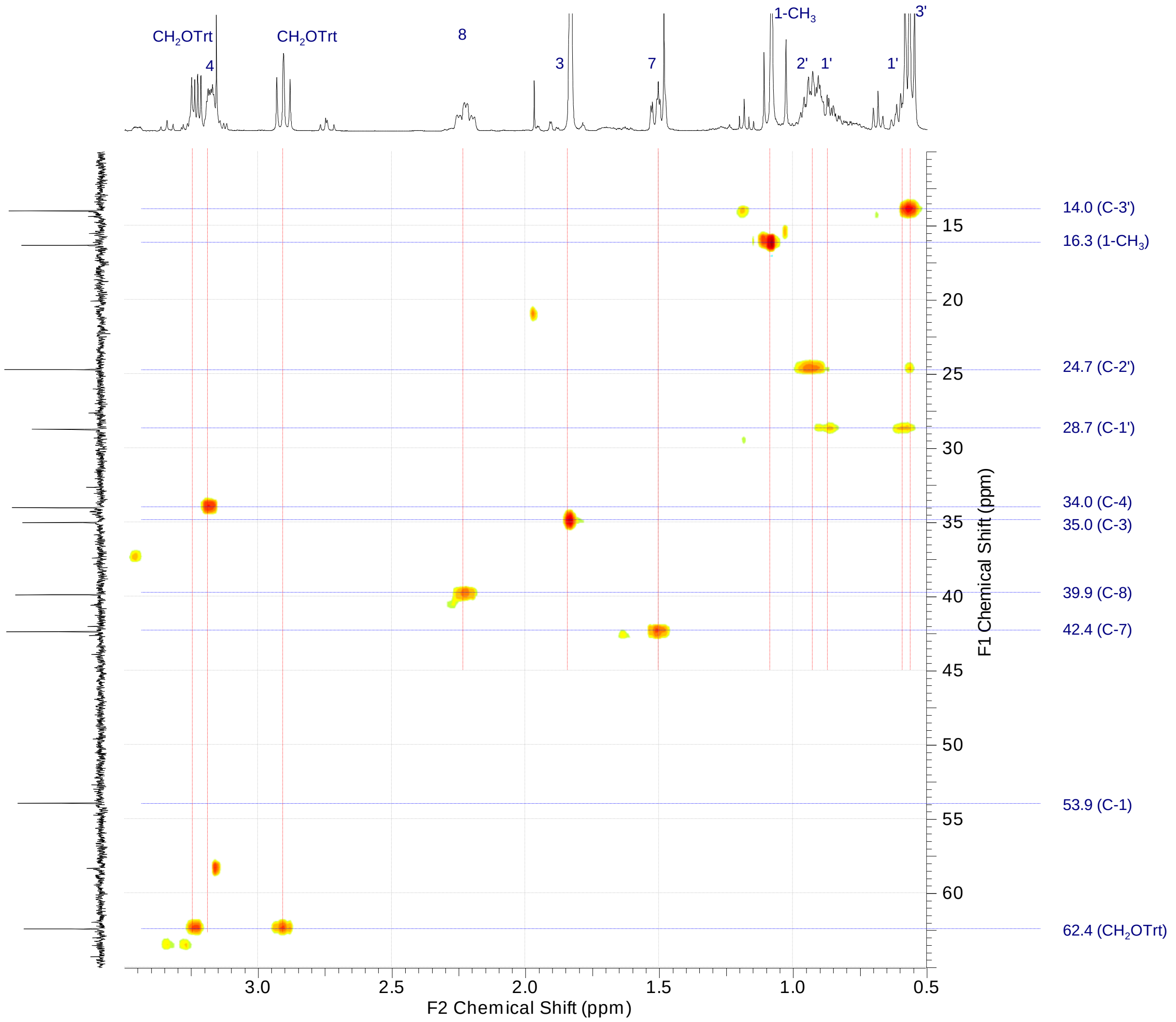

HSQC spectrum of bicyclo[2.2.2]octenone **22d** in $CDCl_3$ (0.5 – 3.5, 10 – 65 ppm)

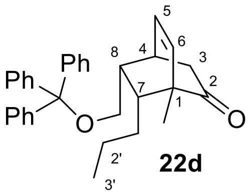

22d

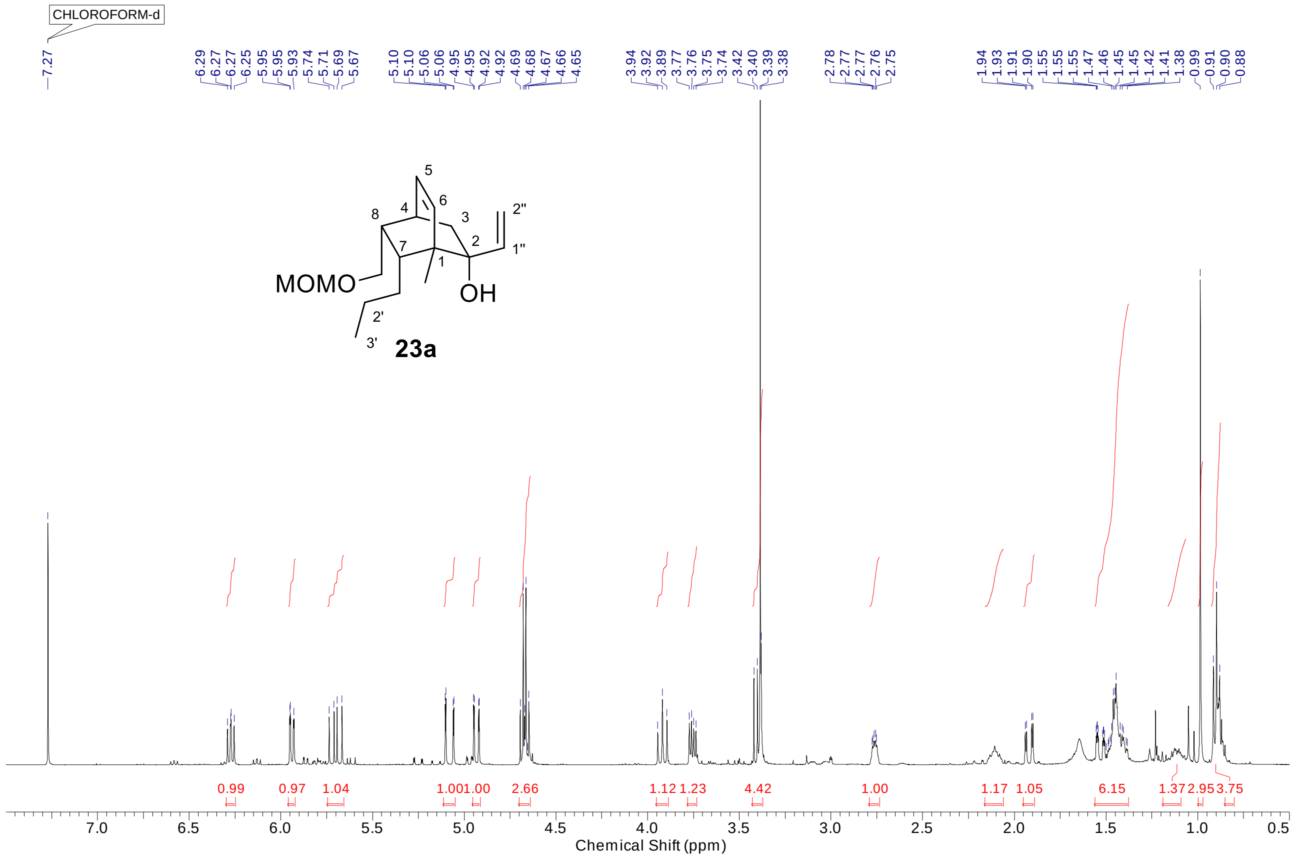

^{1}H NMR (400 MHz) spectrum of bicyclo[2.2.2]octenol **23a** in $CDCl_3$ (0.5 – 7.5 ppm)

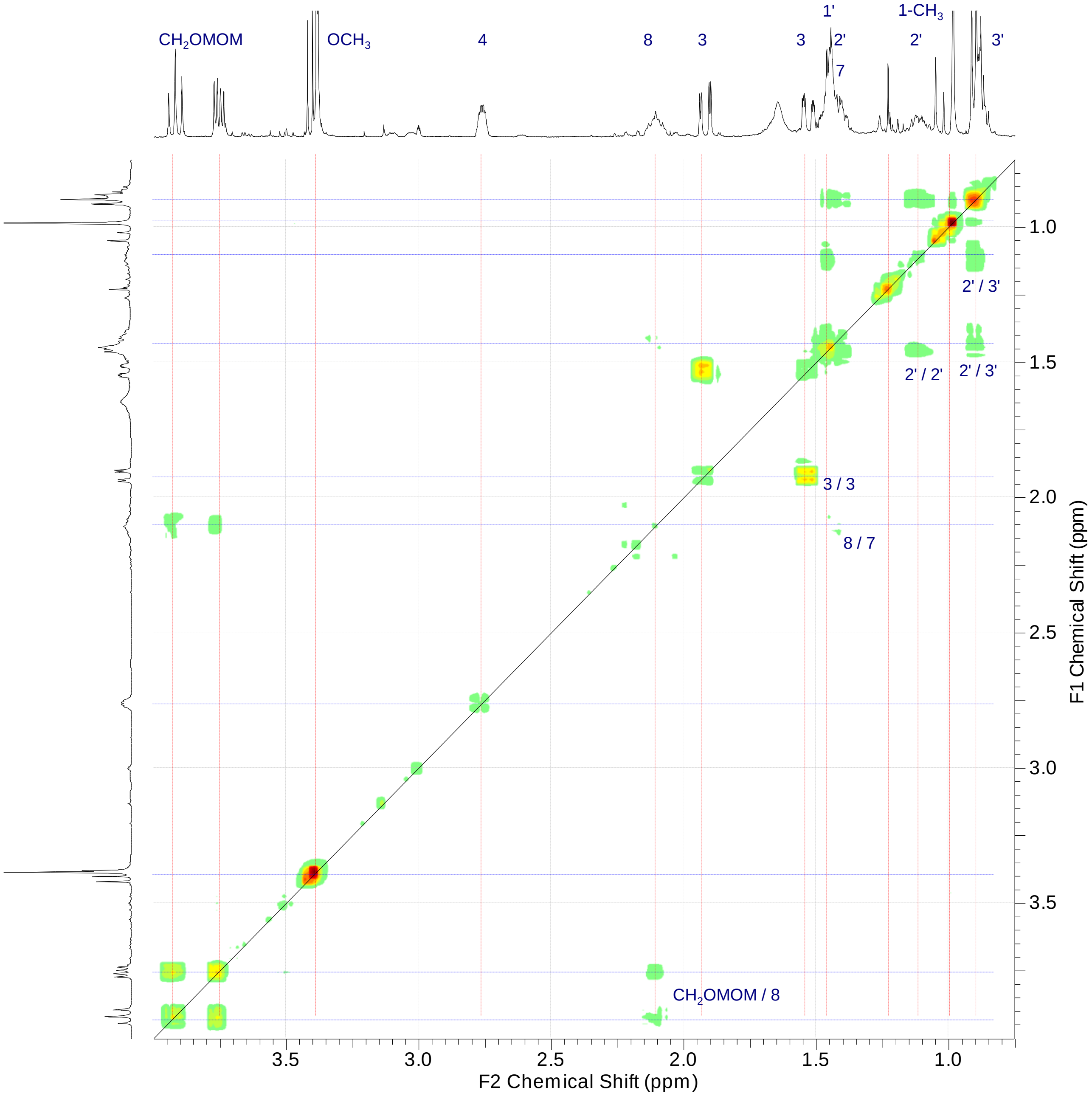

COSY spectrum of bicyclo[2.2.2]octenol **23a** in $CDCl_3$ (0.75 – 4.0 ppm)

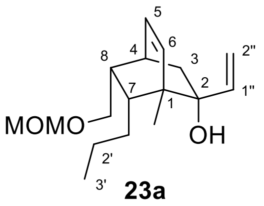

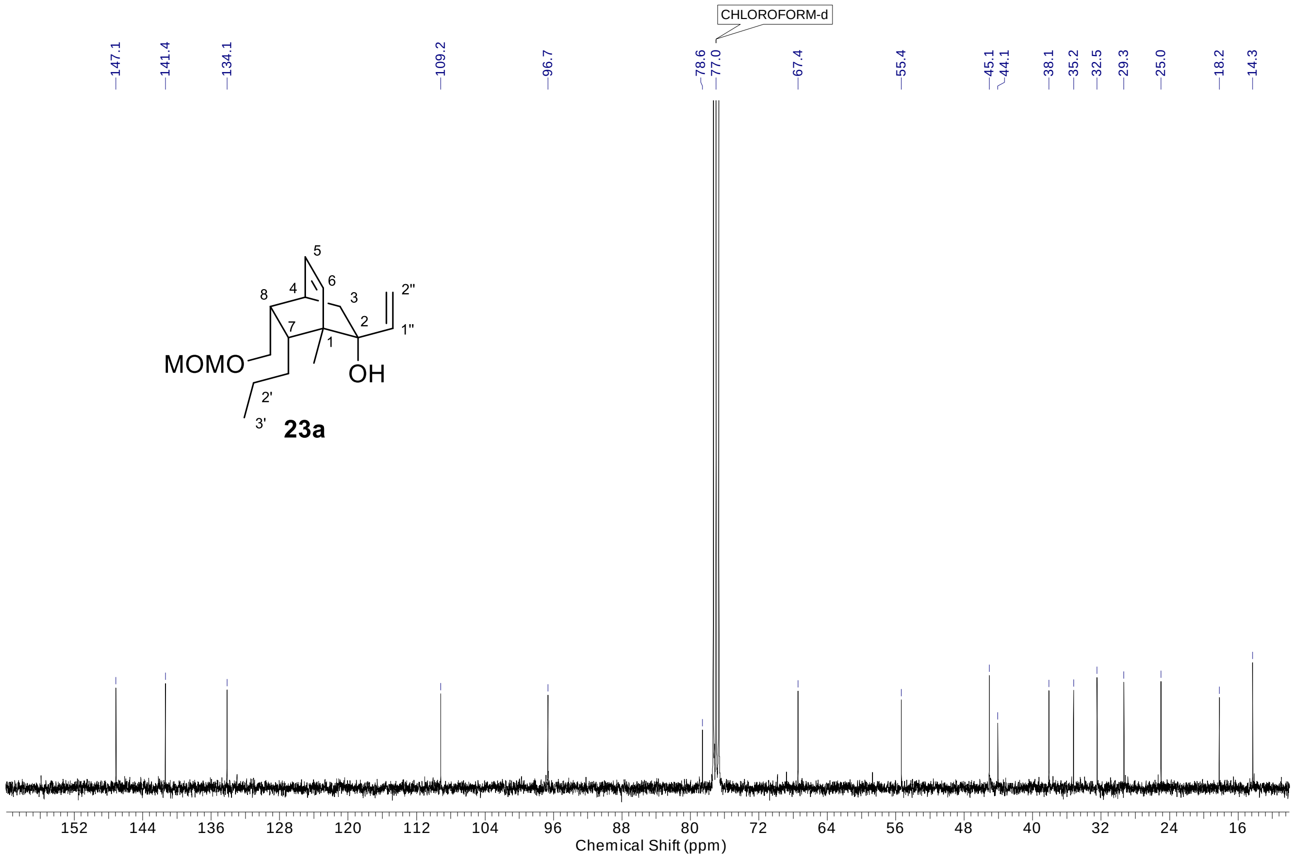

^{13}C NMR (100 MHz) spectrum of bicyclo[2.2.2]octenol **23a** in $CDCl_3$ (10 – 160 ppm)

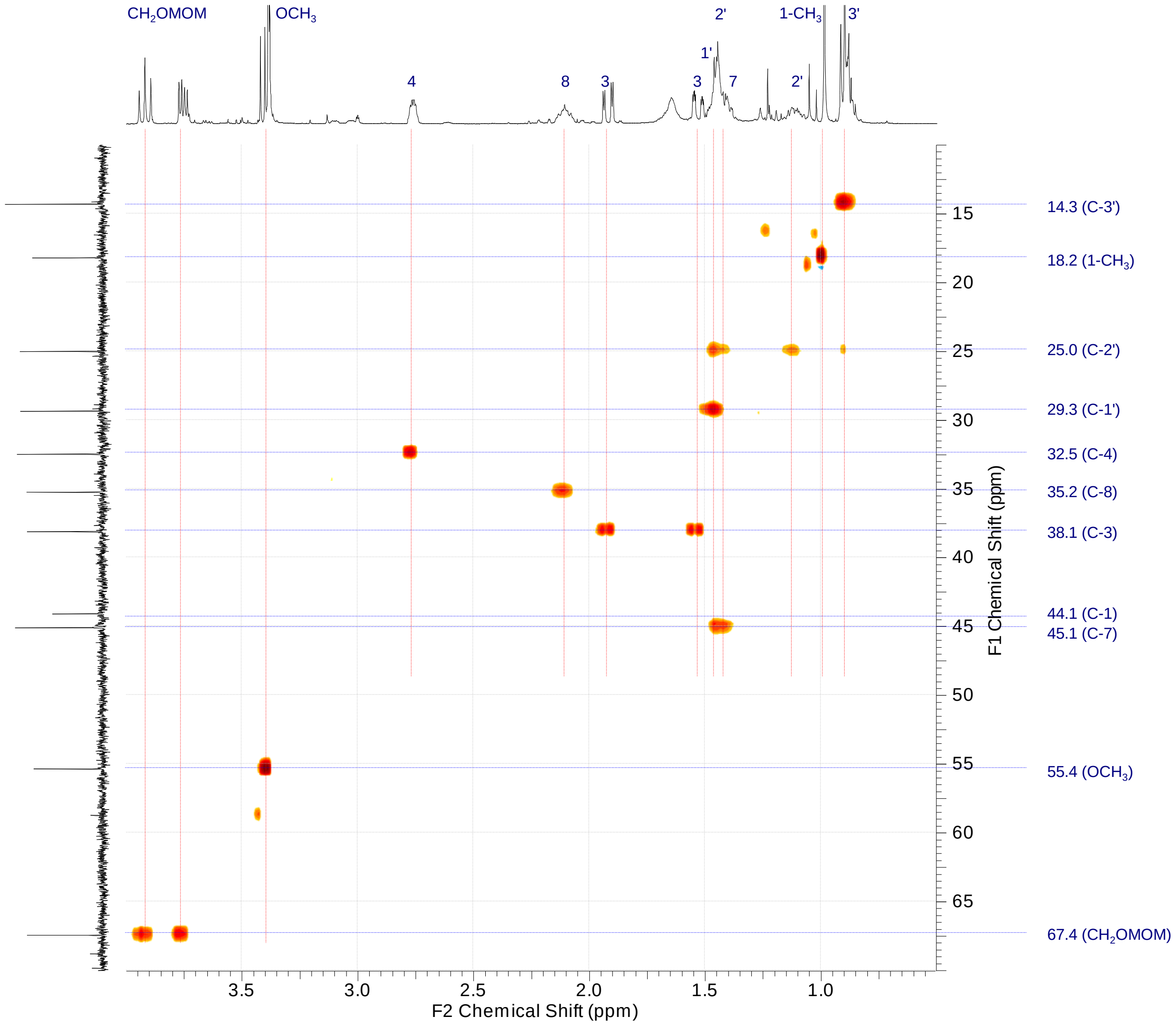

HSQC spectrum of bicyclo[2.2.2]octenonol **23a** in $CDCl_3$ (0.5 – 4.0, 10 – 70 ppm)

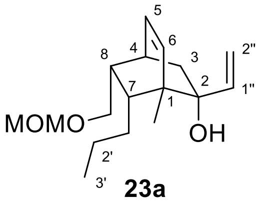

23a

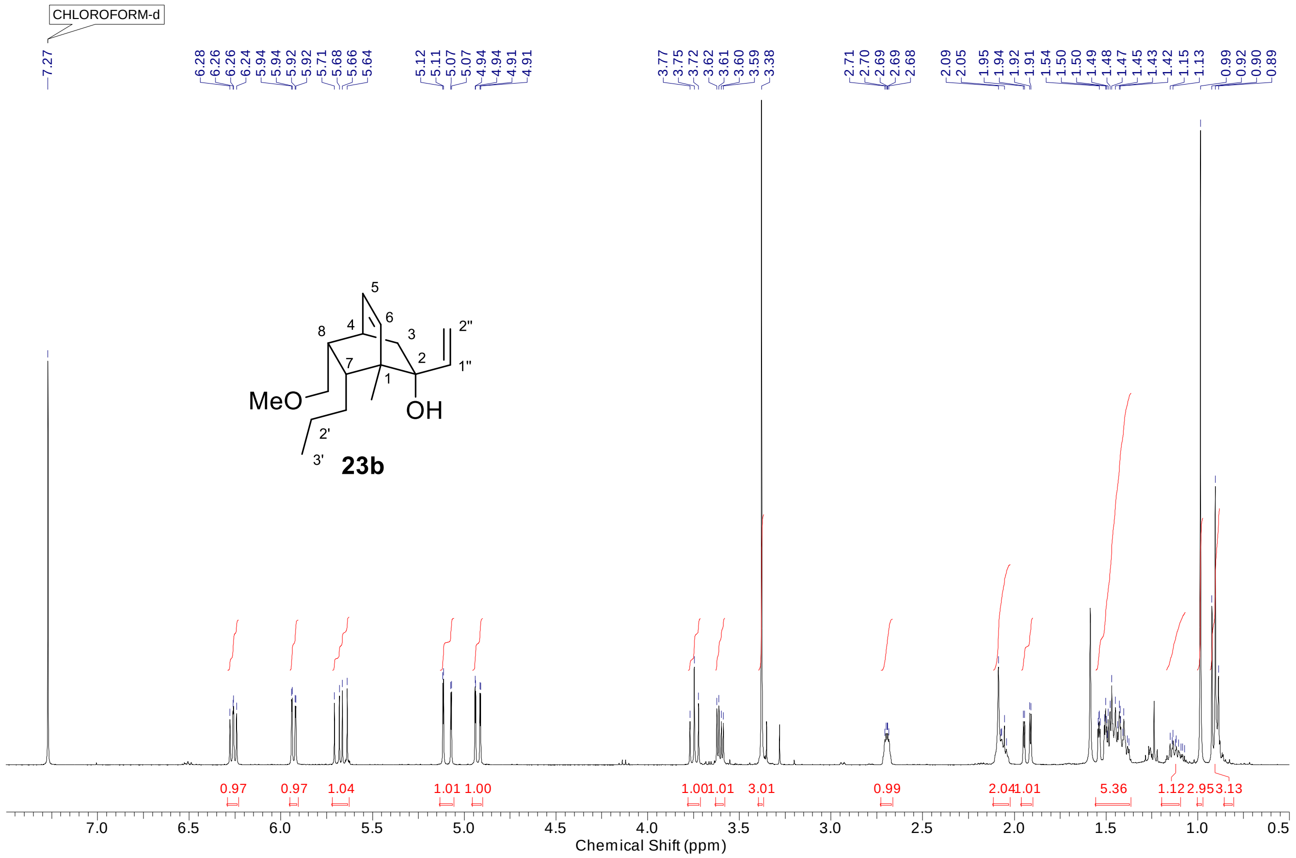

^{1}H NMR (400 MHz) spectrum of bicyclo[2.2.2]octenol **23b** in $CDCl_3$ (0.5 – 7.5 ppm)

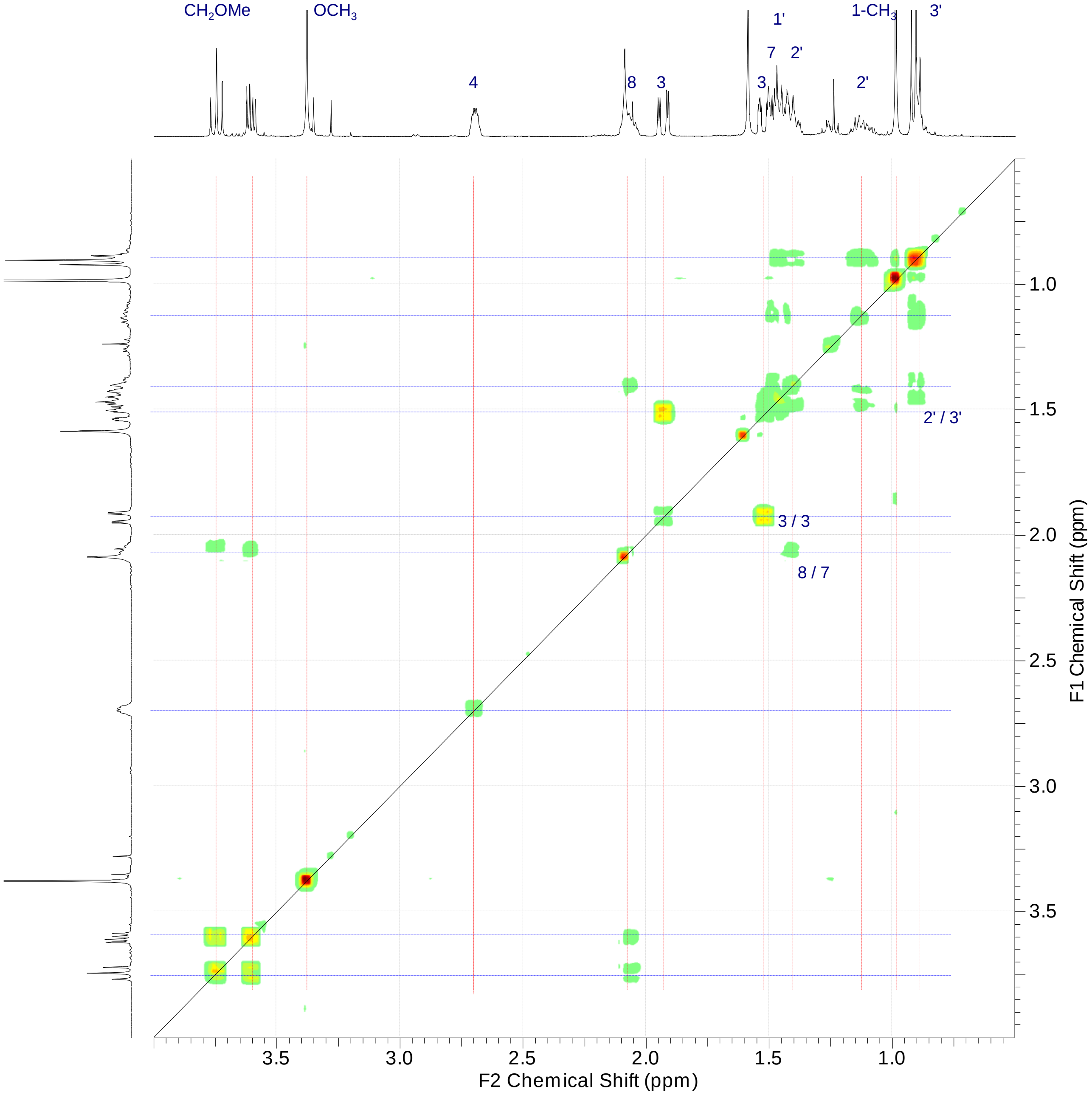

COSY spectrum of bicyclo[2.2.2]octenol **23b** in $CDCl_3$ (0.5 – 4.0 ppm)

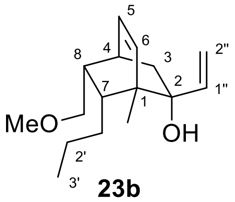

23b

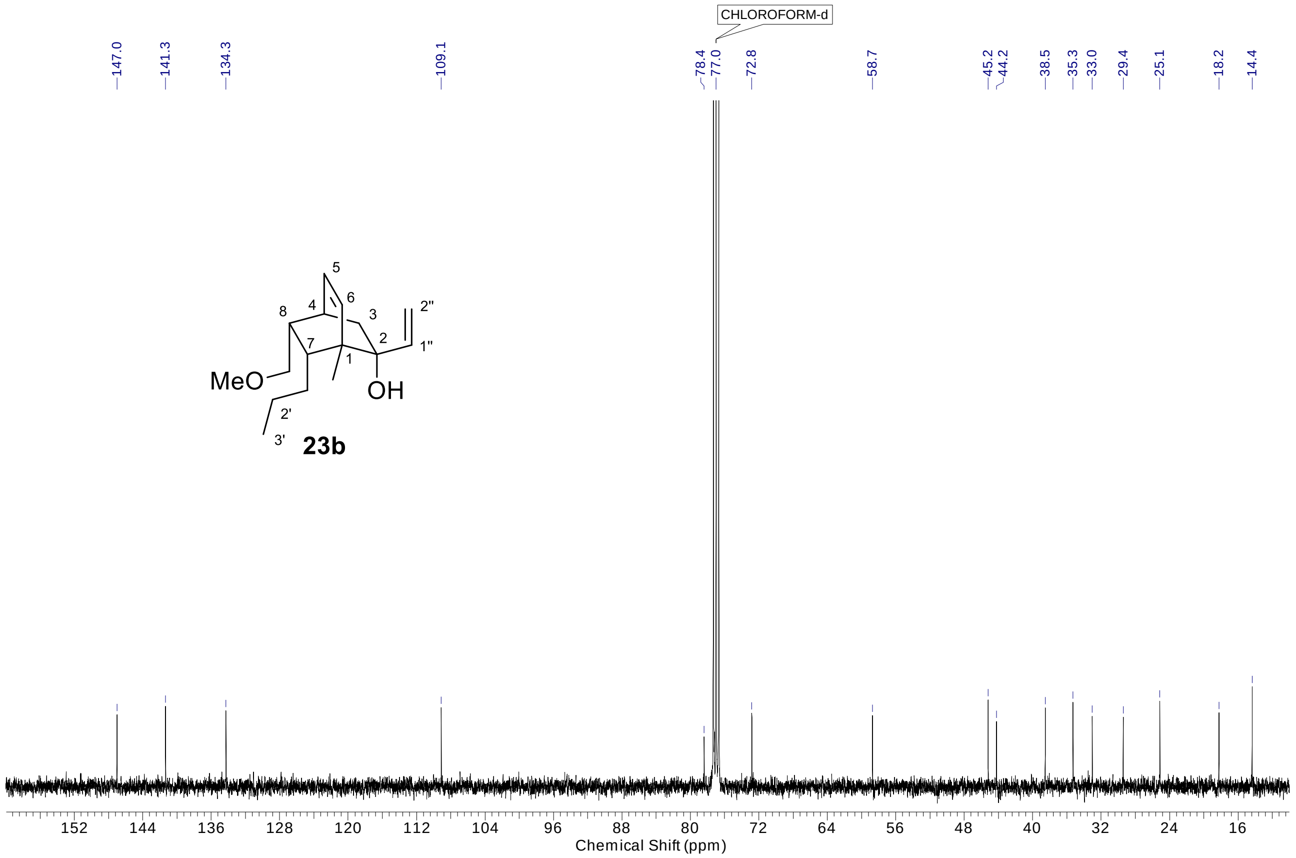

^{13}C NMR (100 MHz) spectrum of bicyclo[2.2.2]octenol **23b** in $CDCl_3$ (10 – 160 ppm)

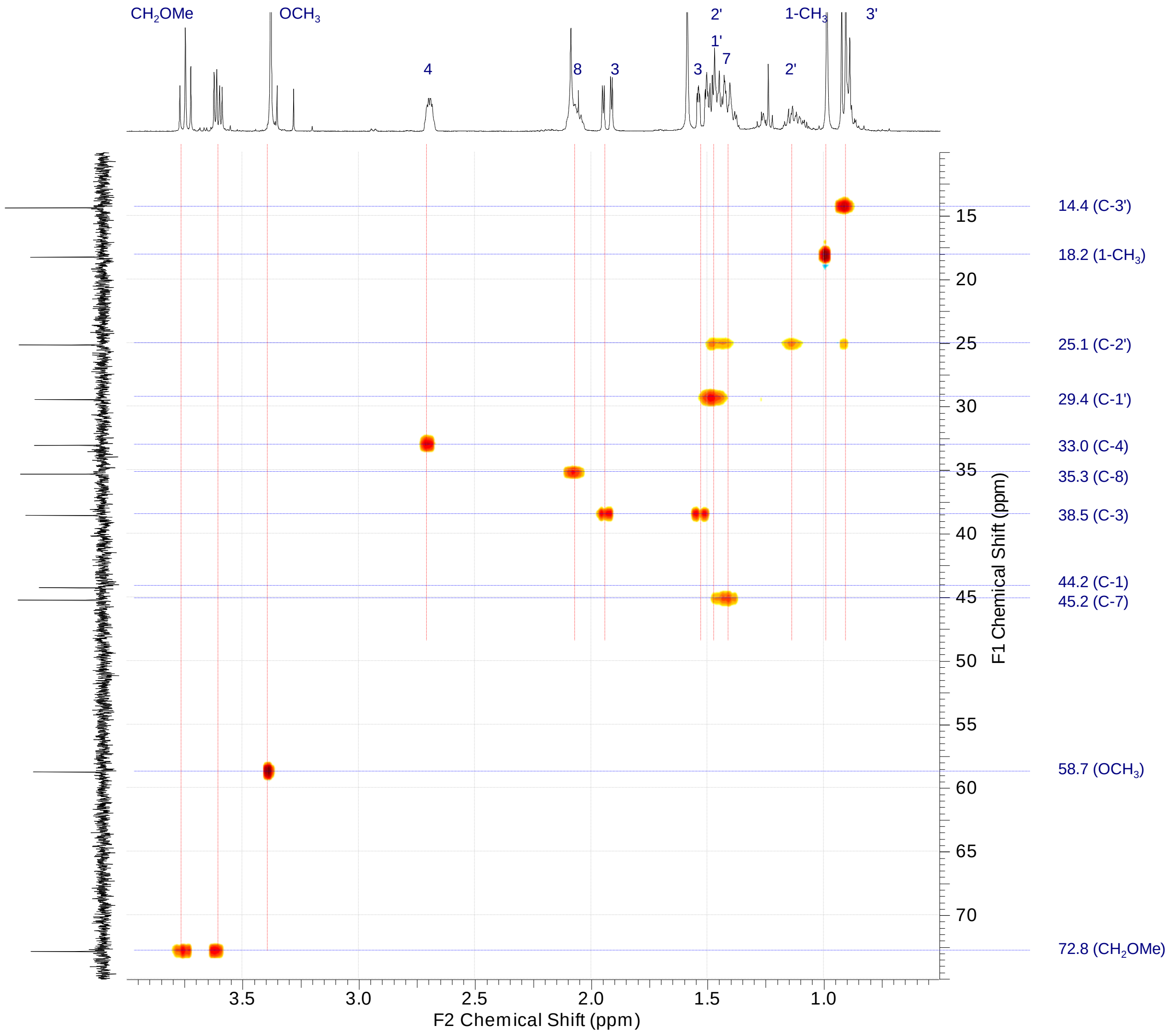

HSQC spectrum of bicyclo[2.2.2]octenonol **23b** in $CDCl_3$ (0.5 – 4.0, 10 – 75 ppm)

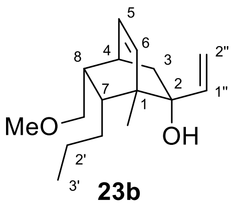

23b

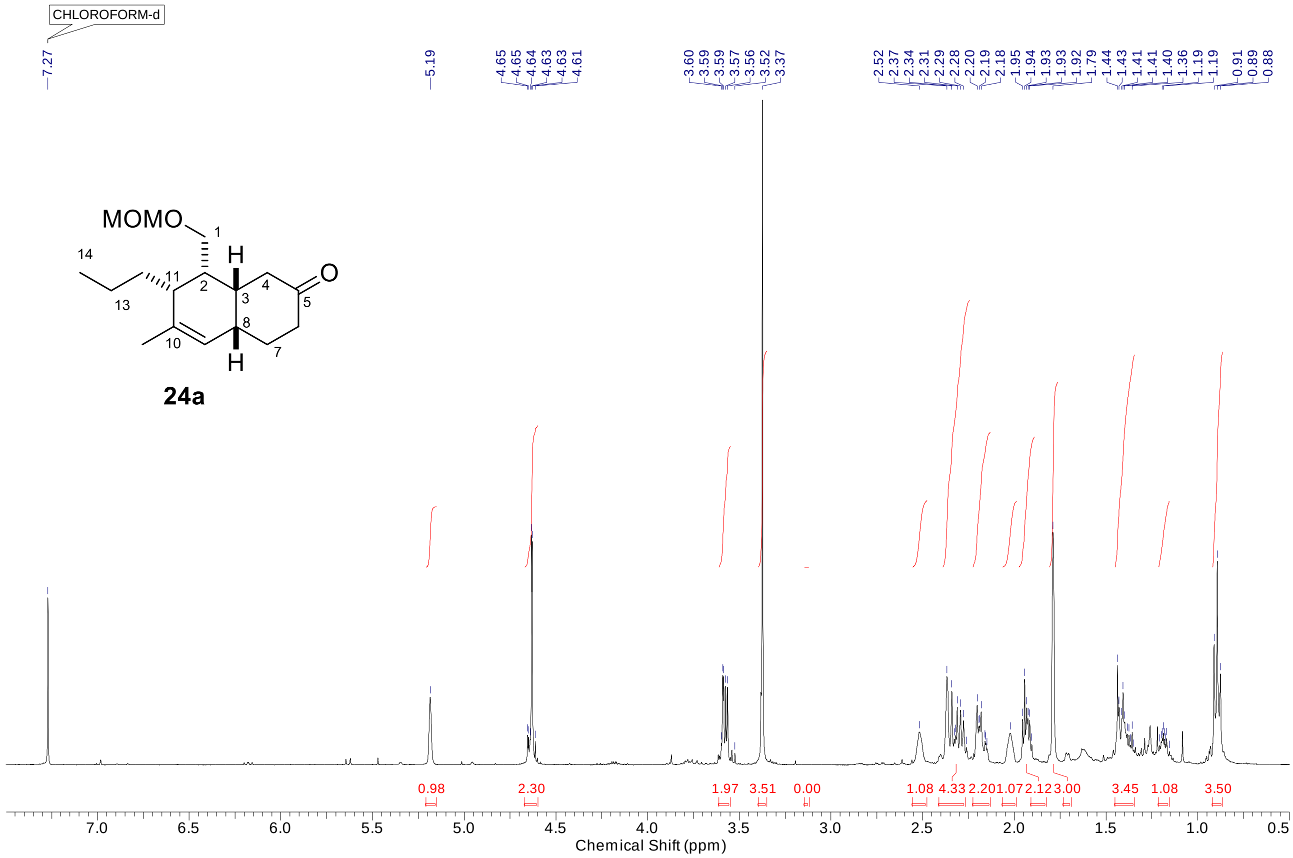

^1H NMR (400 MHz) spectrum of decalinone **24a** in $CDCl_3$ (0.5 – 7.5 ppm)

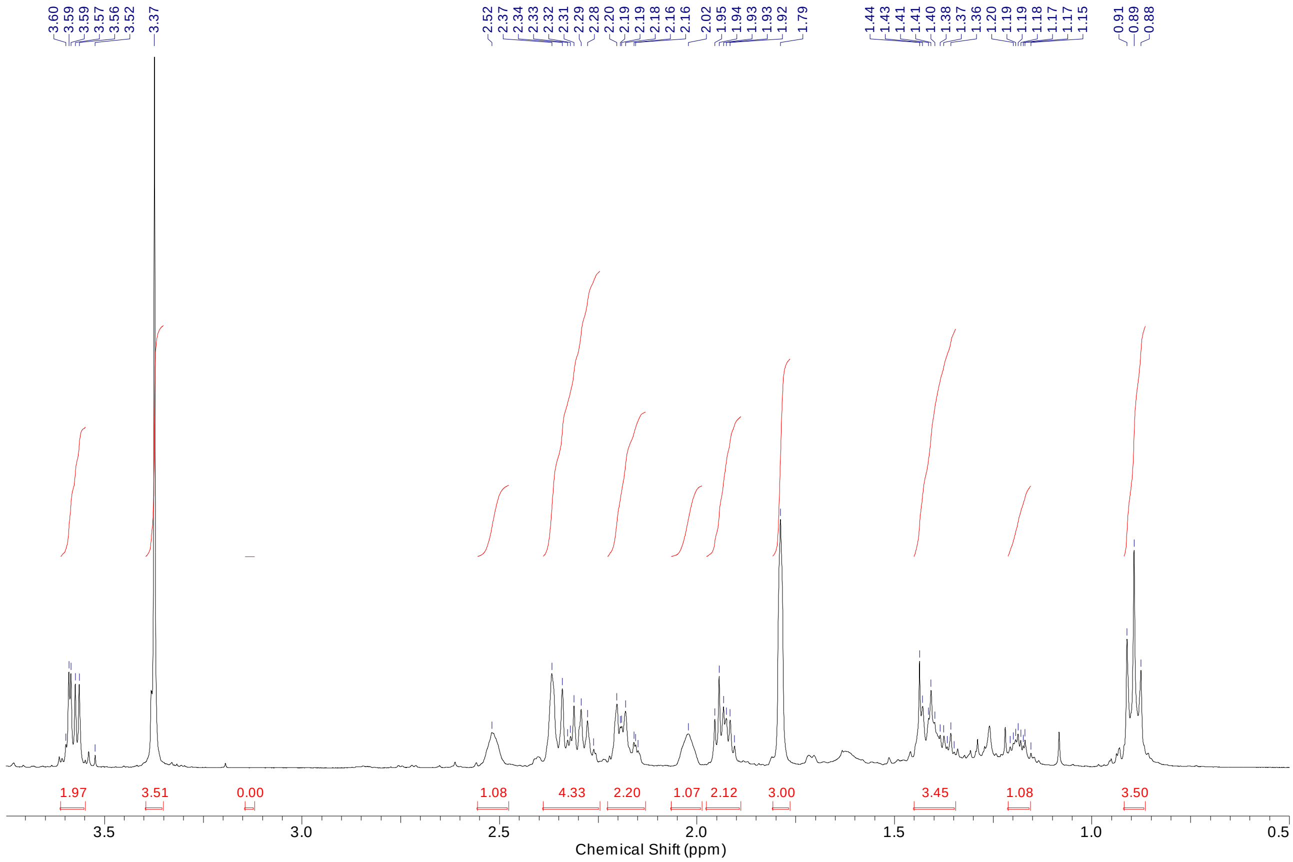

^1H NMR (400 MHz) spectrum ofdecalinone **24a** in $CDCl_3$ (0.5 – 3.75 ppm)

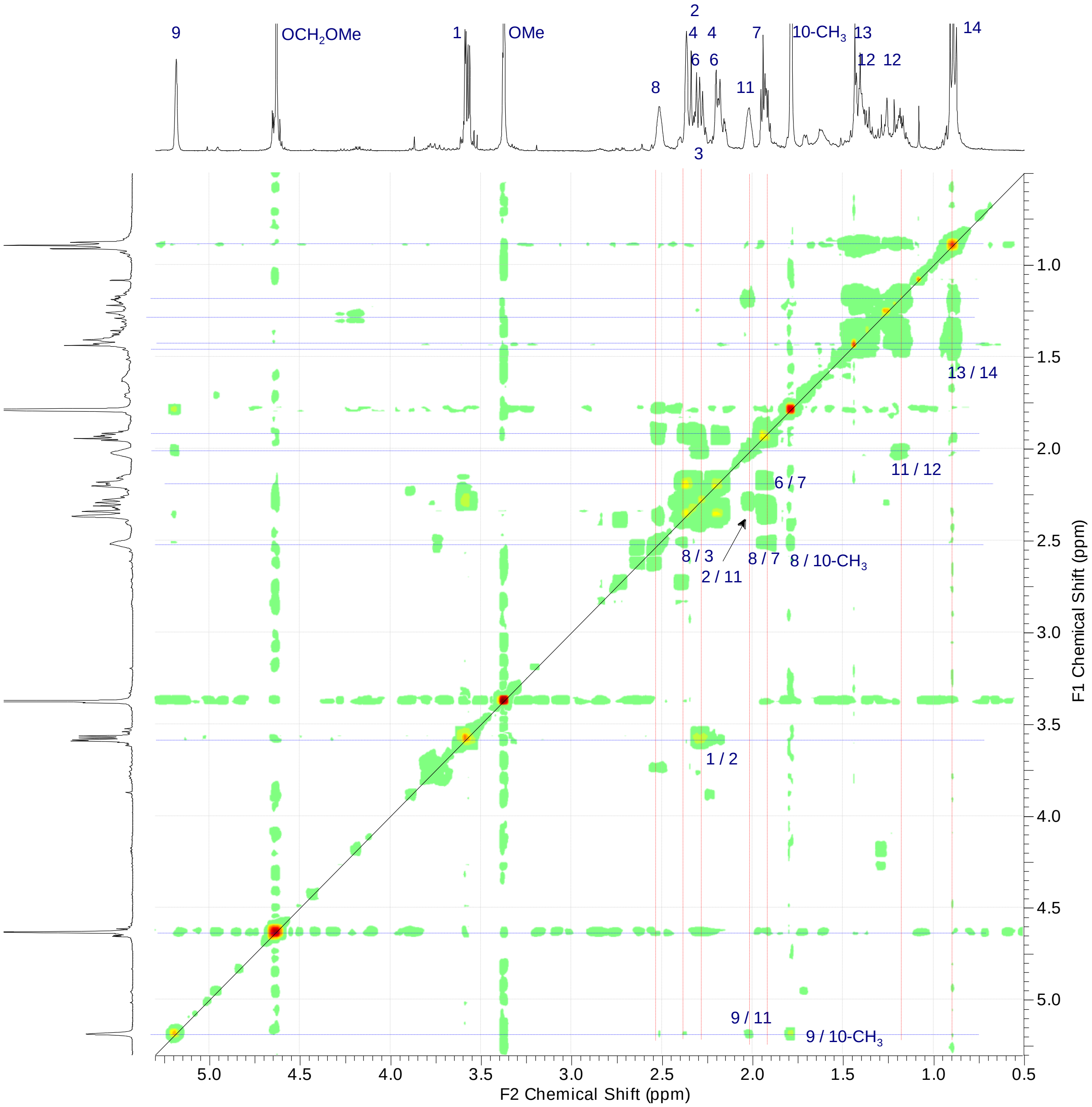

COSY spectrum of decalinone **24a** in $CDCl_3$ (0.5 – 5.3 ppm)

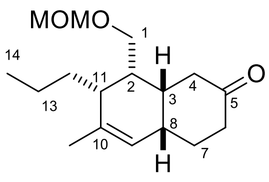

24a

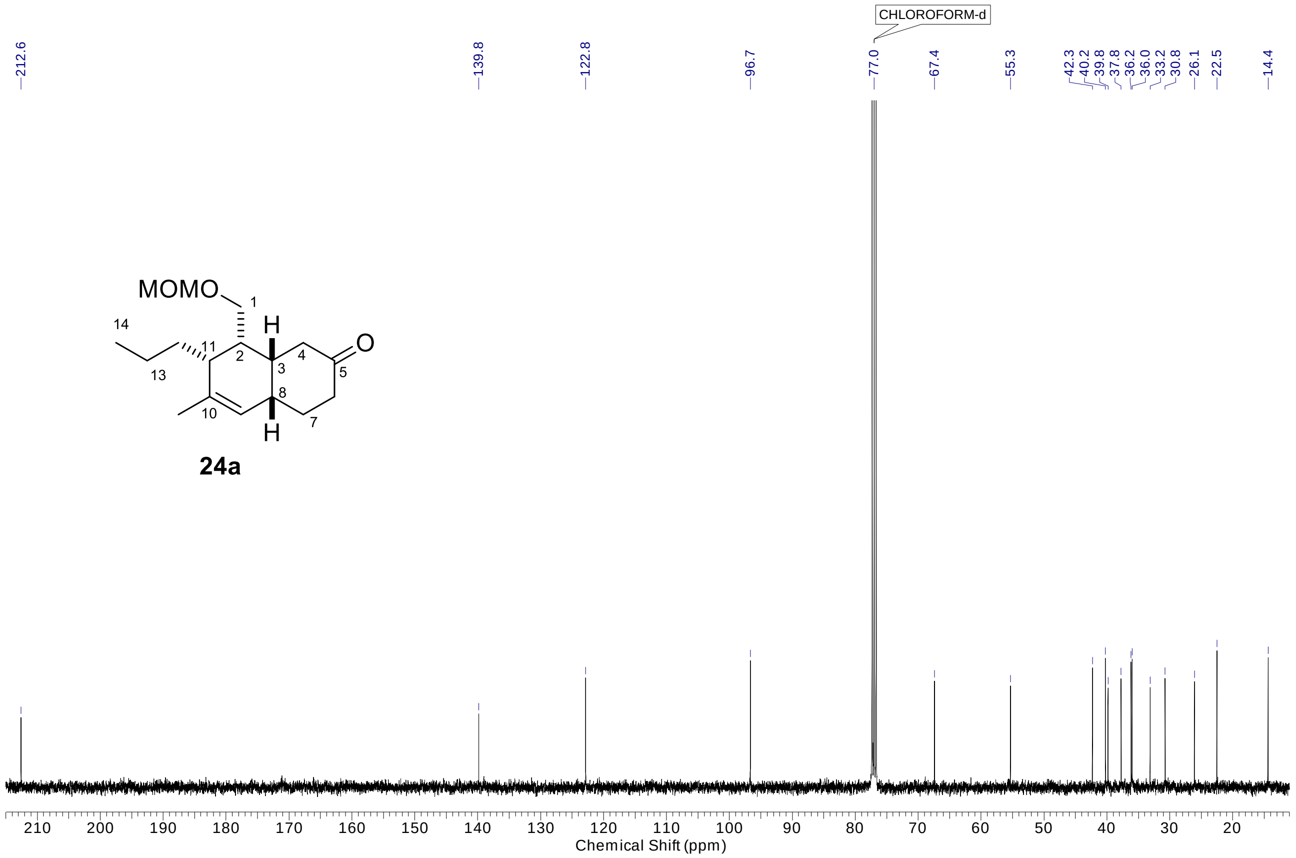

^{13}C NMR (100 MHz) spectrum of decalinone **24a** in $CDCl_3$ (10 – 215 ppm)

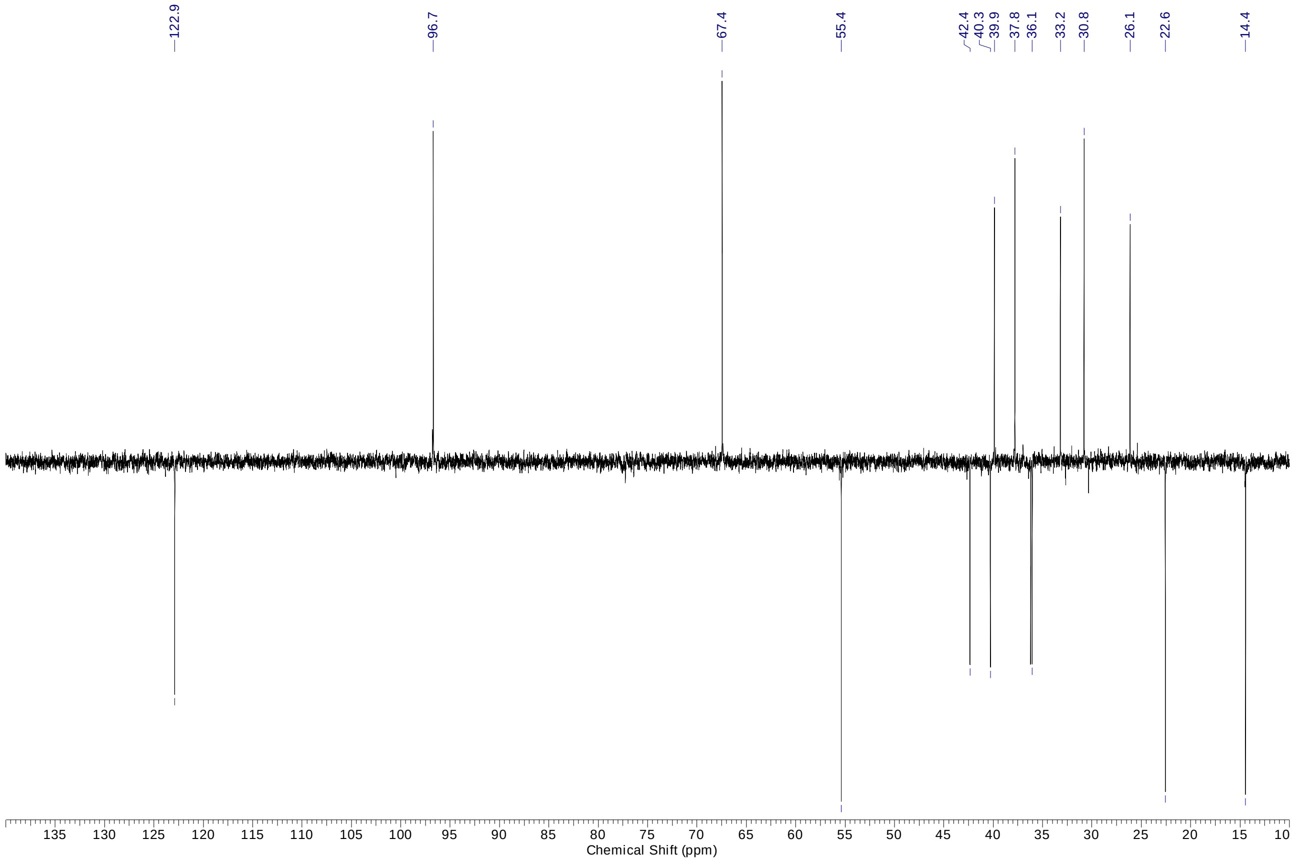

DEPT (100 MHz) spectrum of decalinone **24a** in $CDCl_3$ (10 – 140 ppm)

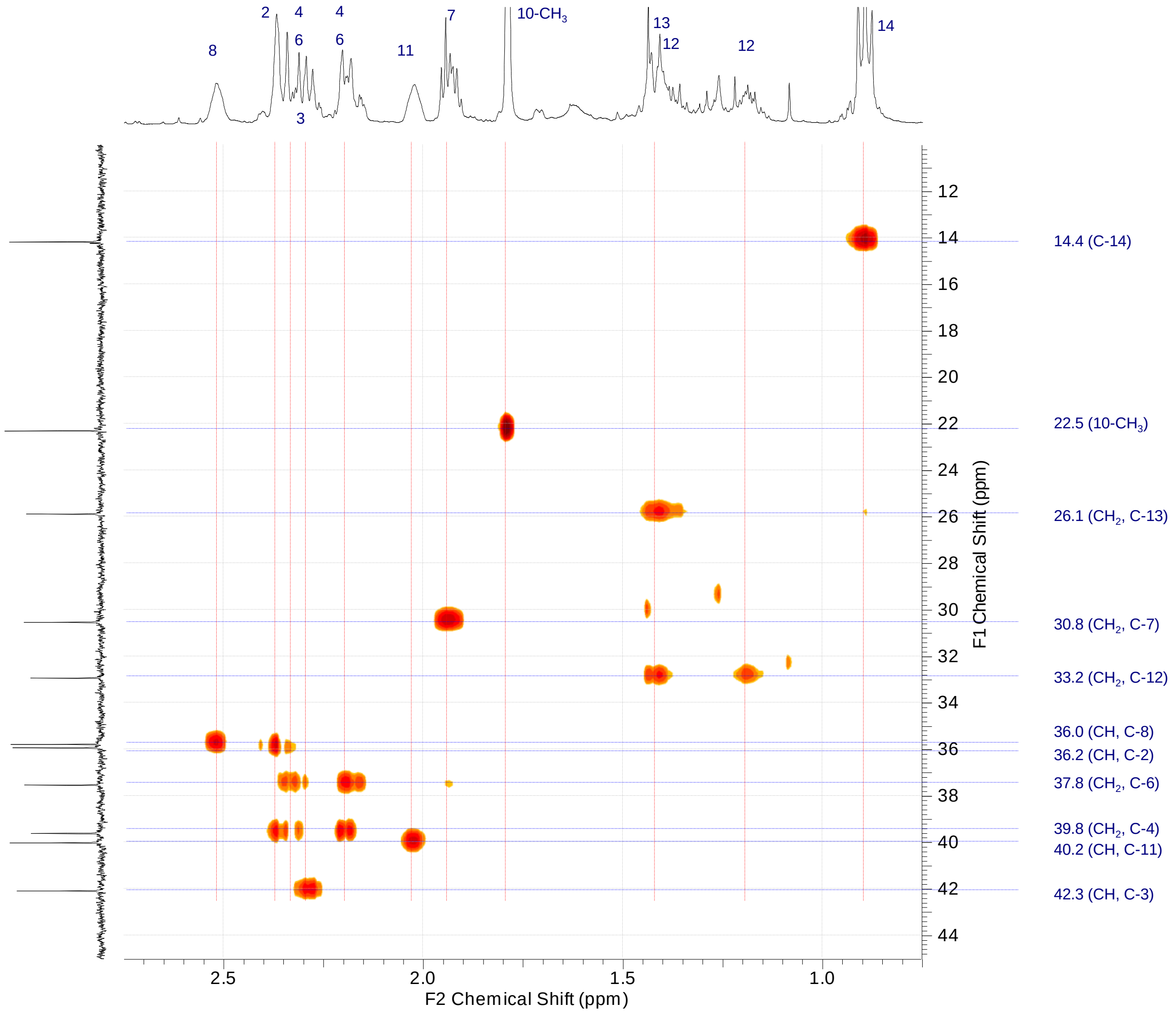

HSQC spectrum of decalinone **24a** in $CDCl_3$ (0.75 – 2.75, 10 – 45 ppm)

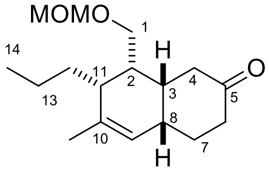

24a

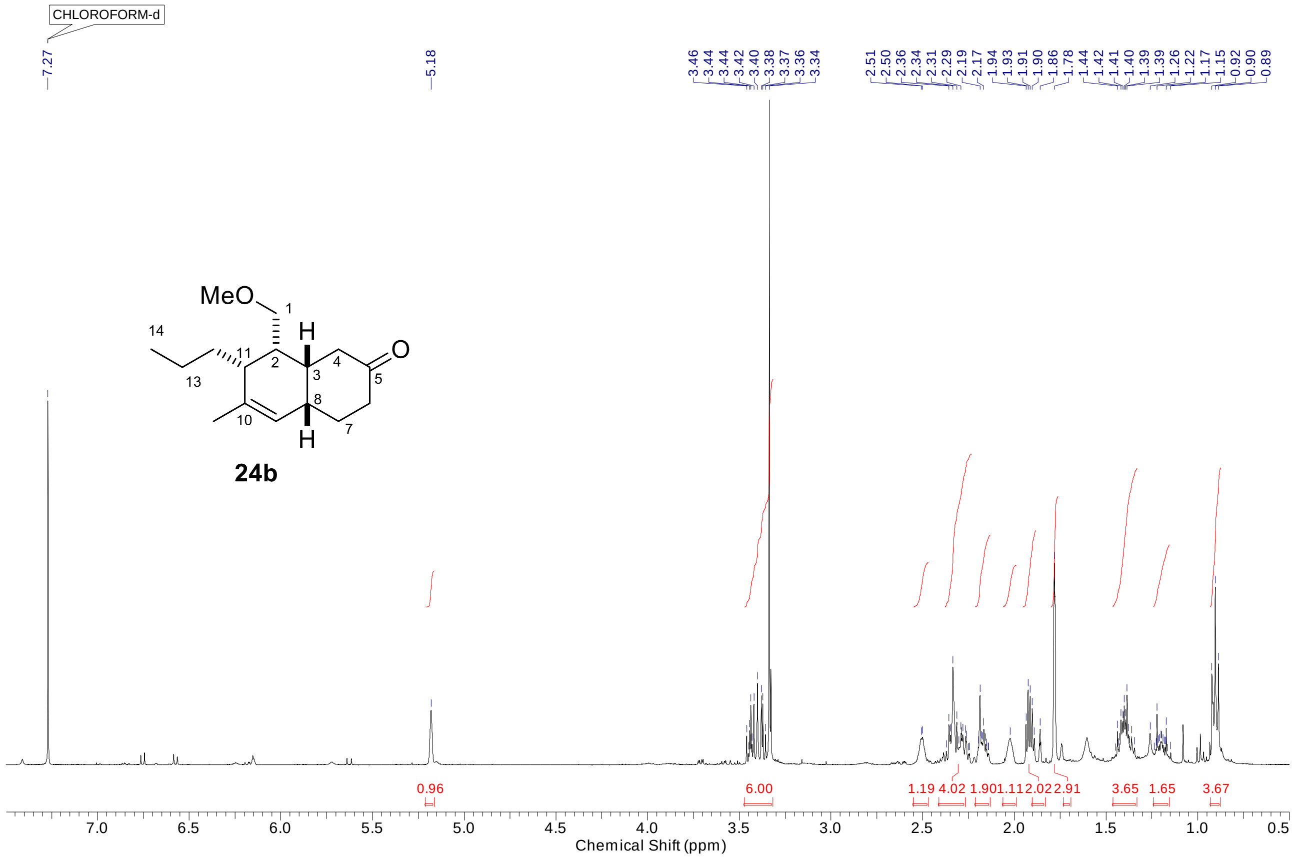

^{1}H NMR (400 MHz) spectrum of decalinone **24b** in $CDCl_3$ (0.5 – 7.5 ppm)

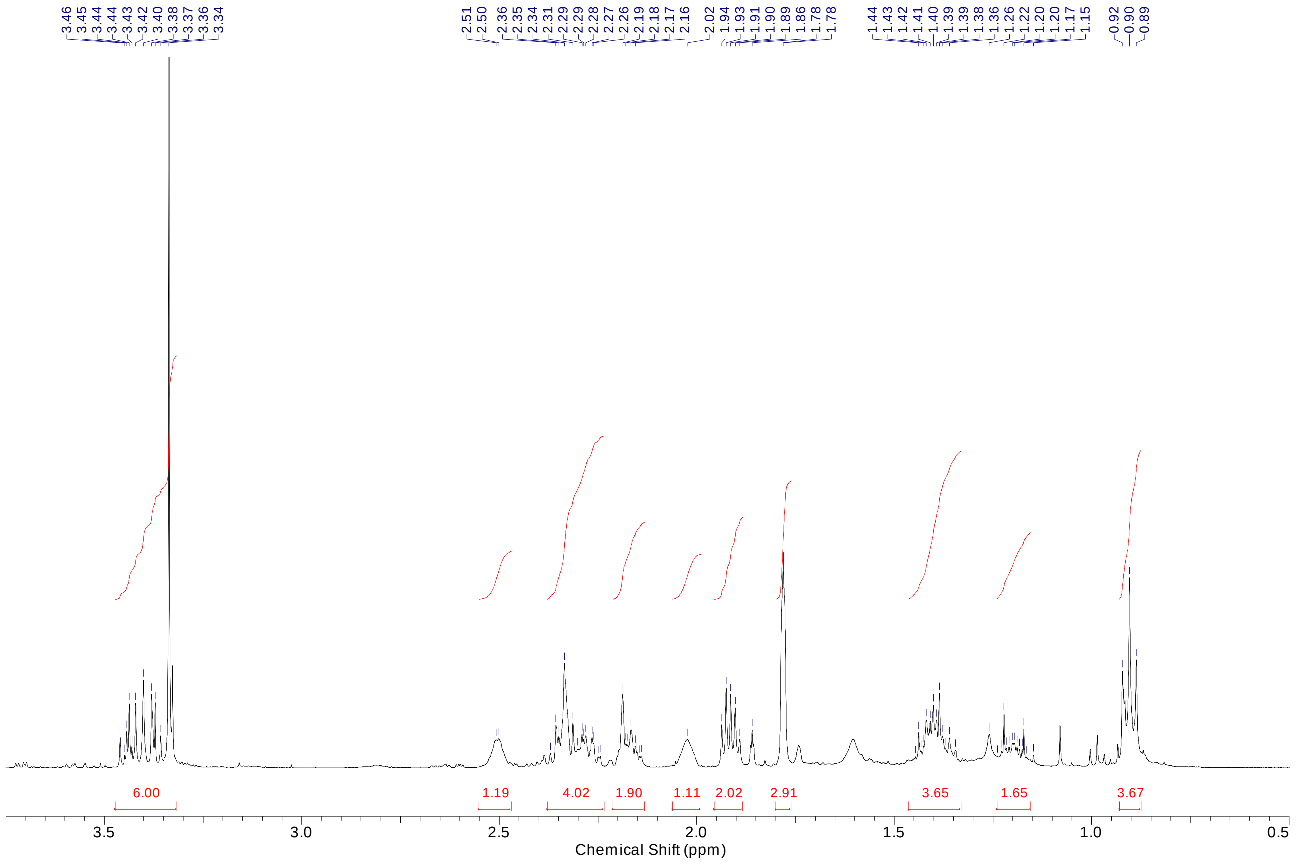

^{1}H NMR (400 MHz) spectrum of decalinone **24b** in $CDCl_3$ (0.5 – 3.75 ppm)

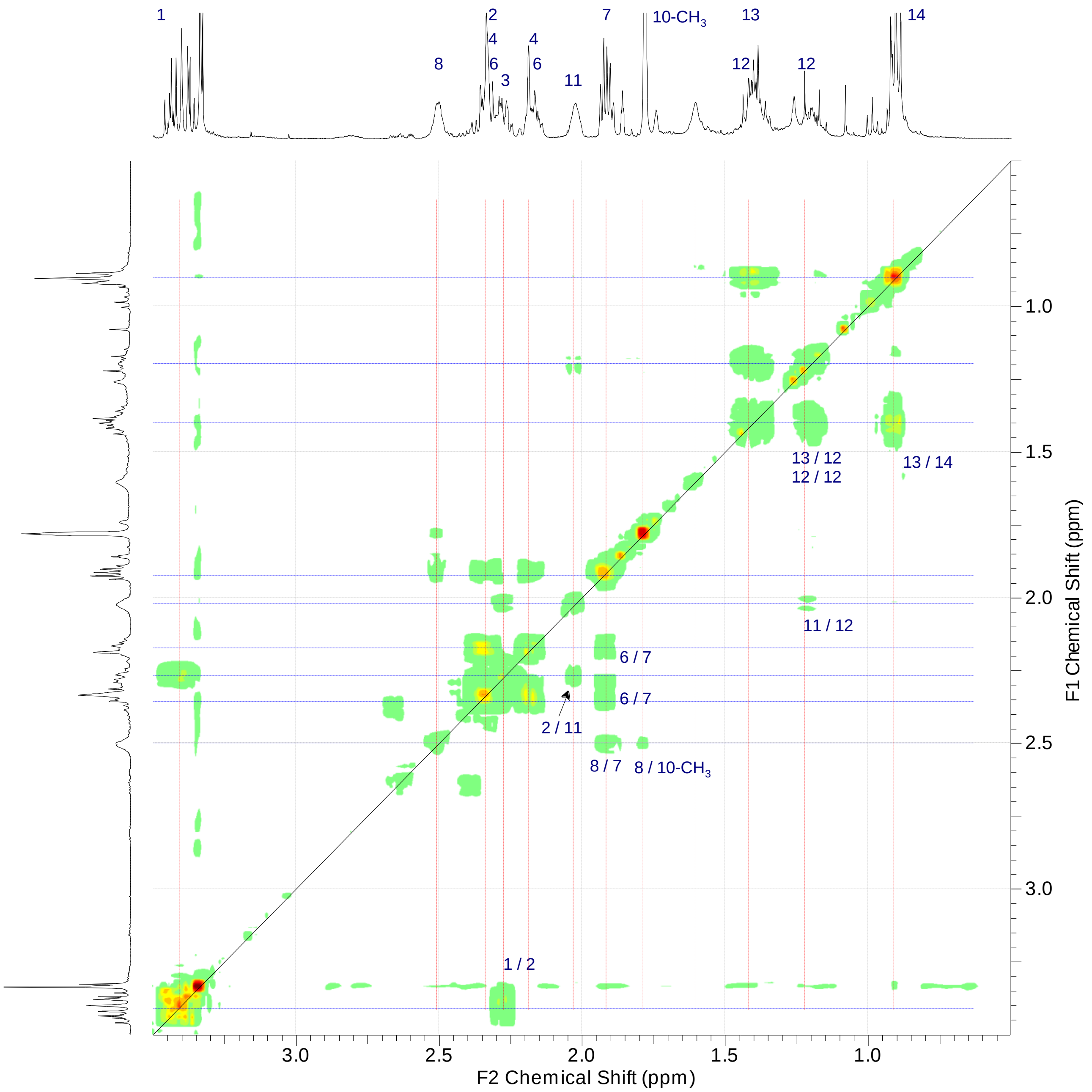

COSY spectrum of decalinone **24b** in $CDCl_3$ (0.5 – 3.5 ppm)

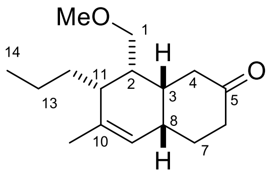

24b

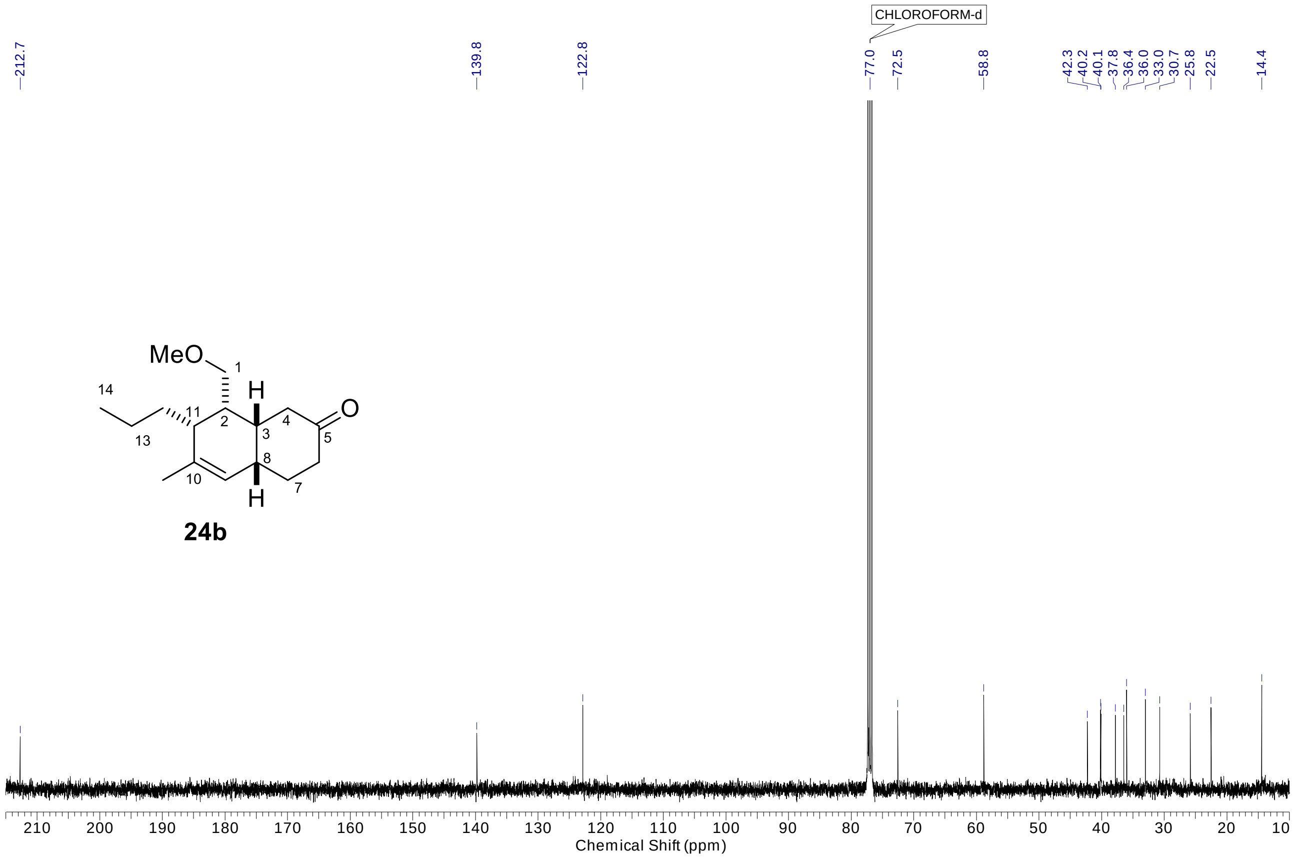

^{13}C NMR (100 MHz) spectrum of decalinone **24b** in $CDCl_3$ (10 – 215 ppm)

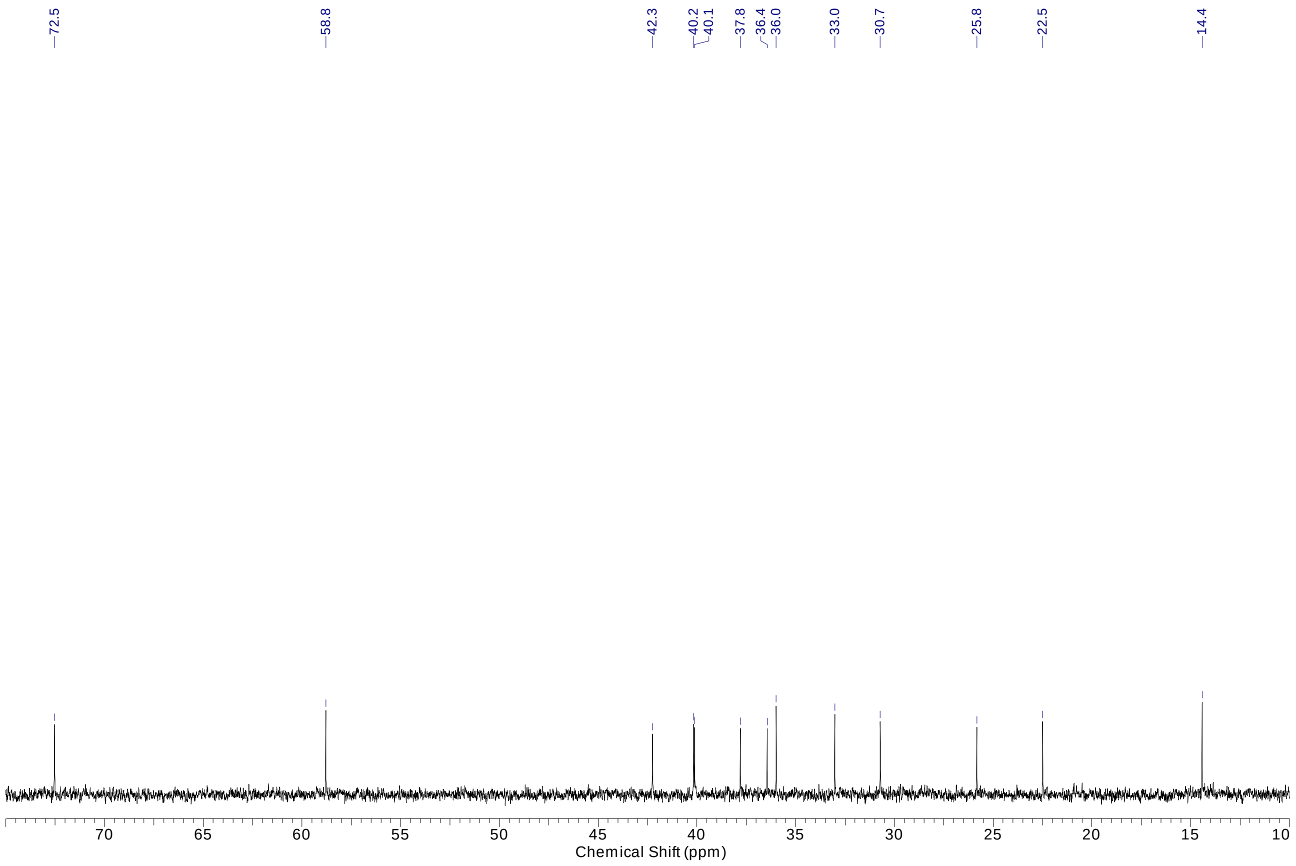

^{13}C NMR (100 MHz) spectrum of decalinone **24b** in $CDCl_3$ (10 – 75 ppm)

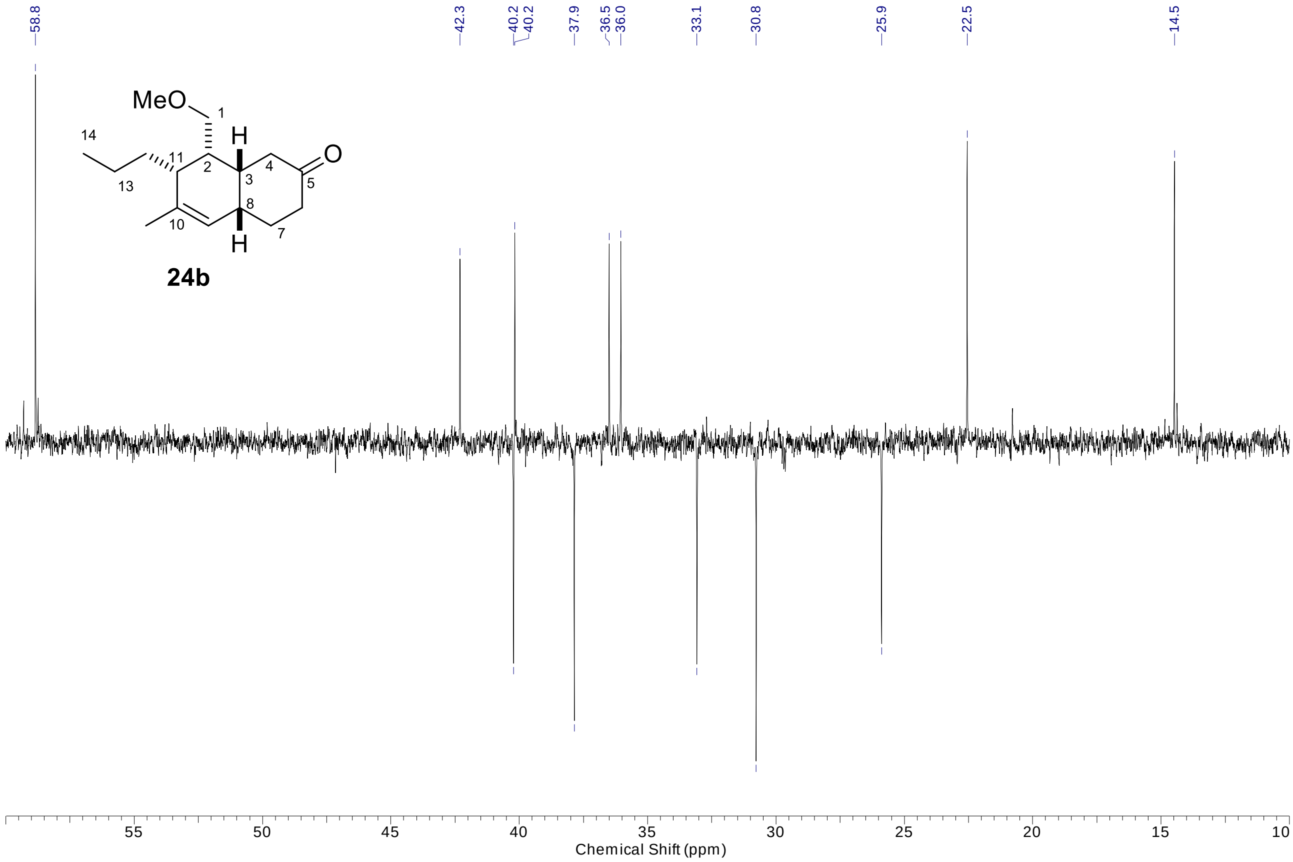

DEPT (100 MHz) spectrum of decalinone **24b** in $CDCl_3$ (10 – 60 ppm)

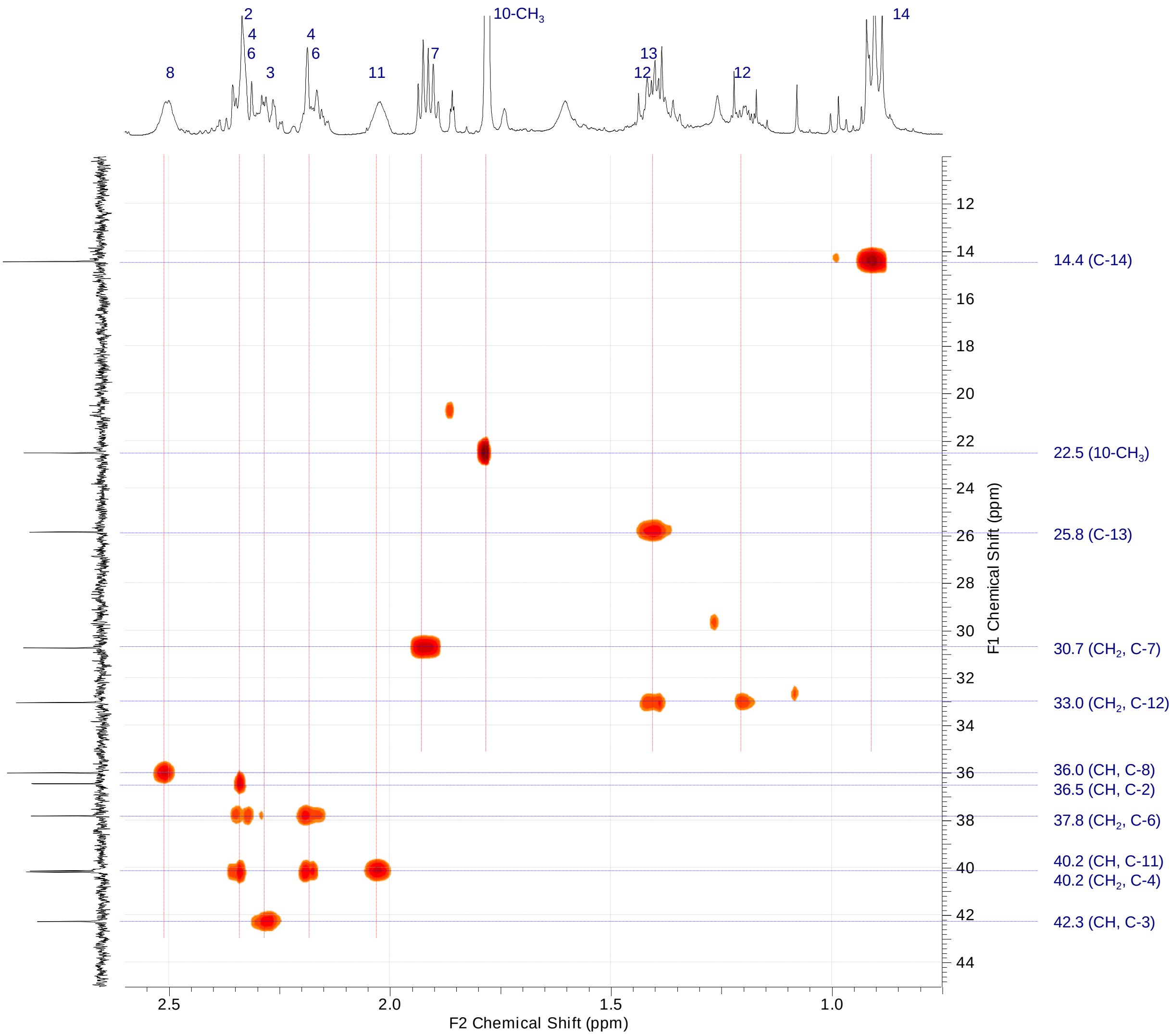

HSQC spectrum of decalinone **24b** in $CDCl_3$ (0.75 – 2.6, 10 – 45 ppm)

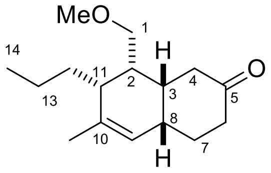

24b

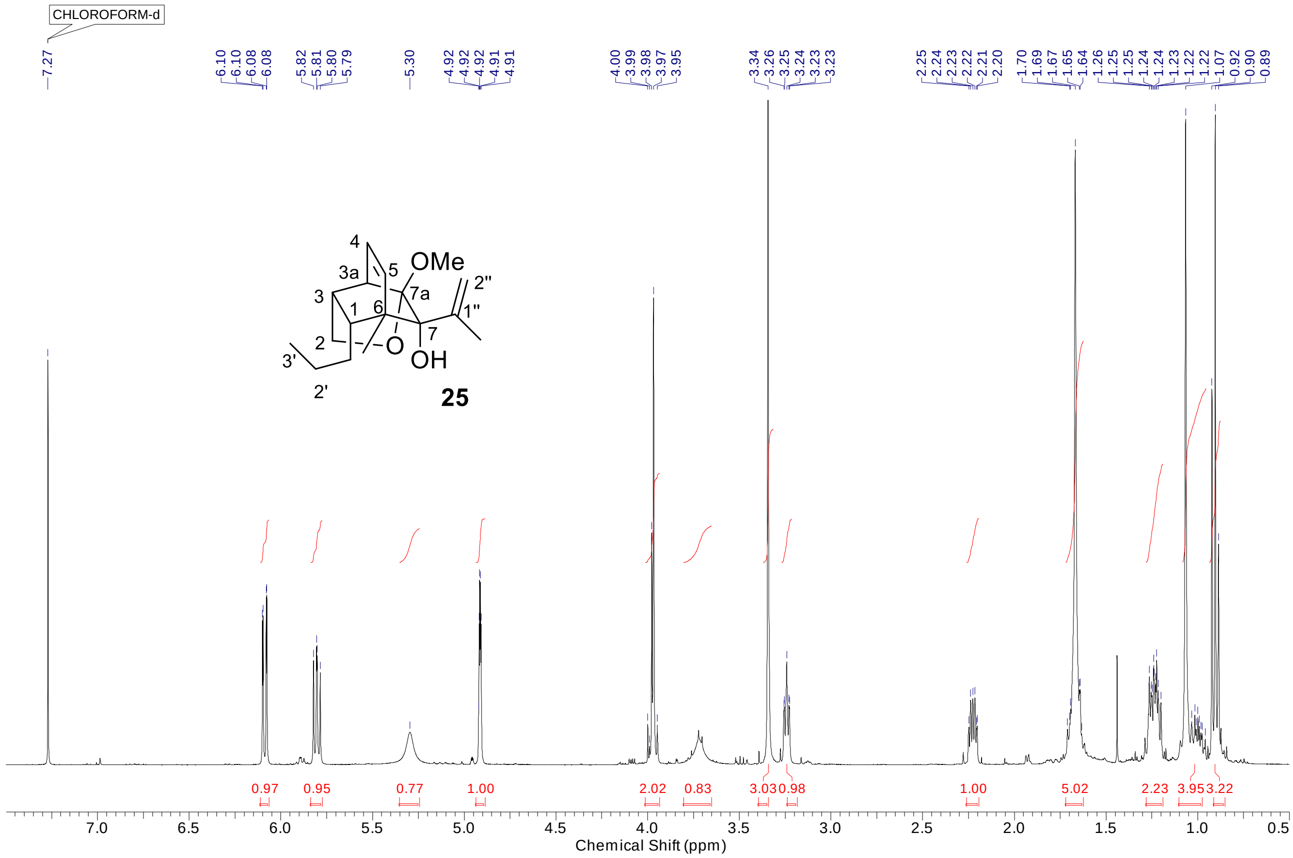

^{1}H NMR (400 MHz) spectrum of polycyclic alcohol **25** in $CDCl_3$ (0.5 – 7.5 ppm)

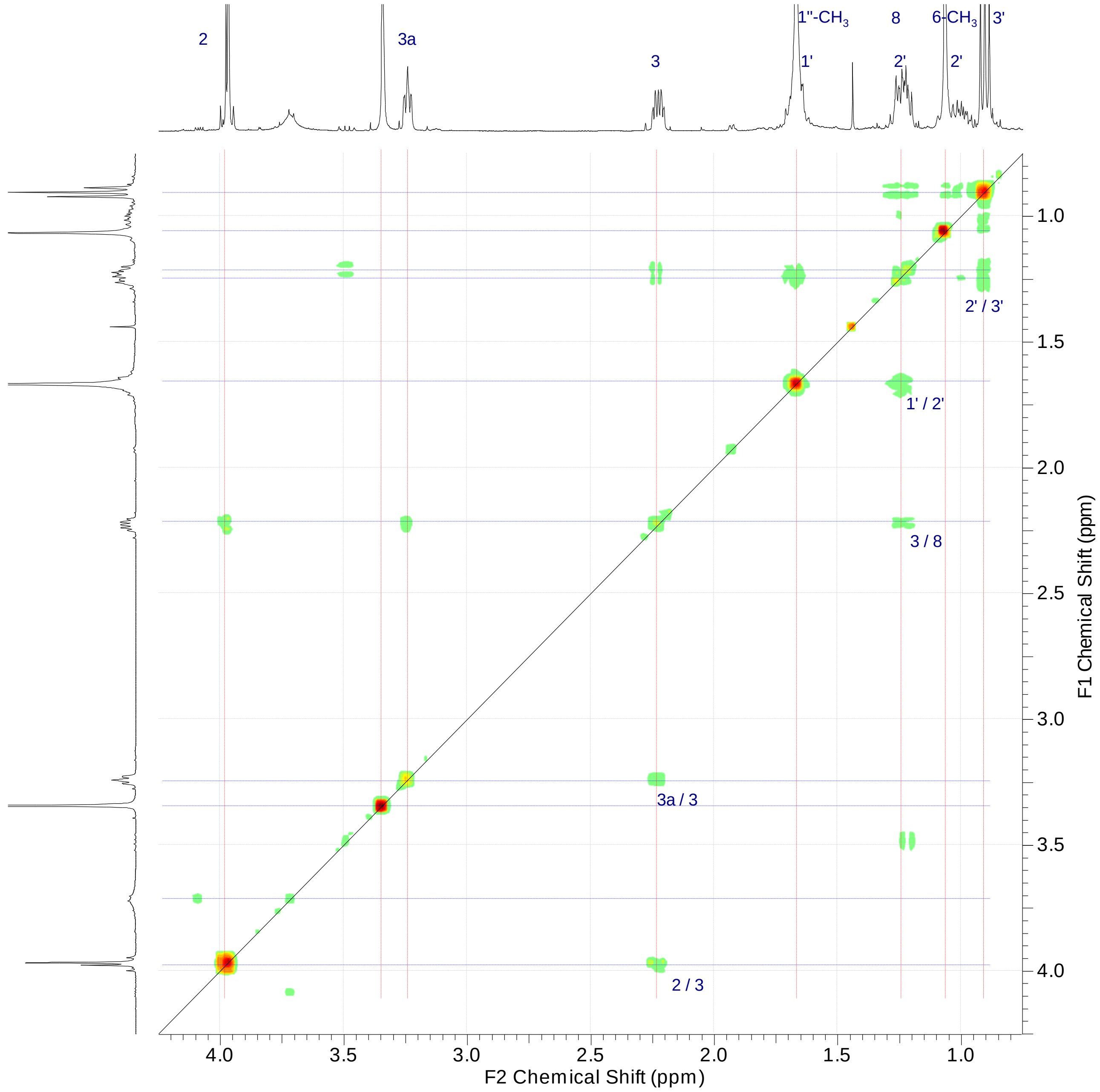

COSY spectrum of polycyclic alcohol **25** in $CDCl_3$ (0.75 – 4.25 ppm)

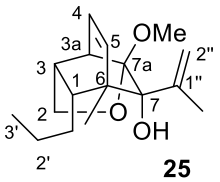

25

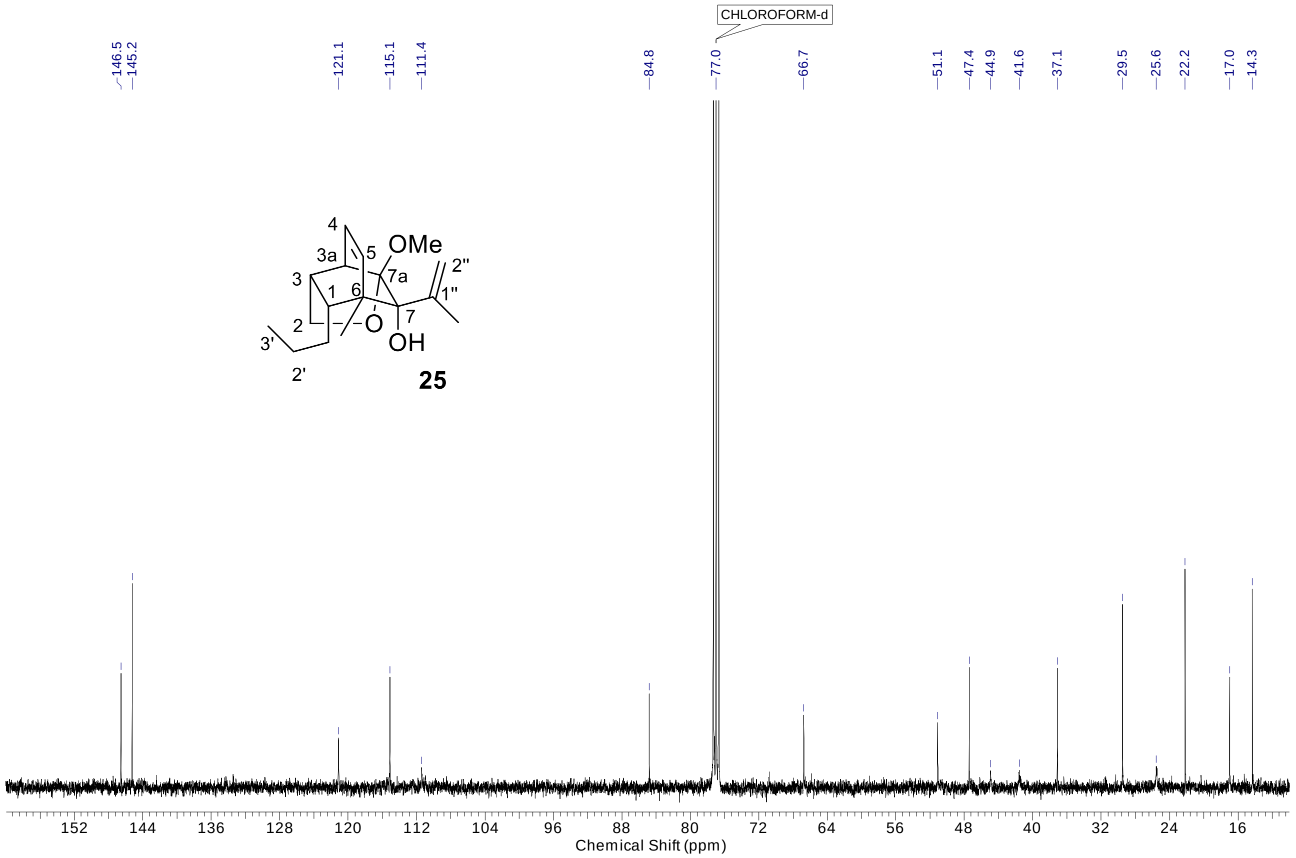

^{13}C NMR (100 MHz) spectrum of polycyclic alcohol **25** in $CDCl_3$ (10 – 160 ppm)

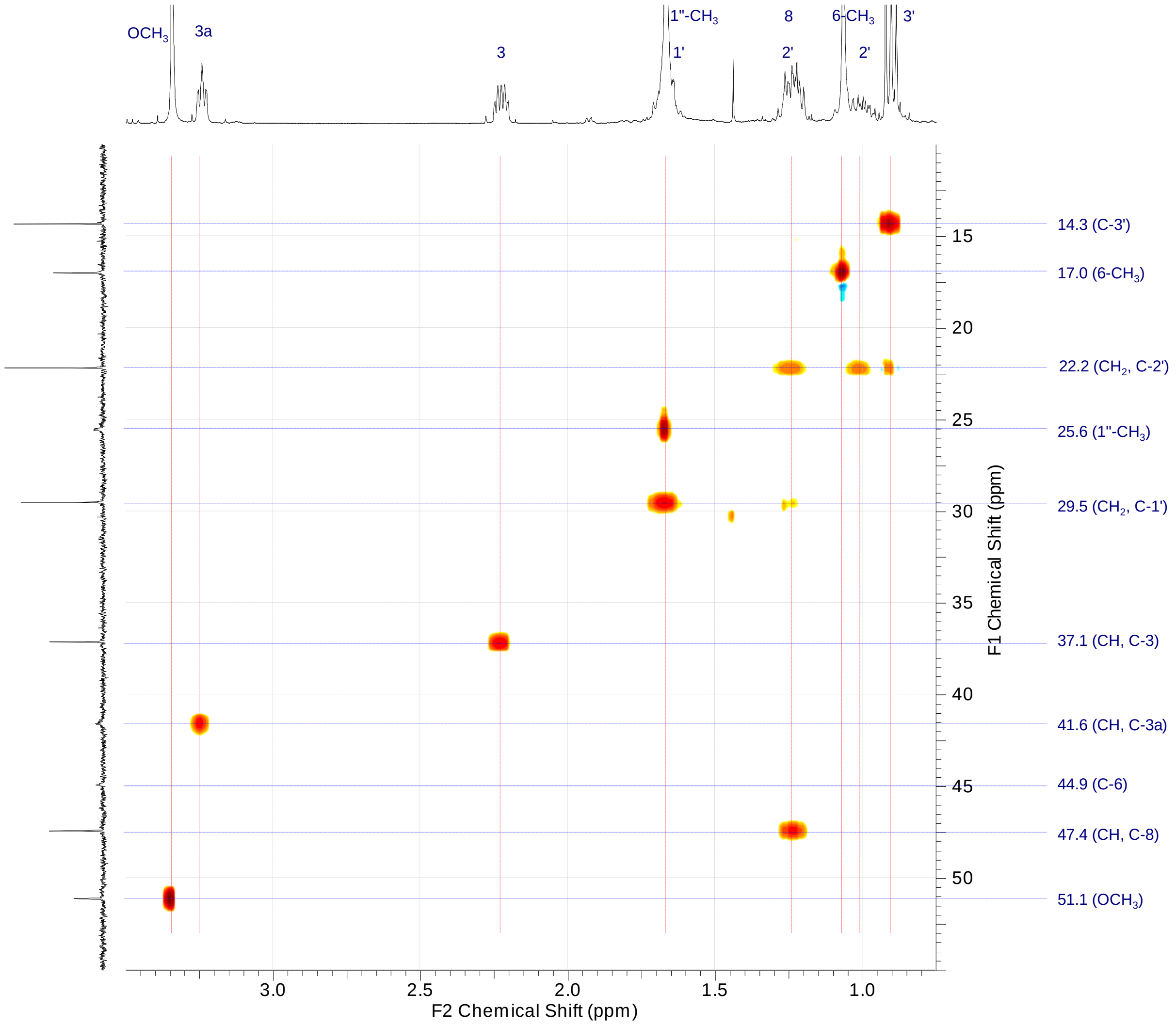

HSQC spectrum of polycyclic alcohol **25** in $CDCl_3$ (0.75 – 3.5, 10 – 55 ppm)

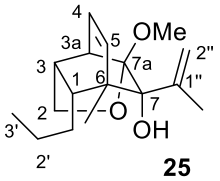

25

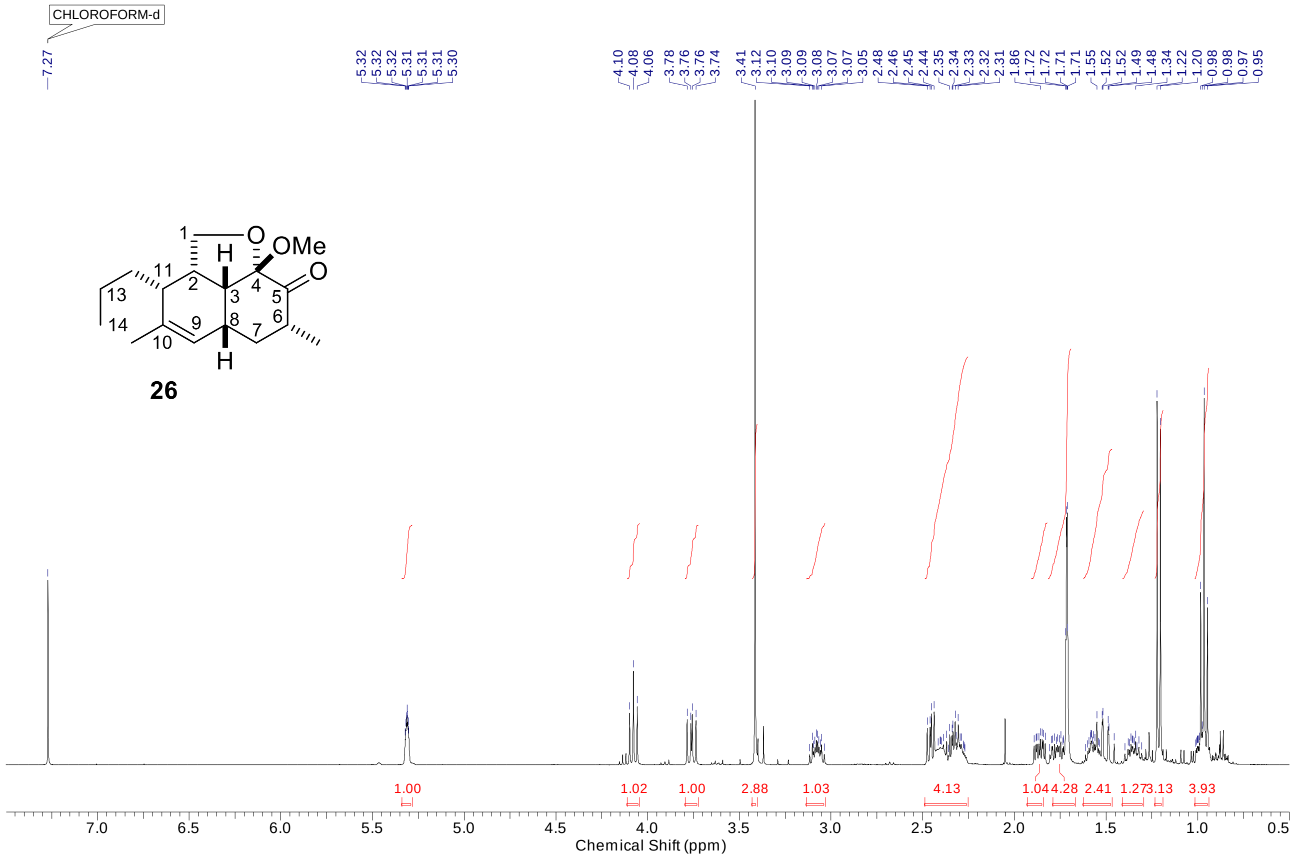

^{1}H NMR (400 MHz) spectrum of decalinone **26** in $CDCl_3$ (0.5 – 7.5 ppm)

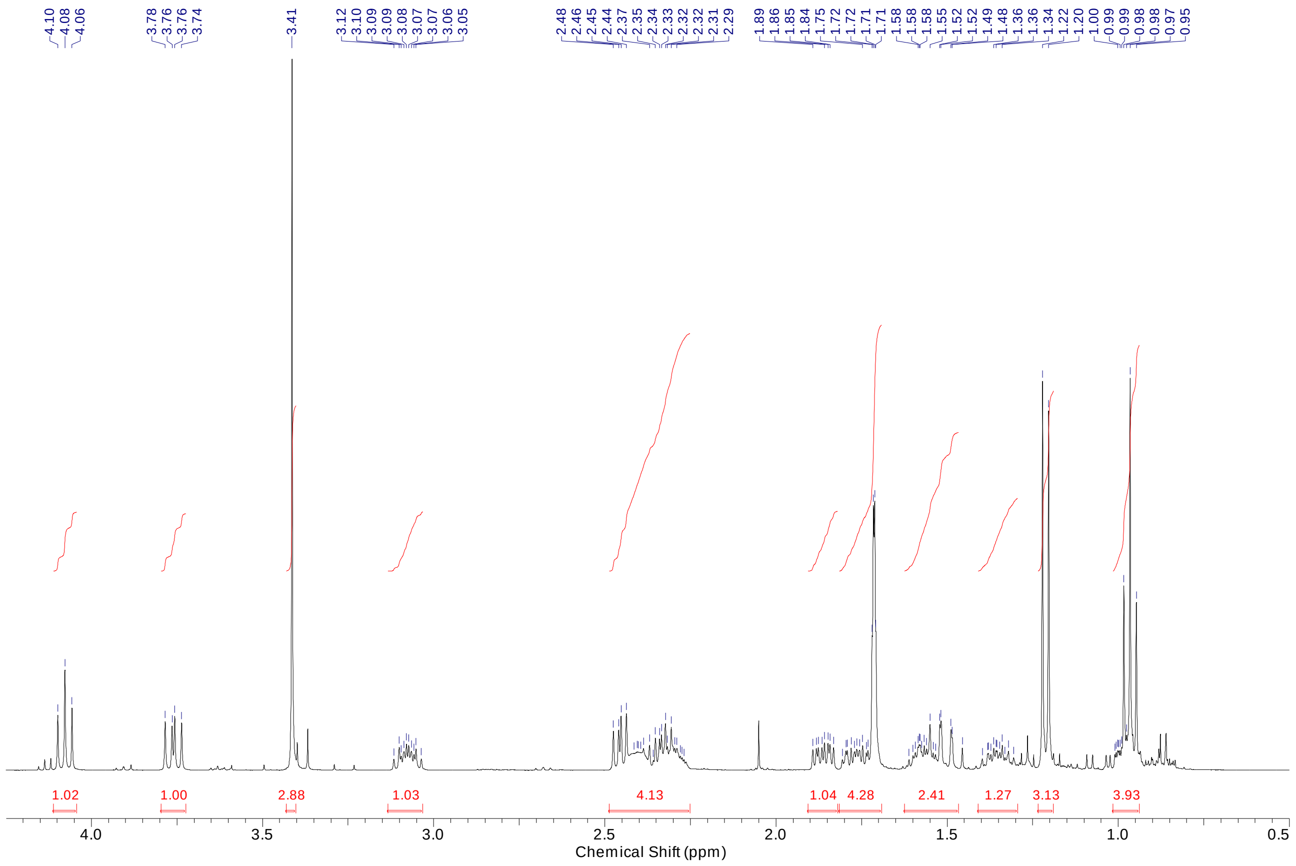

^{1}H NMR (400 MHz) spectrum of decalinone **26** in $CDCl_3$ (0.5 – 4.25 ppm)

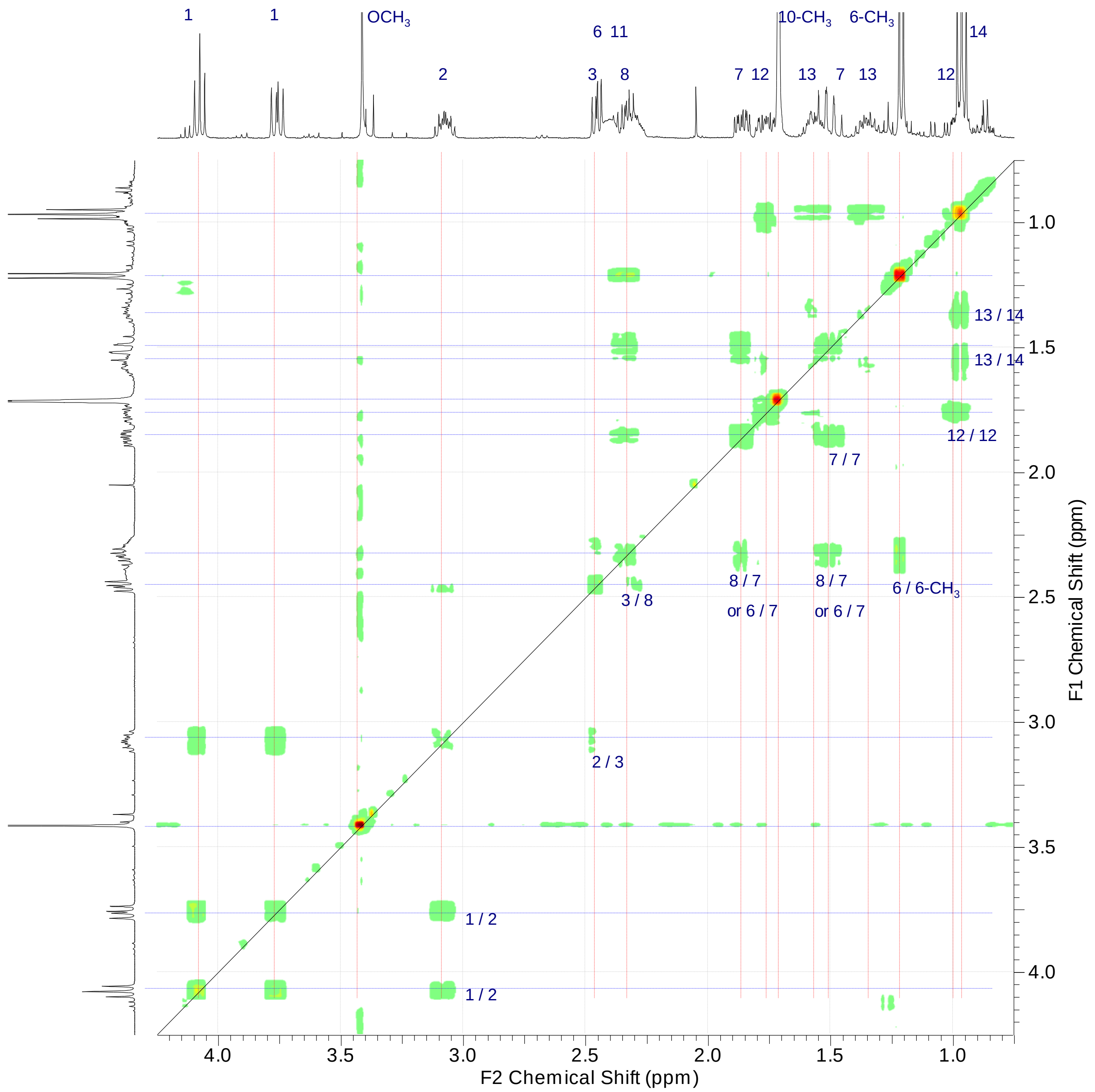

COSY spectrum of polycyclic decalinone **26** in $CDCl_3$ (0.75 – 4.25 ppm)

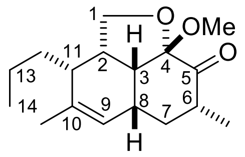

26

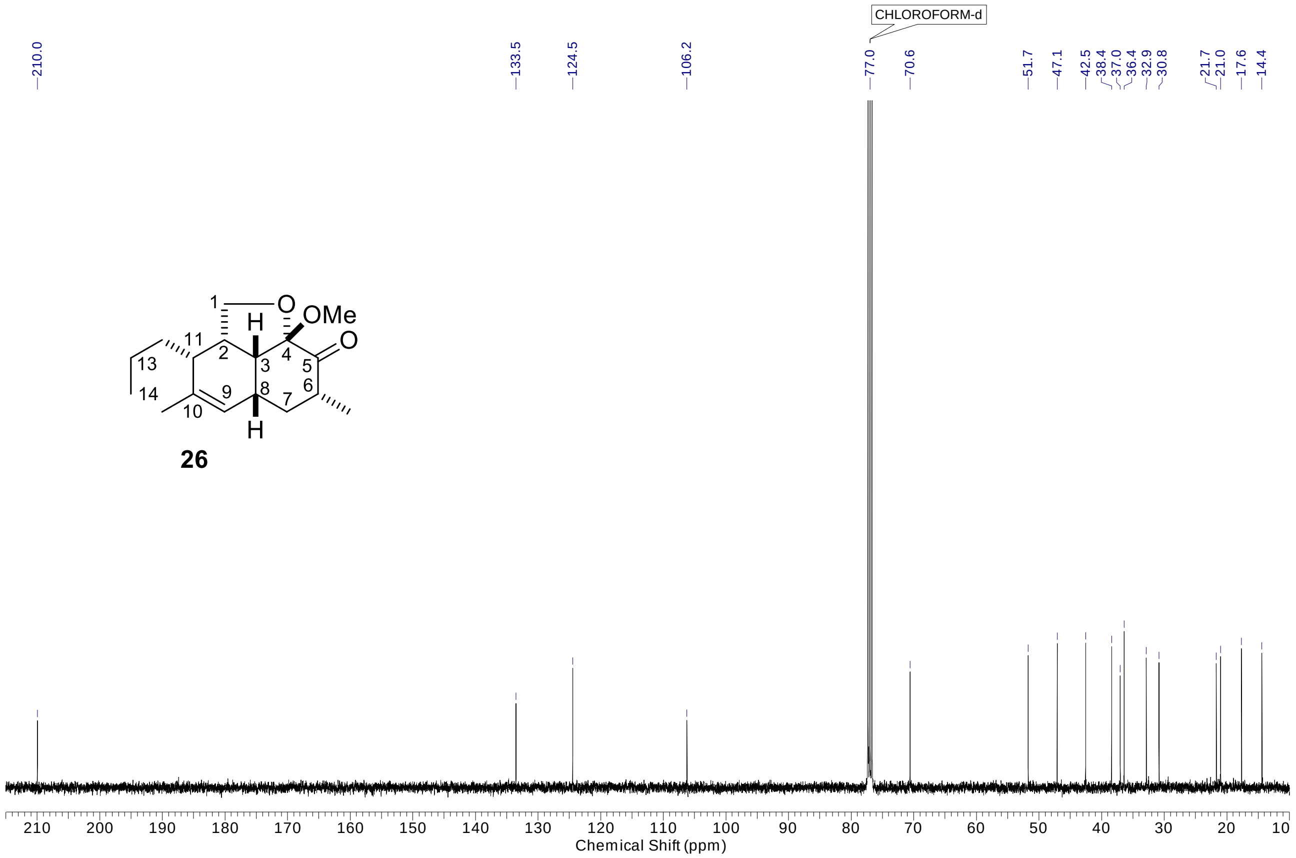

^{13}C NMR (100 MHz) spectrum of decalinone **26** in $CDCl_3$ (10 – 215 ppm)

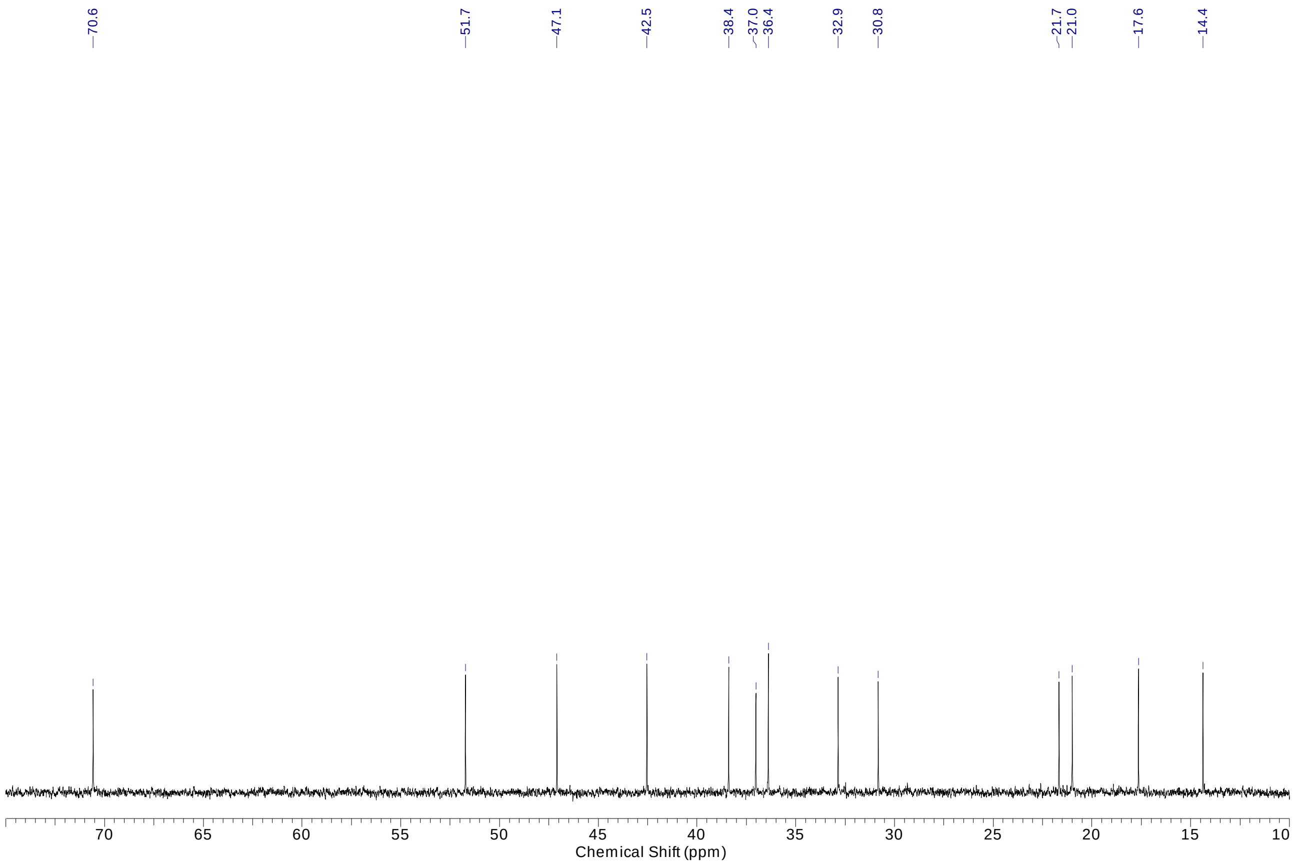

^{13}C NMR (100 MHz) spectrum of decalinone **26** in $CDCl_3$ (10 – 75 ppm)

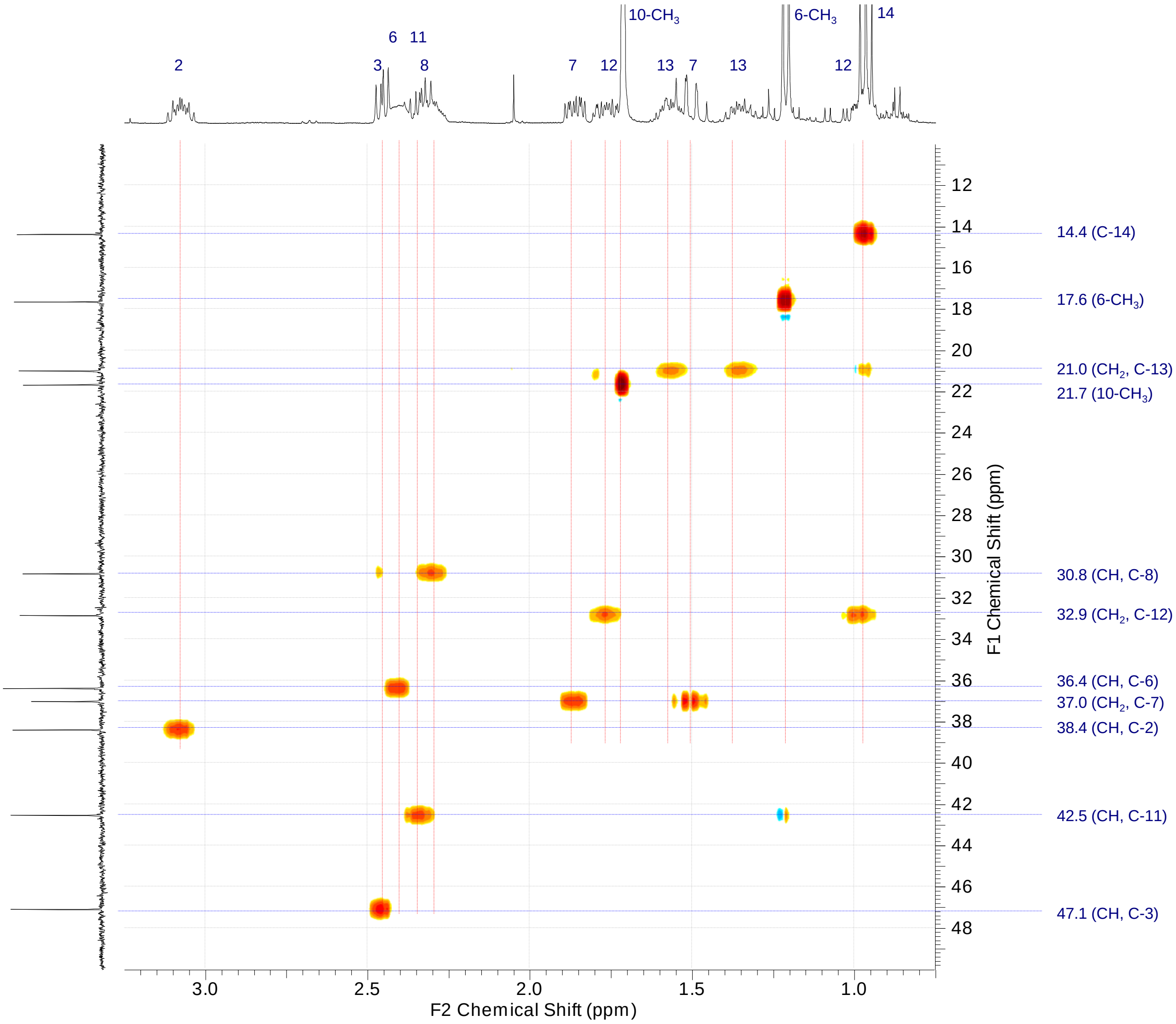

HSQC spectrum of decalinone **26** in $CDCl_3$ (0.75 – 3.25, 10 – 50 ppm)

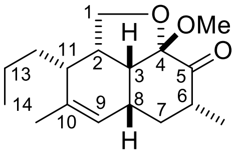

26

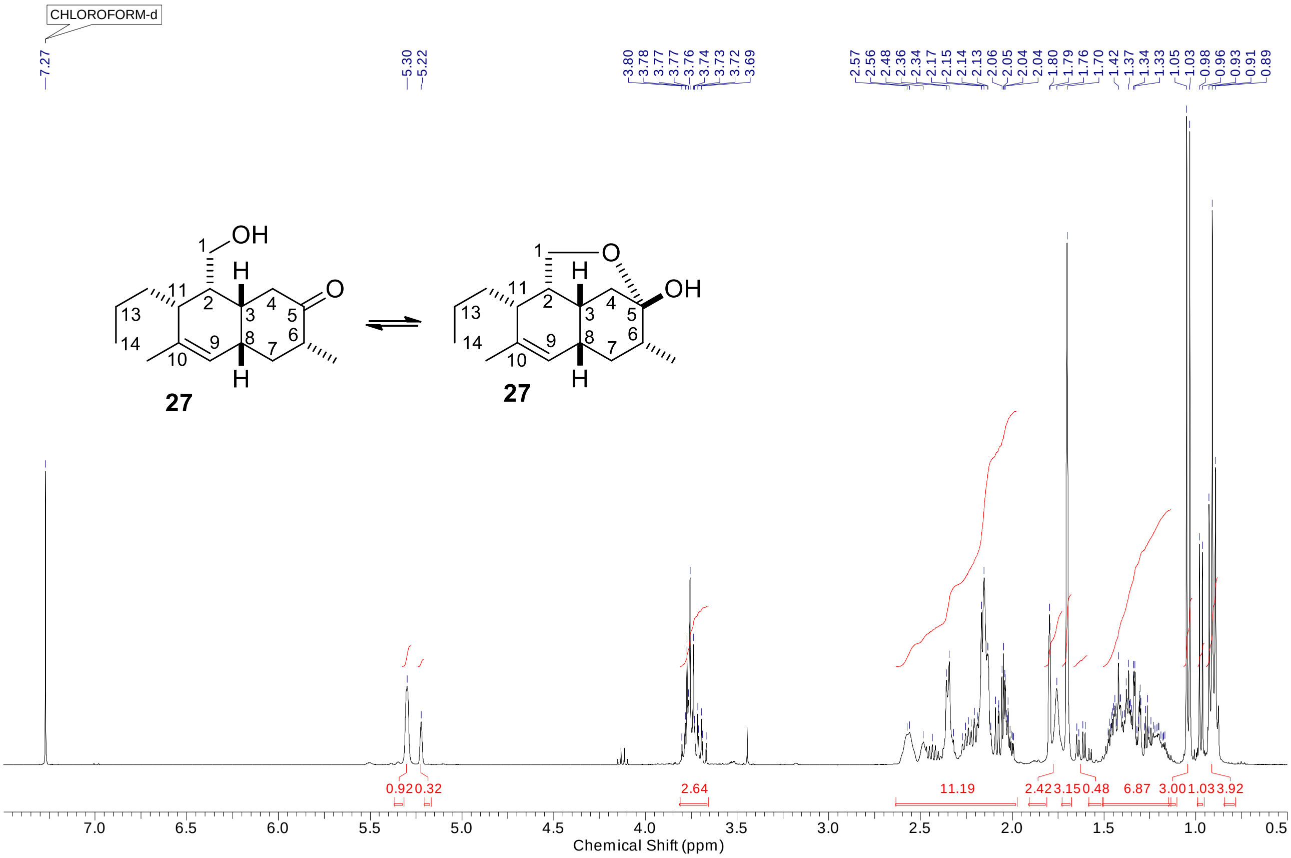

^{1}H NMR (400 MHz) spectrum of decalinone **27** in $CDCl_3$ (0.5 – 7.5 ppm)

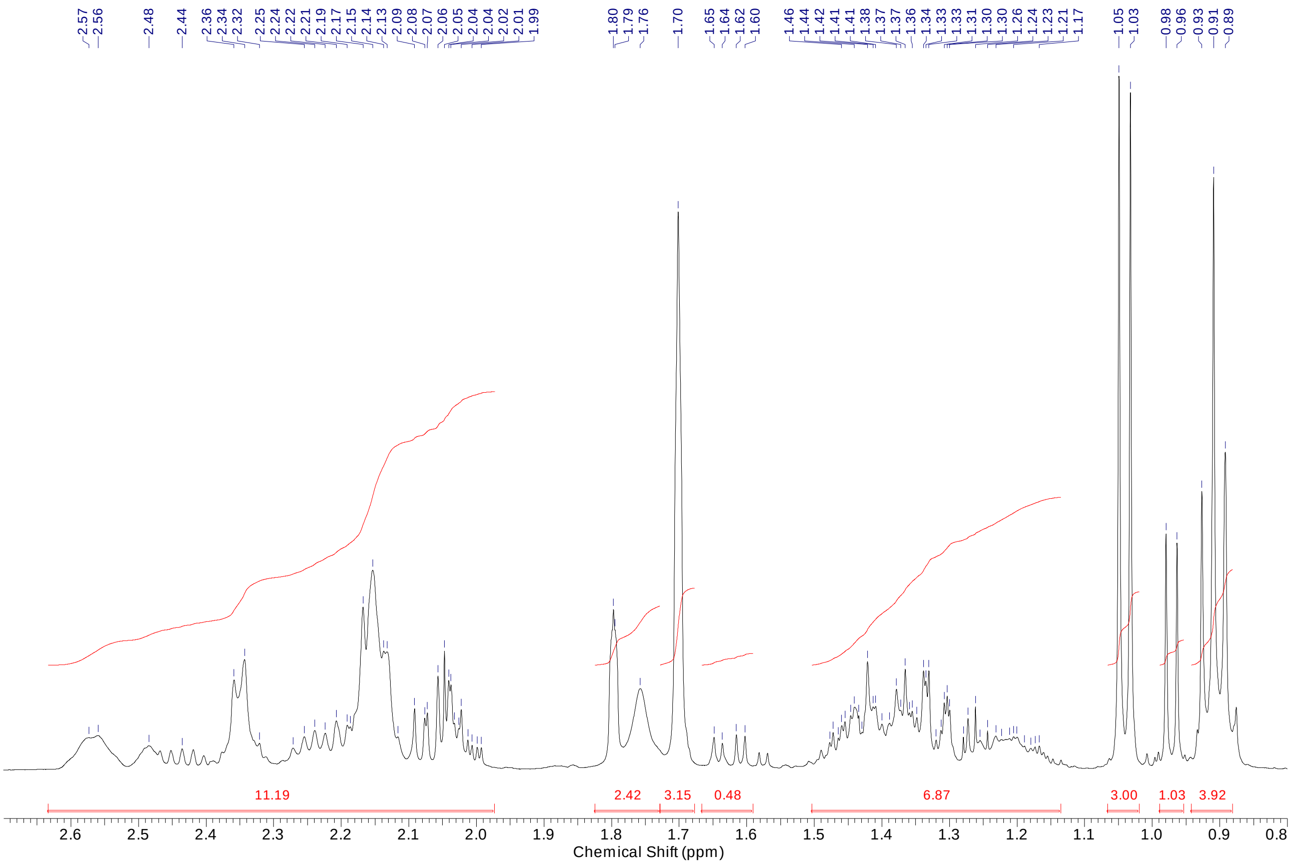

^{1}H NMR (400 MHz) spectrum of decalinone **27** in $CDCl_3$ (0.8 – 2.7 ppm)

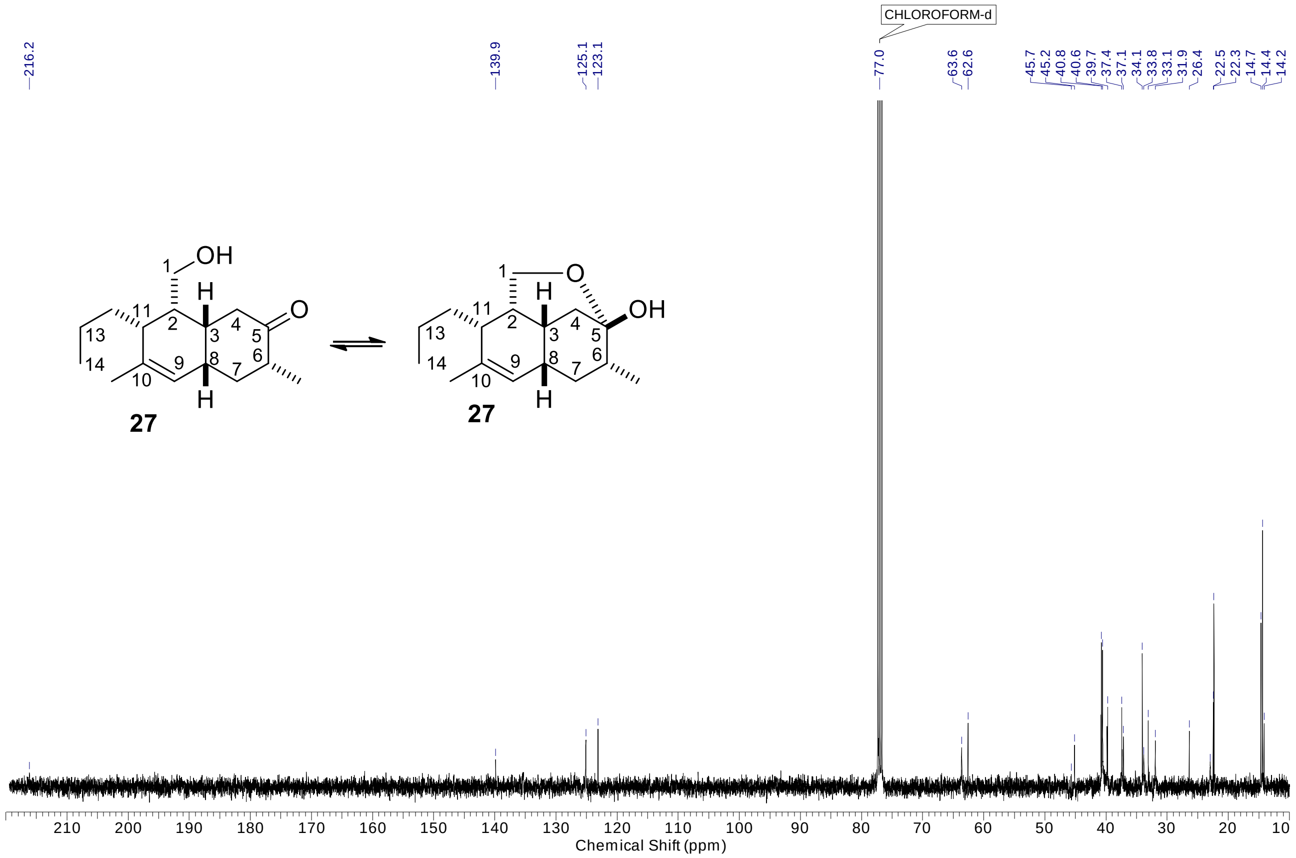

^{13}C NMR (100 MHz) spectrum of decalinone **27** in $CDCl_3$ (10 – 220 ppm)

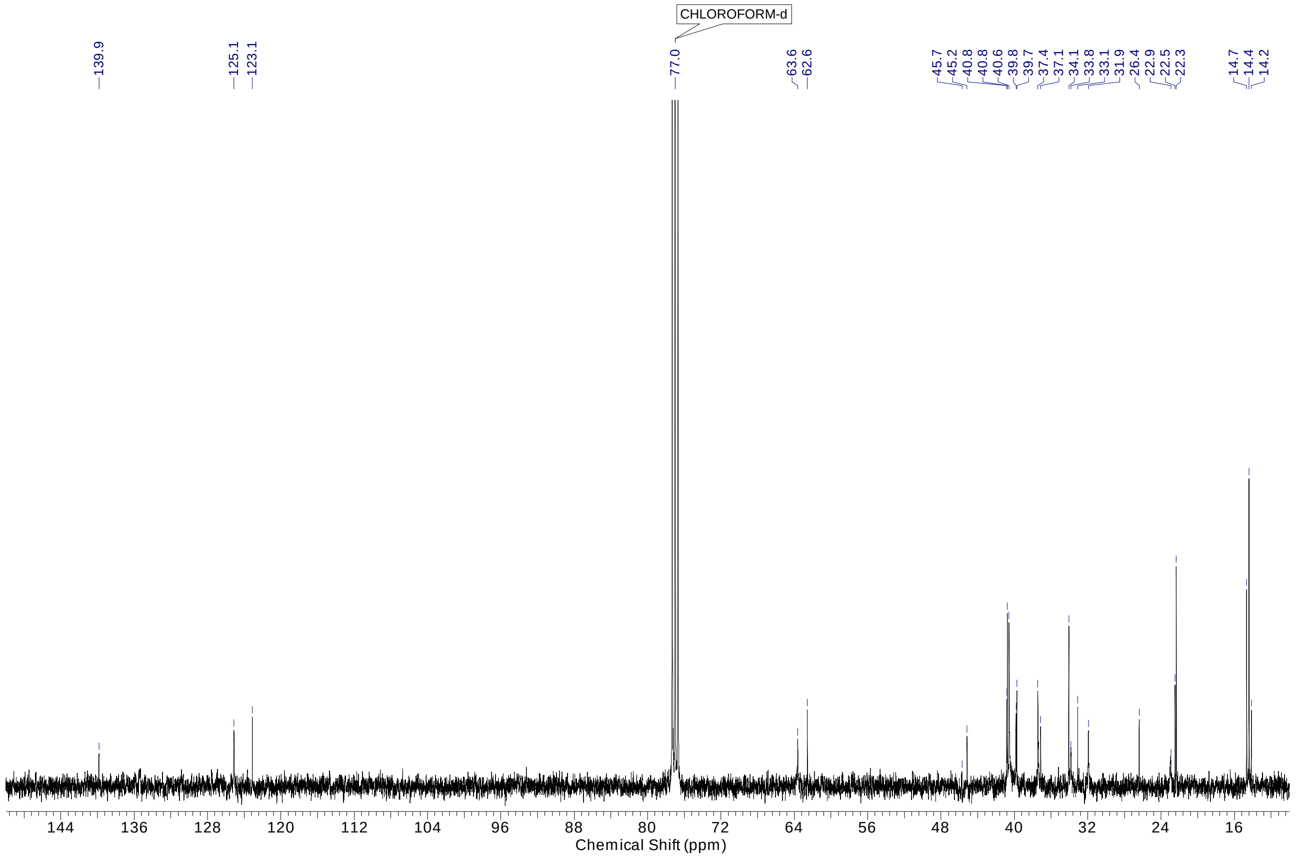

^{13}C NMR (100 MHz) spectrum of decalinone **27** in $CDCl_3$ (10 – 150 ppm)

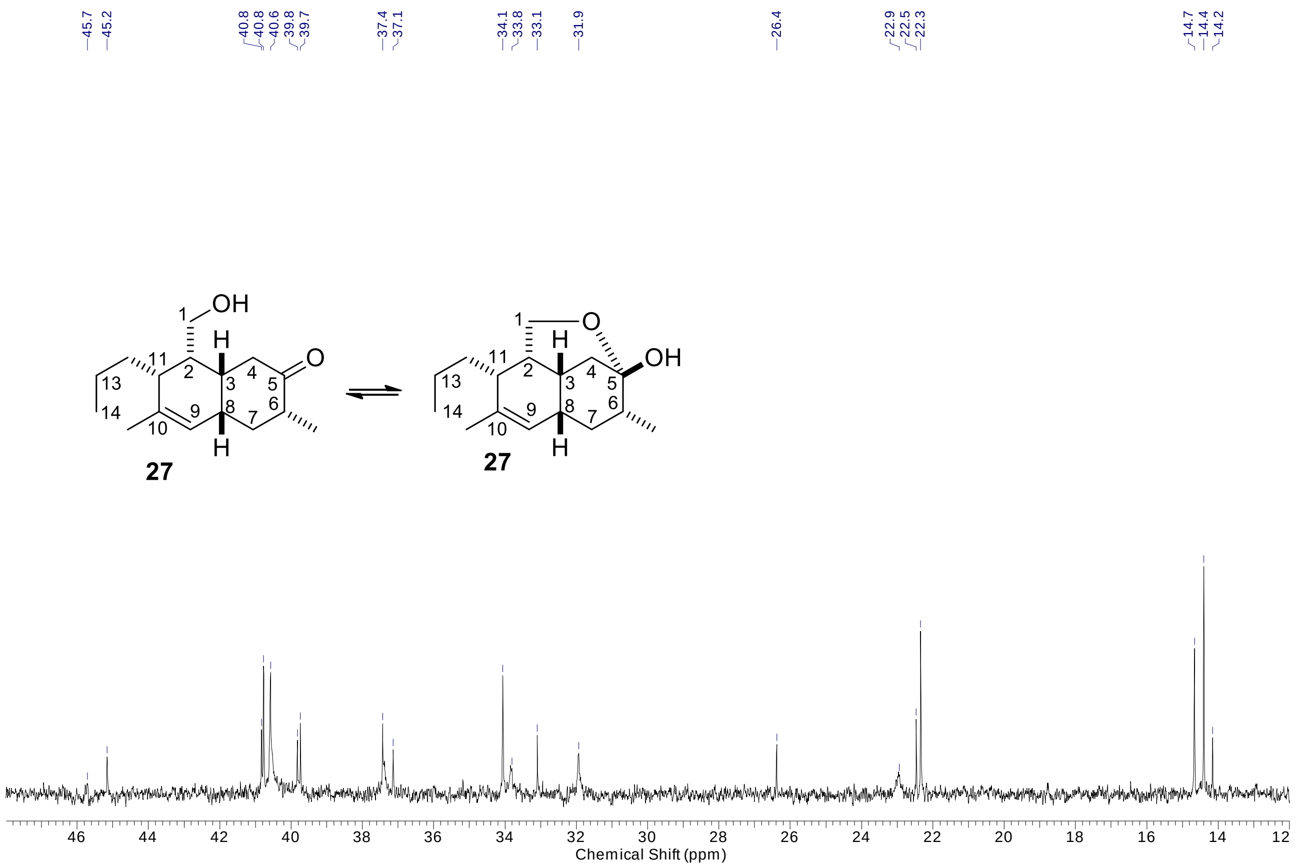

^{13}C NMR (100 MHz) spectrum of decalinone **27** in $CDCl_3$ (12 – 48 ppm)

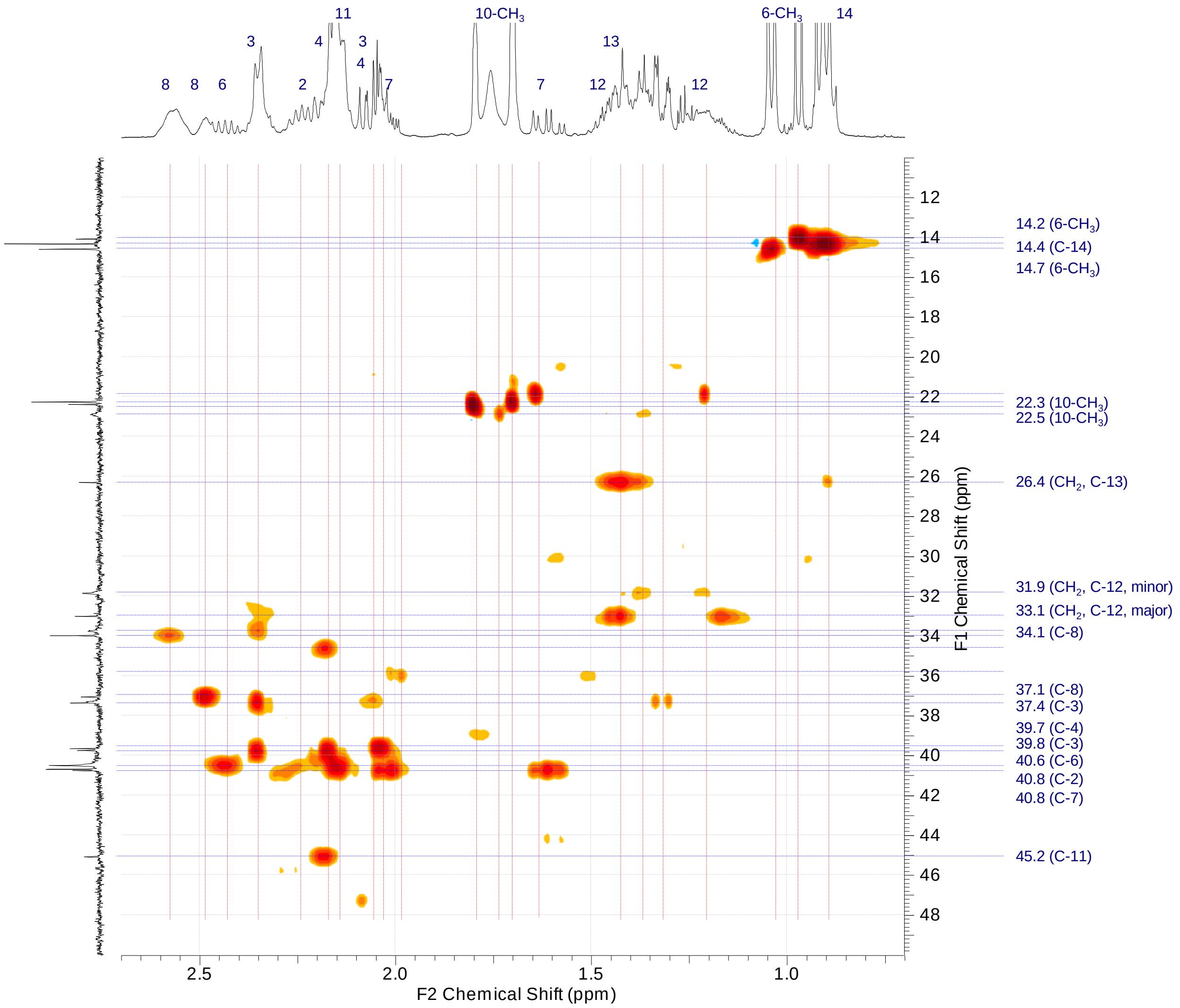

HSQC spectrum of decalinone **27** in $CDCl_3$ (0.7 – 2.7, 10 – 50 ppm)

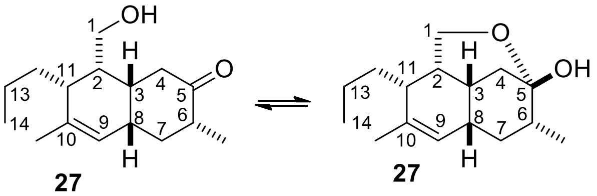

27 **27**

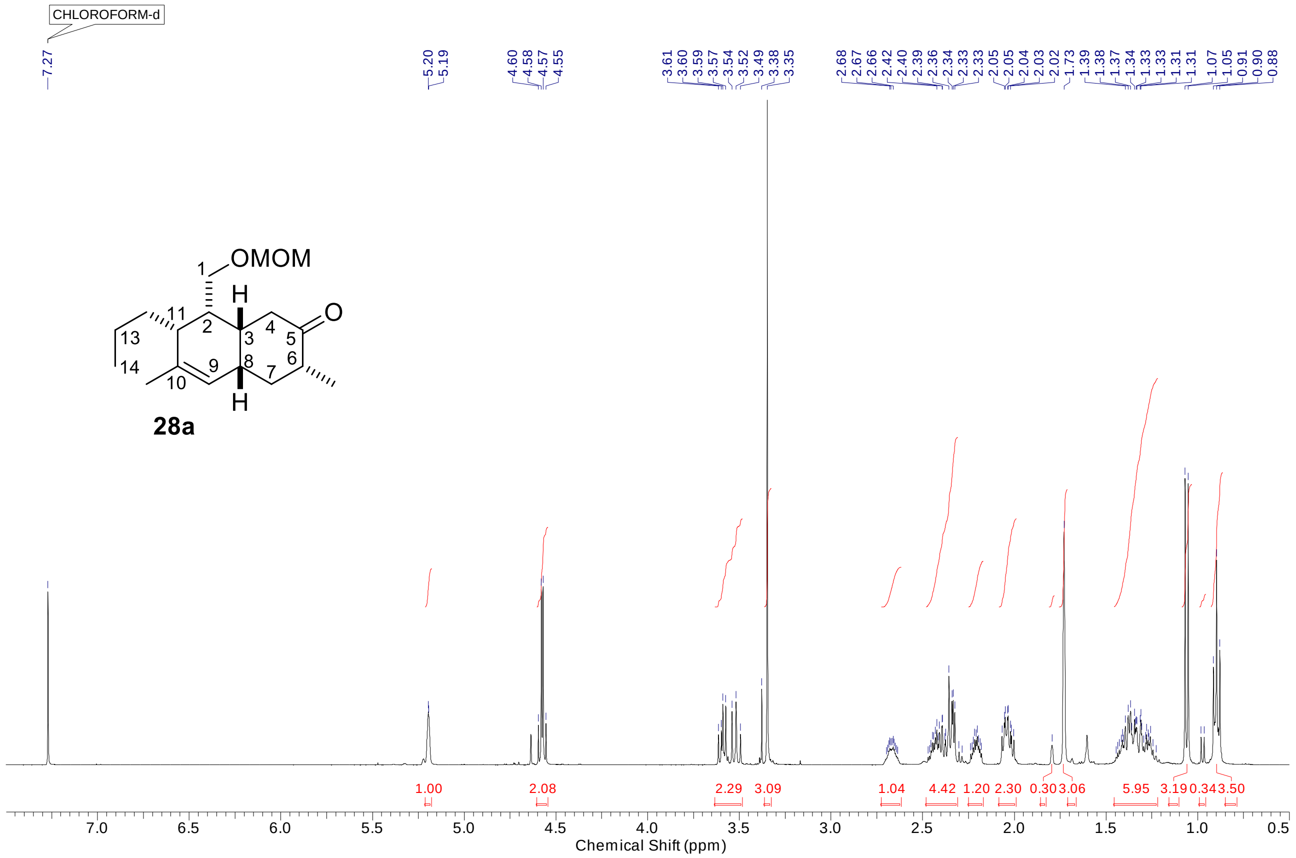

^{1}H NMR (400 MHz) spectrum of decalinone **28a** in $CDCl_3$ (0.5 – 7.5 ppm)

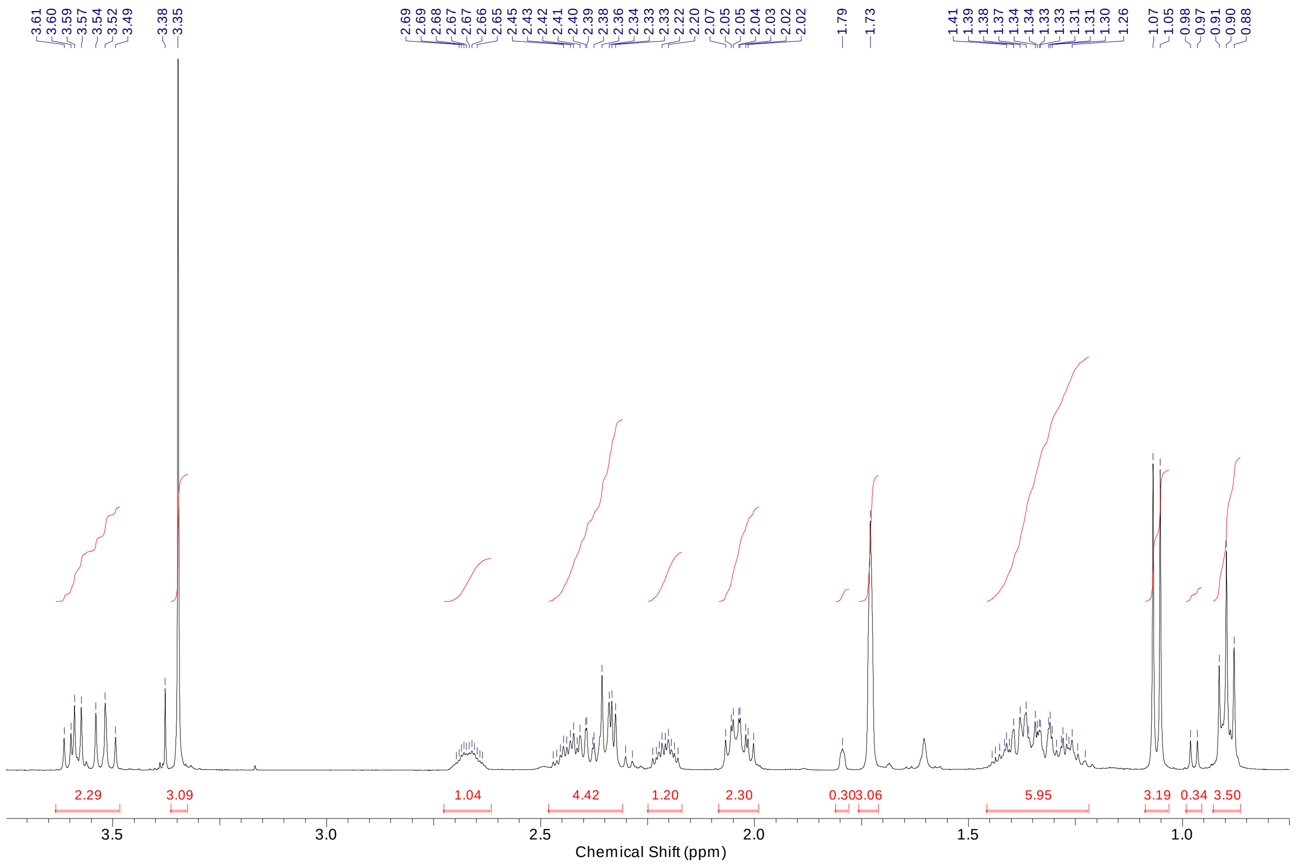

^{1}H NMR (400 MHz) spectrum of decalinone **28a** in $CDCl_3$ (0.75 – 3.75 ppm)

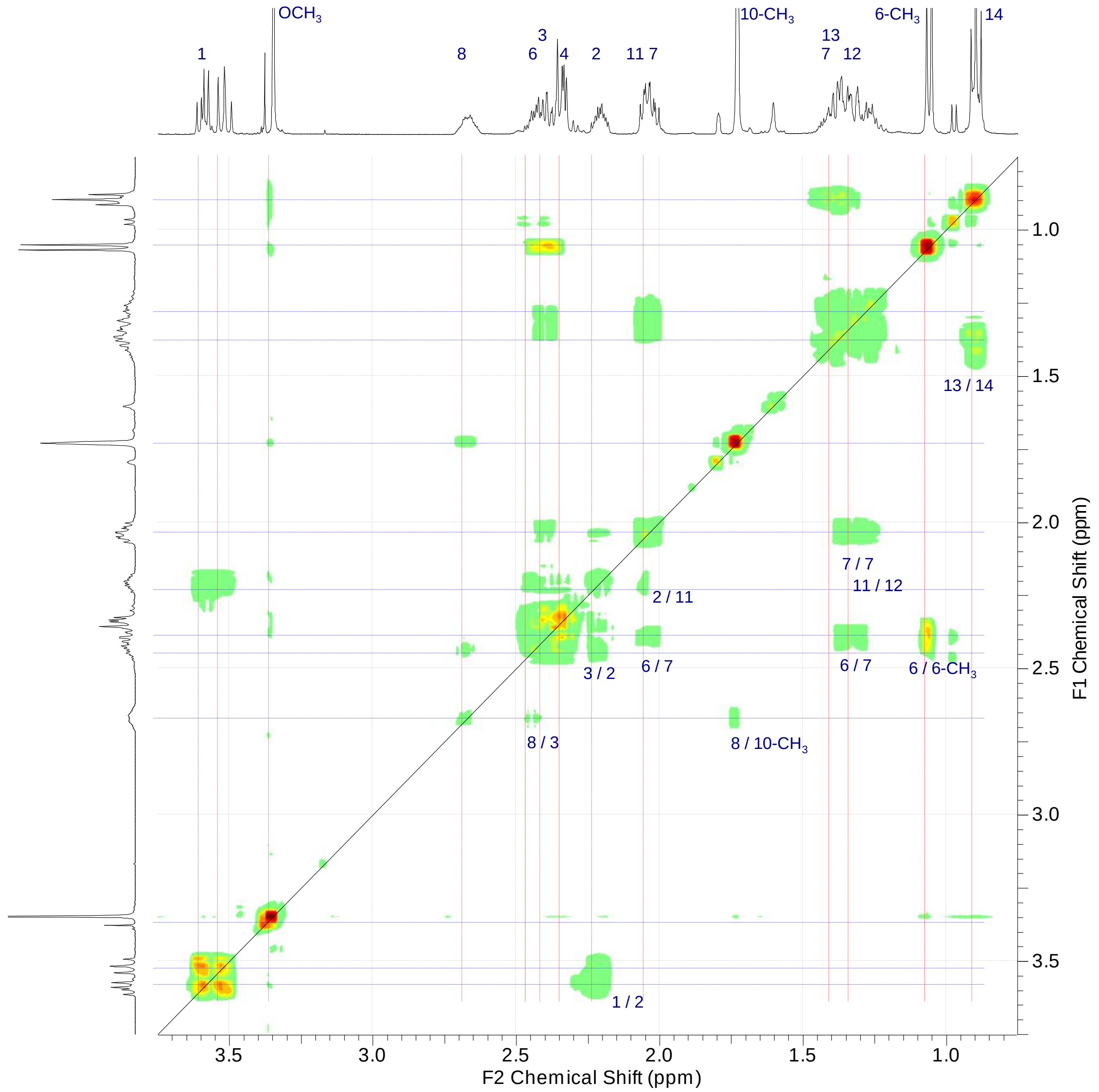

COSY spectrum of decalinone **28a** in $CDCl_3$ (0.75 – 3.75 ppm)

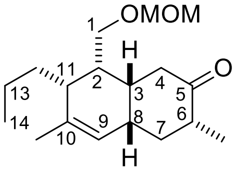

28a

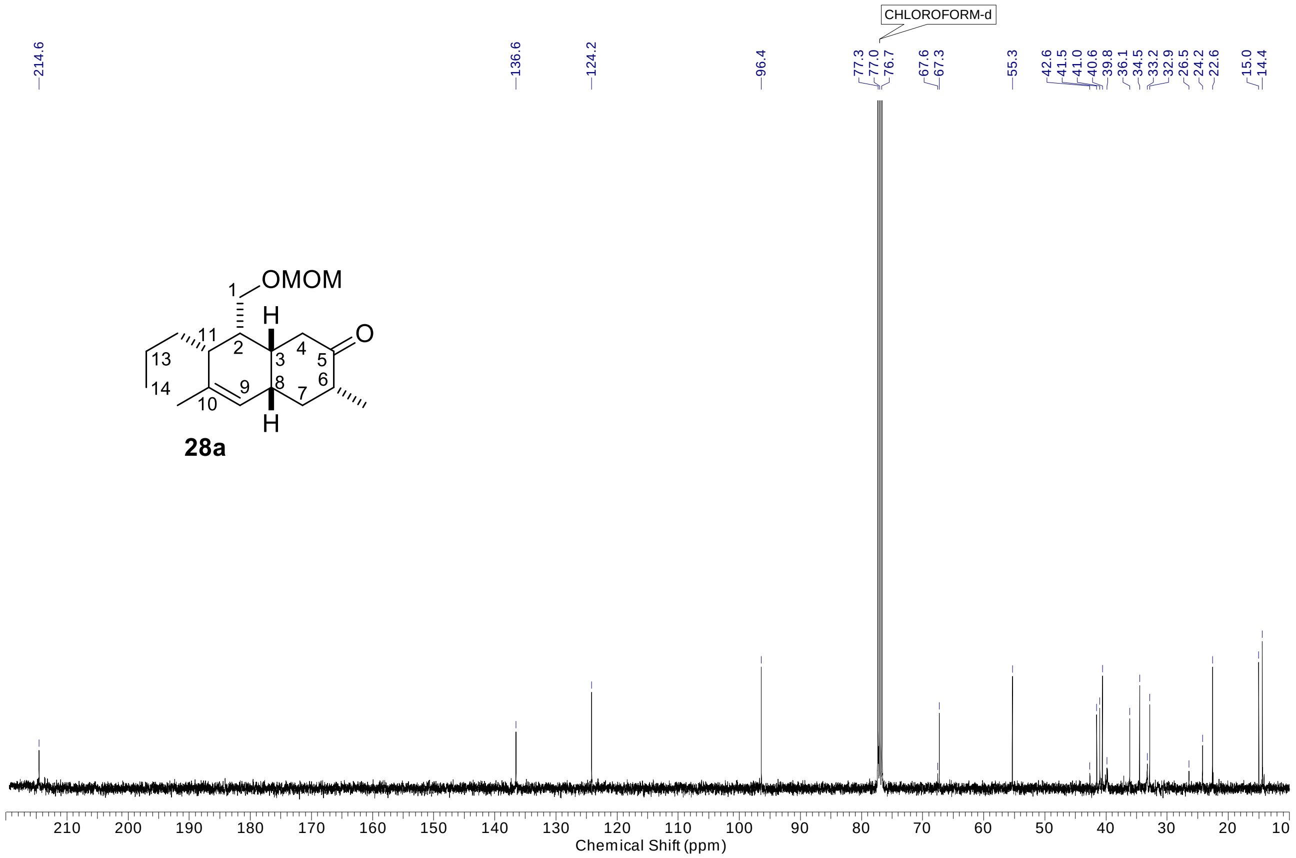

^{13}C NMR (100 MHz) spectrum of decalinone **28a** in $CDCl_3$ (10 – 220 ppm)

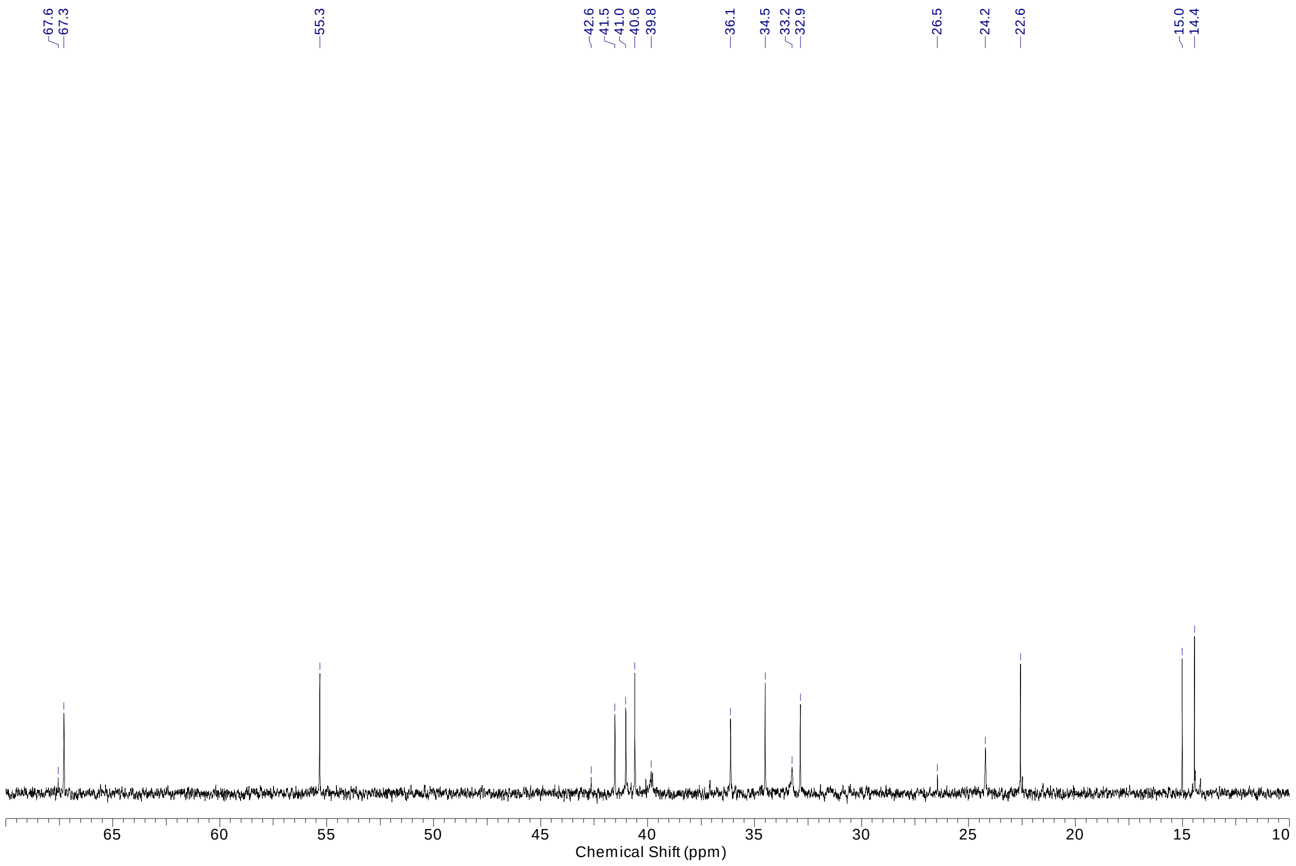

^{13}C NMR (100 MHz) spectrum of decalinone **28a** in $CDCl_3$ (10 – 70 ppm)

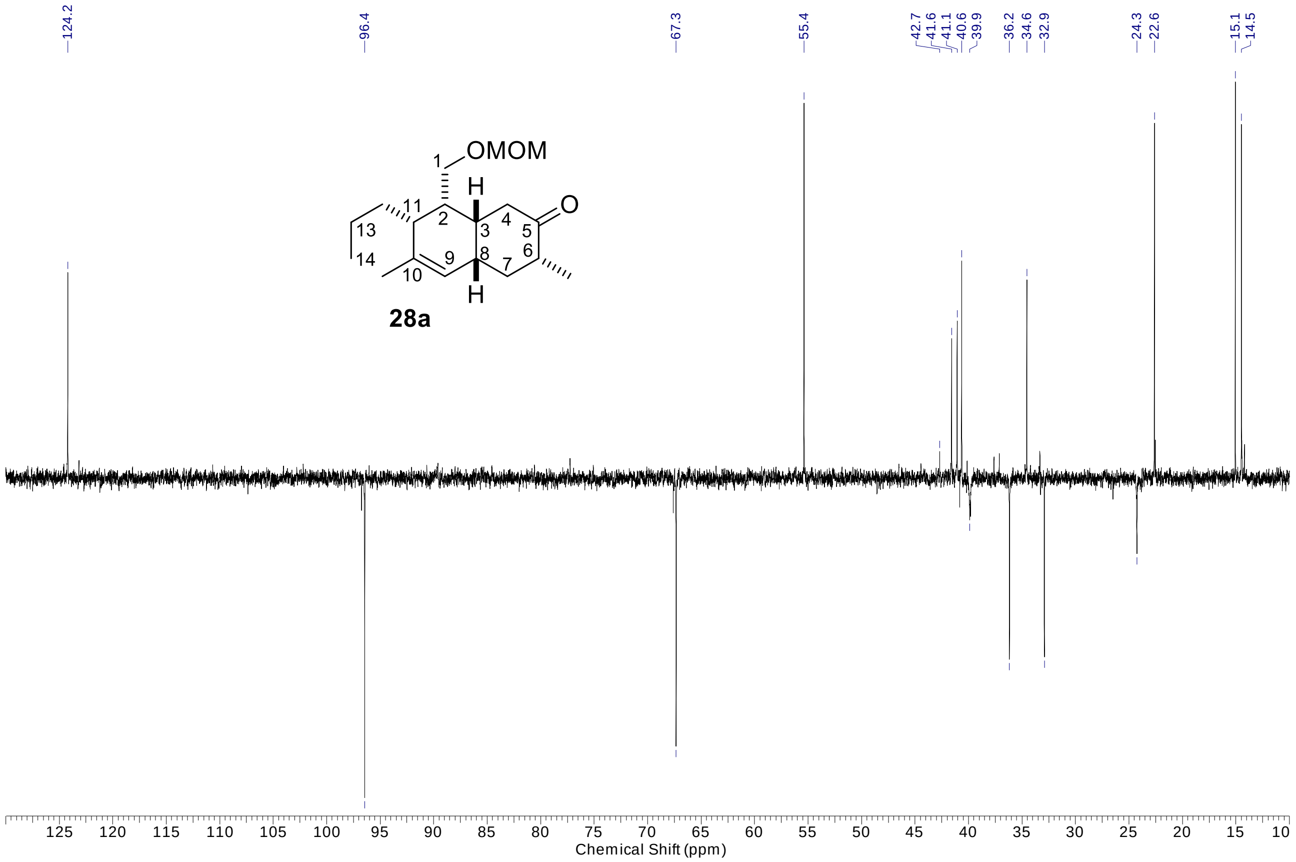

DEPT (100 MHz) spectrum of decalinone **28a** in $CDCl_3$ (10 – 130 ppm)

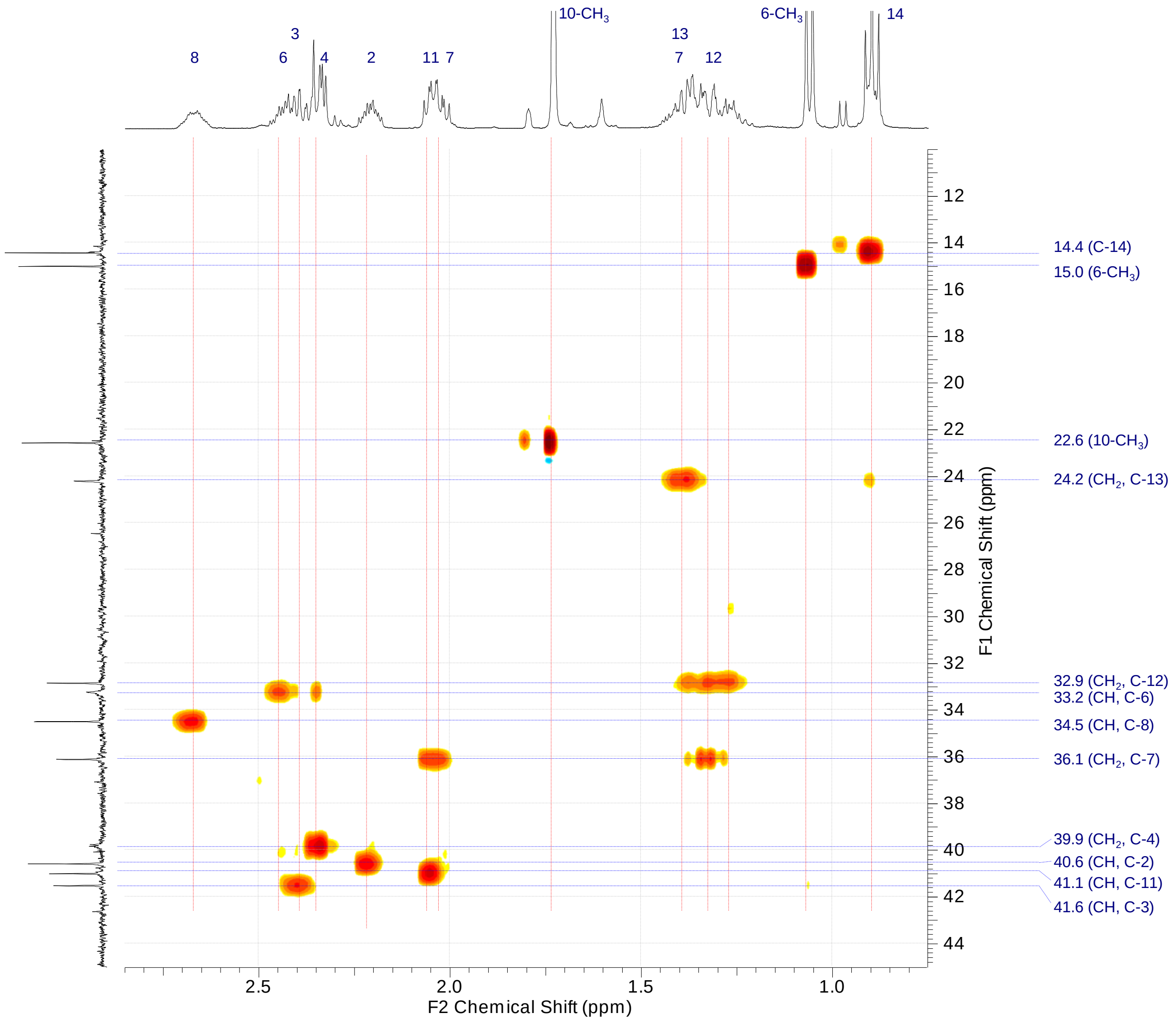

HSQC spectrum of decalinone **28a** in $CDCl_3$ (0.75 – 2.85, 10 – 45 ppm)

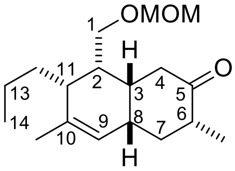

28a

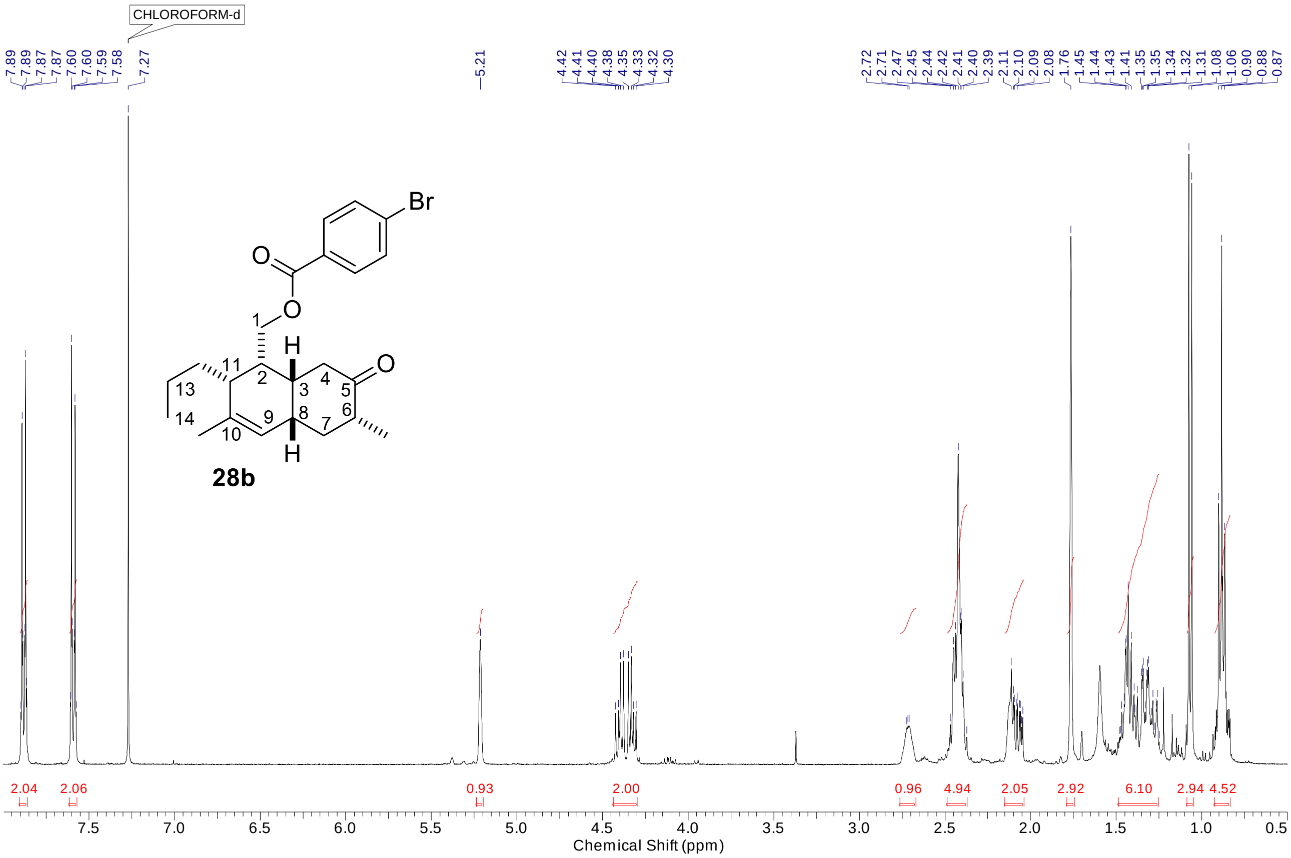

^{1}H NMR (400 MHz) spectrum of decalinone **28b** in $CDCl_3$ (0.5 – 8.0 ppm)

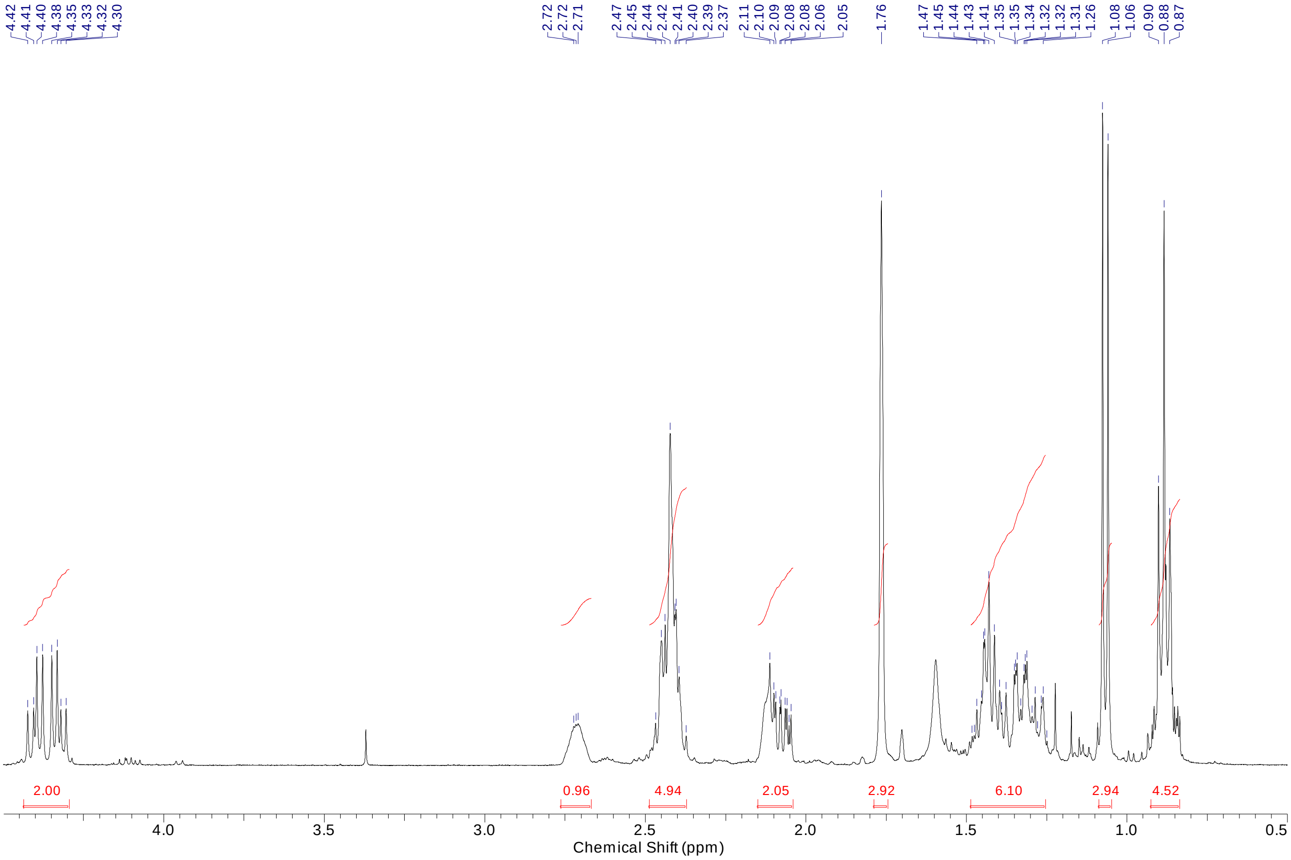

^{1}H NMR (400 MHz) spectrum of decalinone **28b** in $CDCl_3$ (0.5 – 4.5 ppm)

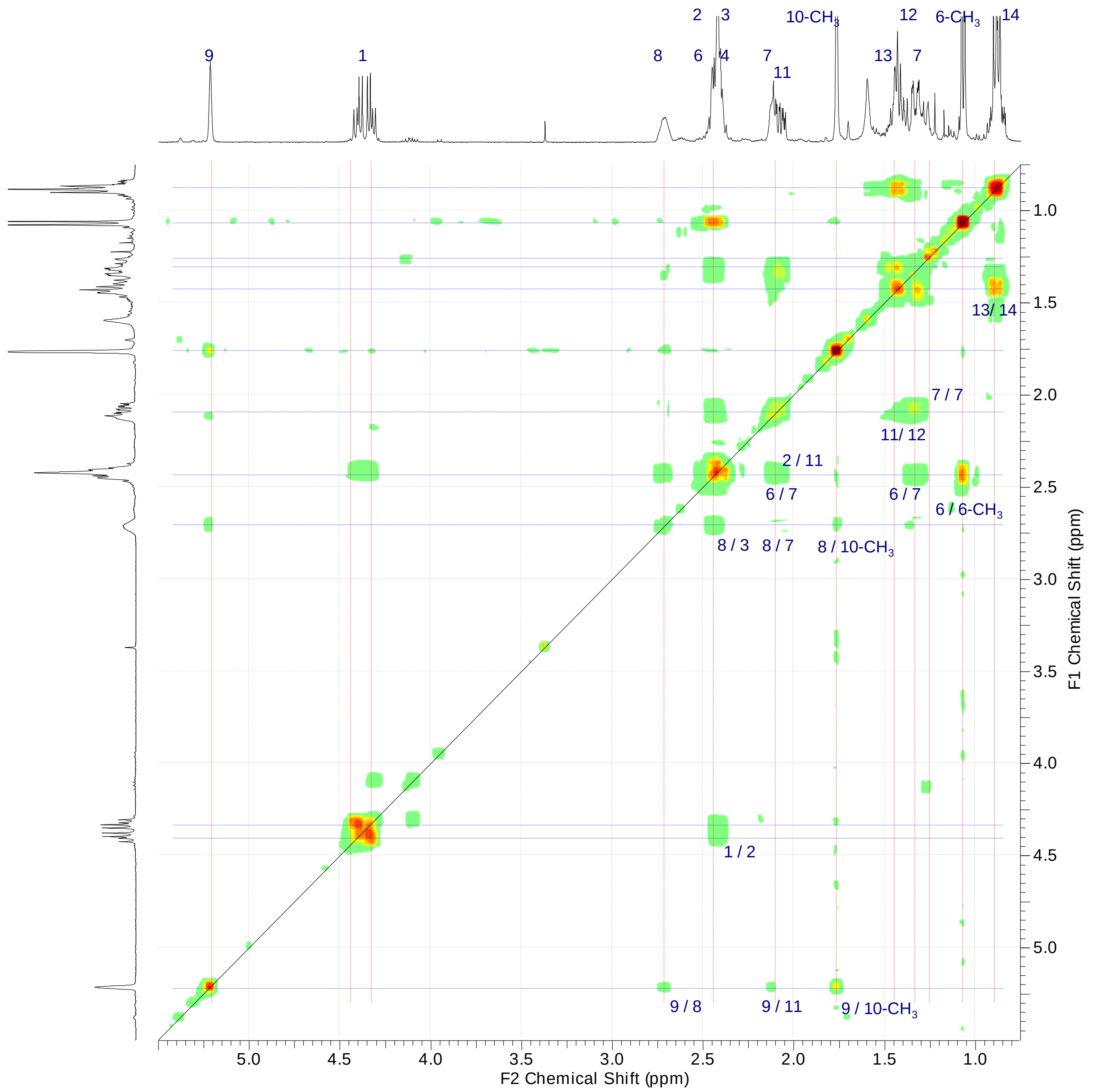

COSY spectrum of decalinone **28b** in $CDCl_3$ (0.75 – 5.5 ppm)

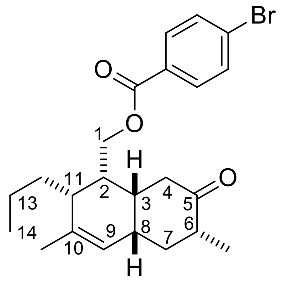

28b

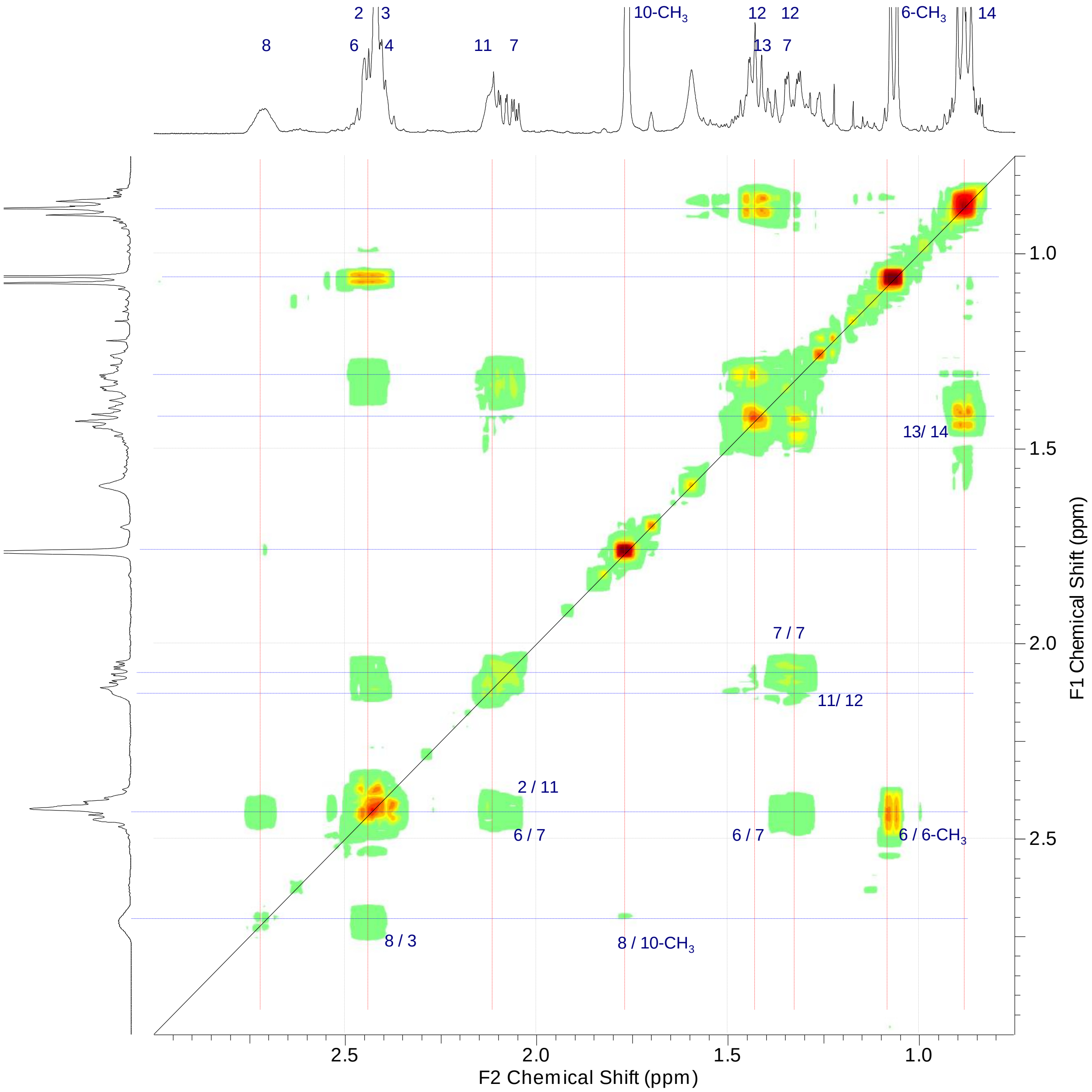

COSY spectrum of decalinone **28b** in $CDCl_3$ (0.75 – 3.0 ppm)

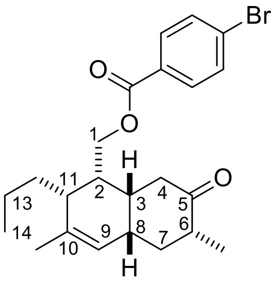

28b

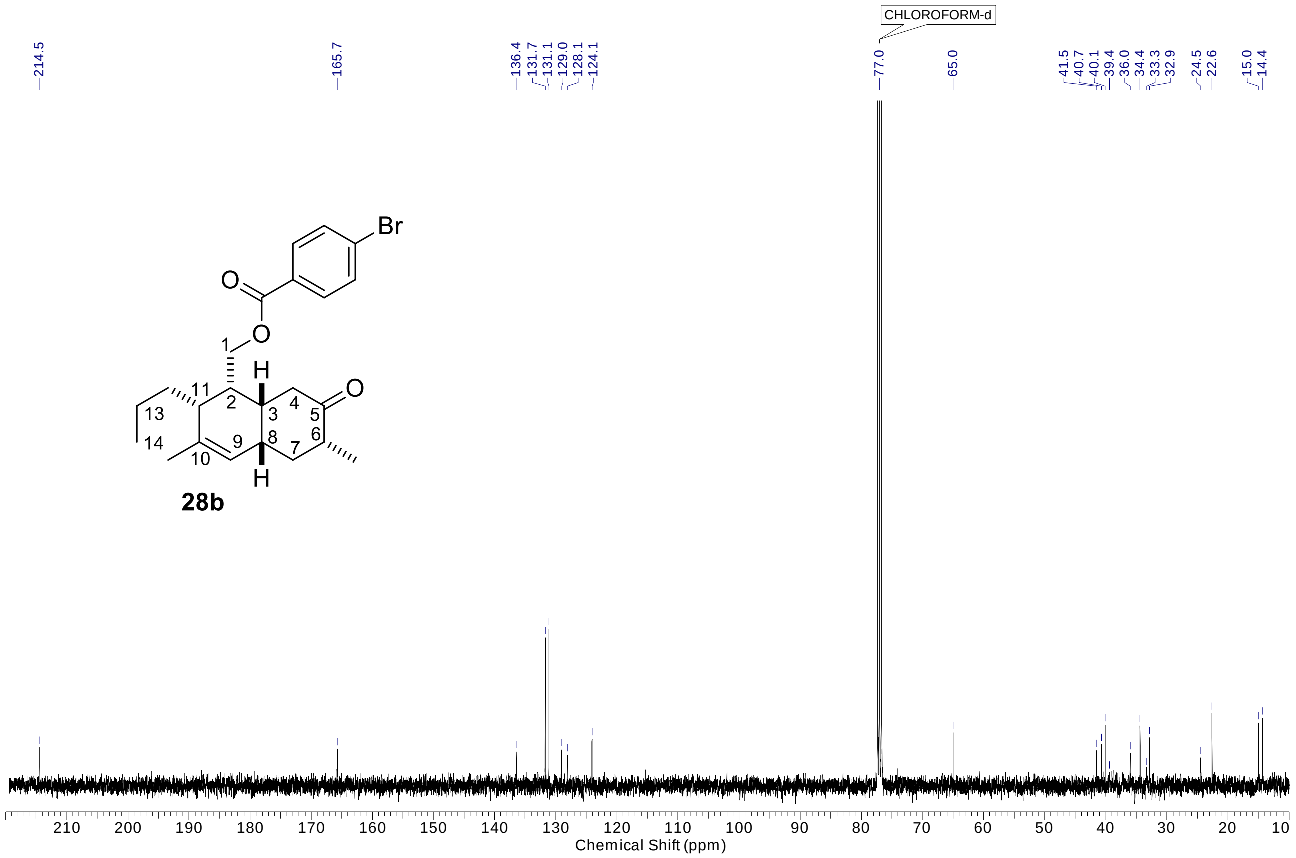

^{13}C NMR (100 MHz) spectrum of decalinone **28b** in $CDCl_3$ (10 – 220 ppm)

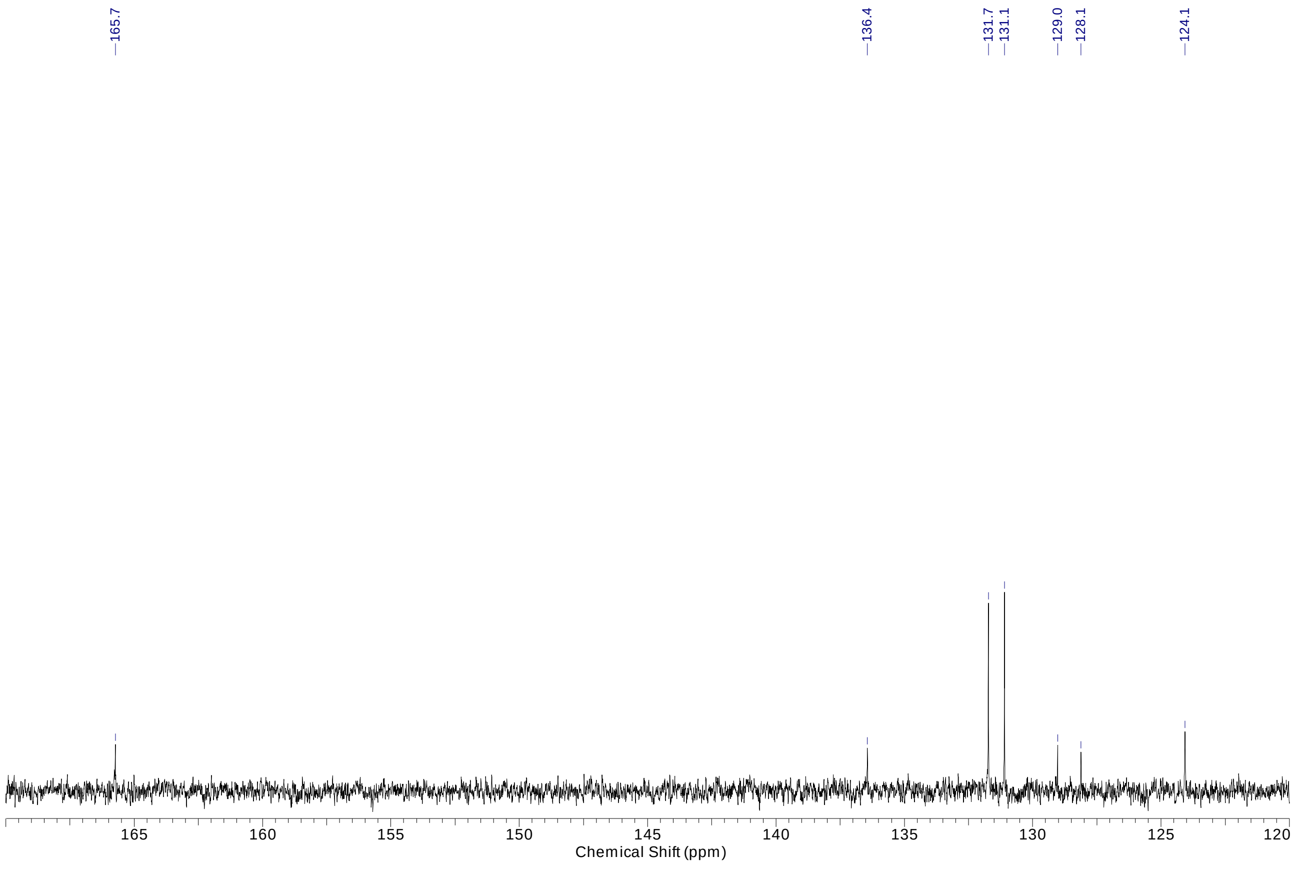

^{13}C NMR (100 MHz) spectrum of decalinone **28b** in $CDCl_3$ (120 – 170 ppm)

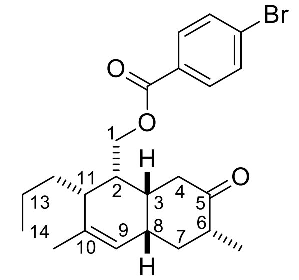

28b

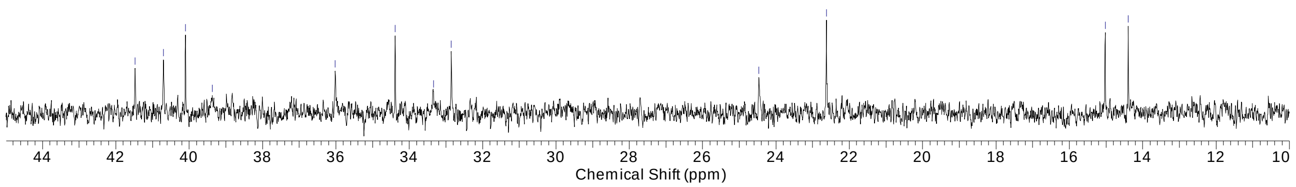

^{13}C NMR (100 MHz) spectrum of decalinone **28b** in $CDCl_3$ (10 – 45 ppm)

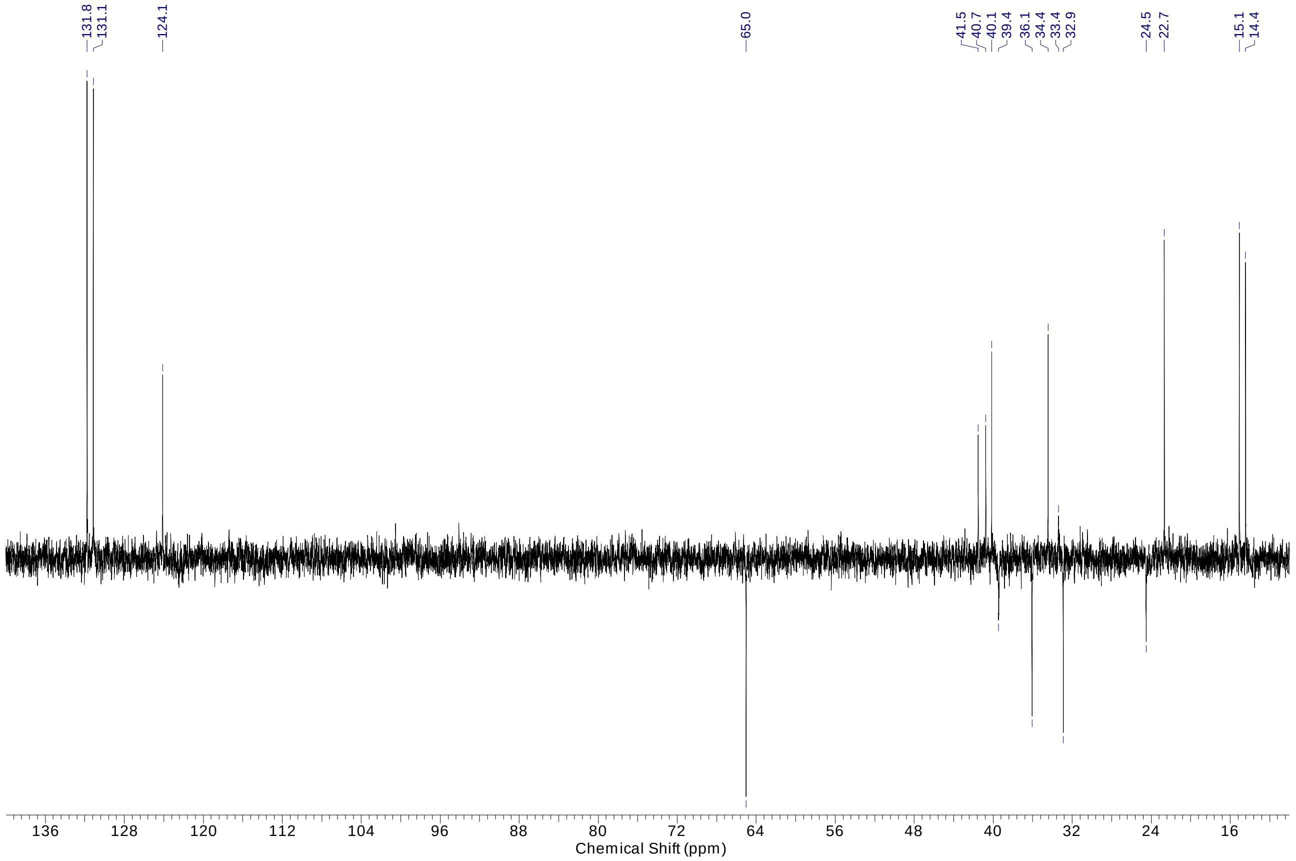

DEPT (100 MHz) spectrum of decalinone **28b** in $CDCl_3$ (10 – 140 ppm)

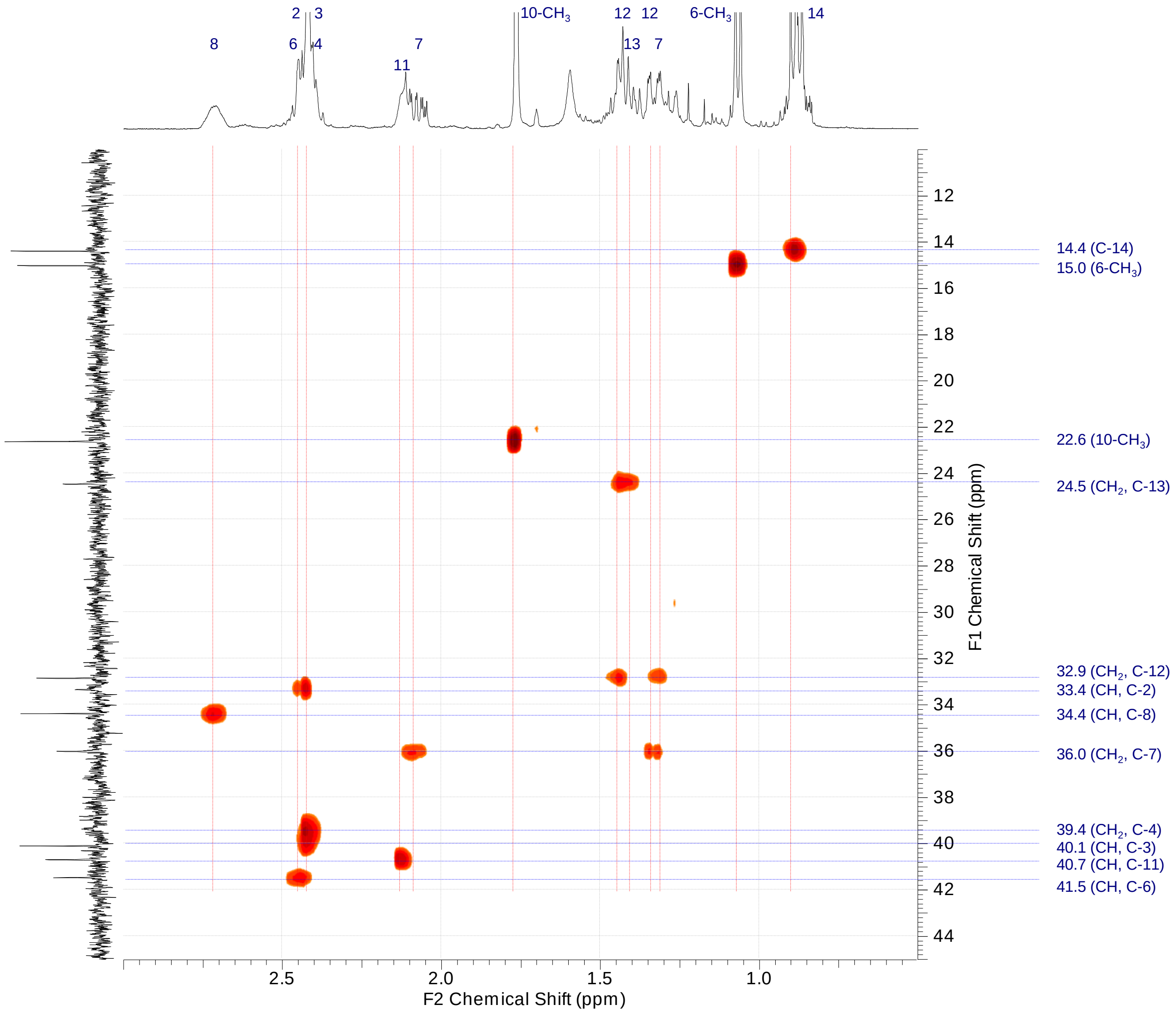

HSQC spectrum of decalinone **28b** in $CDCl_3$ (0.5 – 3.0, 10 – 45 ppm)

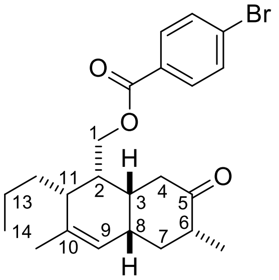

28b

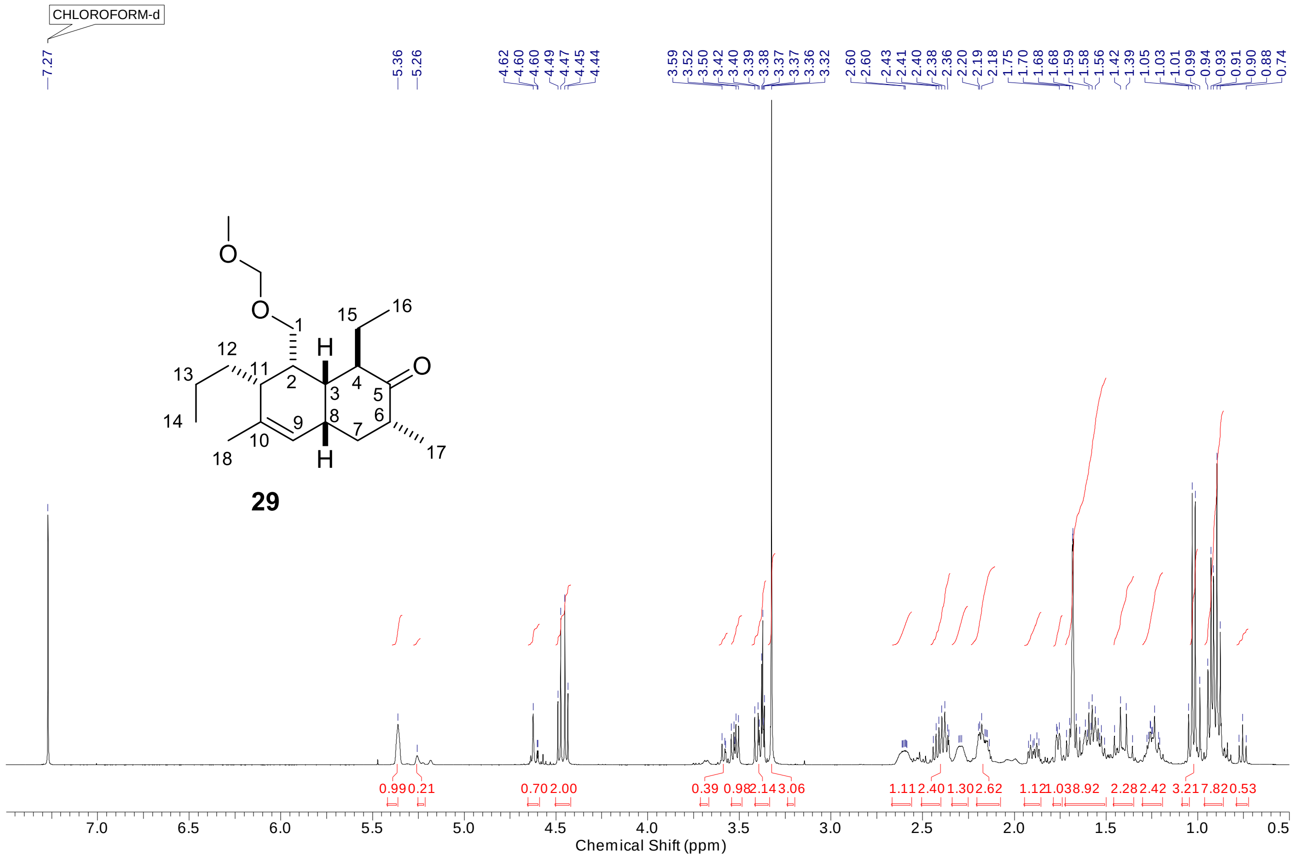

^{1}H NMR (600 MHz) spectrum of decalinone **29** in $CDCl_3$ (0.5 – 7.5 ppm)

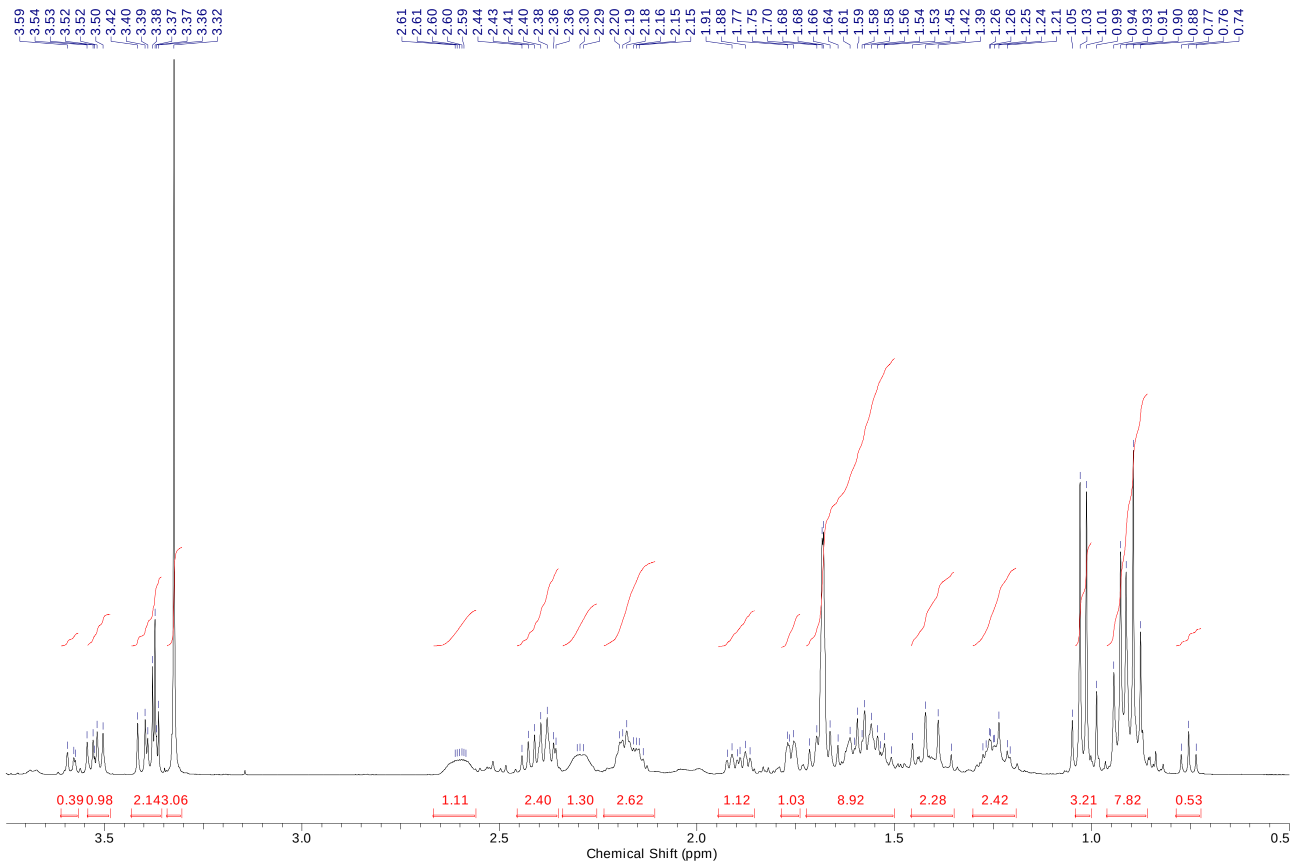

^{1}H NMR (600 MHz) spectrum of decalinone **29** in $CDCl_3$ (0.5 – 3.75 ppm)

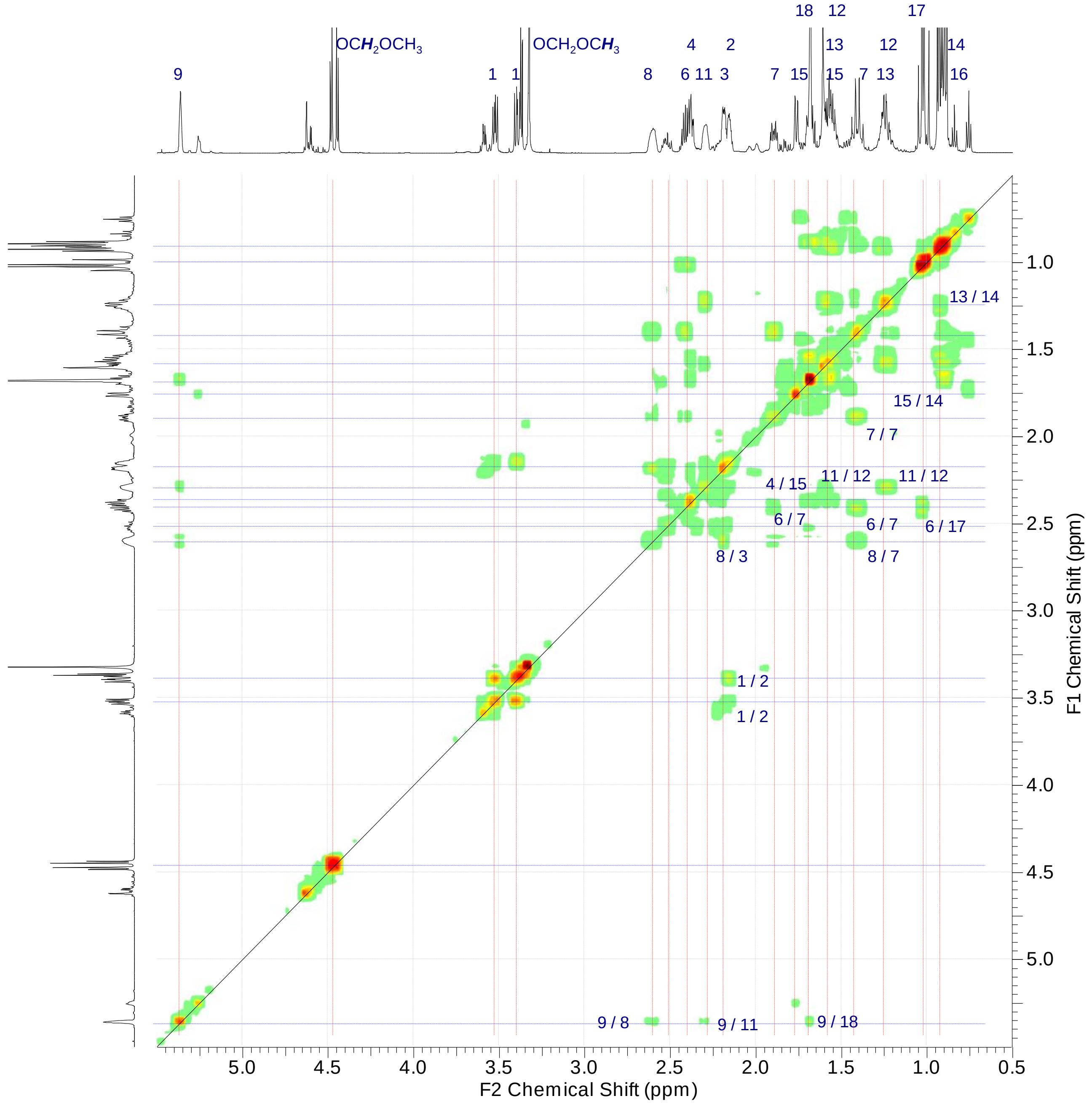

COSY spectrum of decalinone **29** in $CDCl_3$ (0.5 – 5.5 ppm)

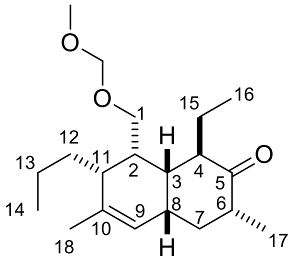

29

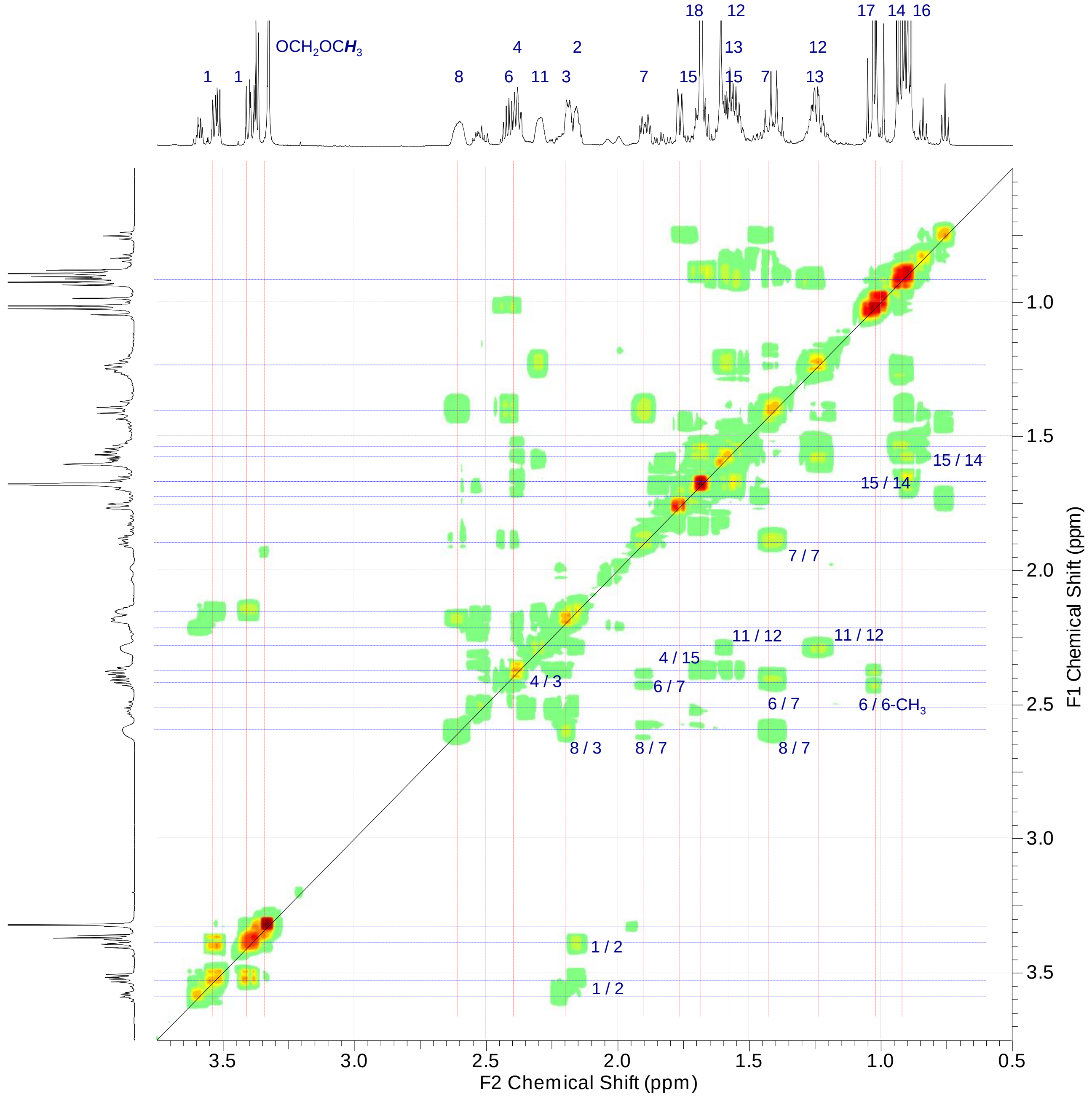

COSY spectrum of decalinone **29** in $CDCl_3$ (0.5 – 3.75 ppm)

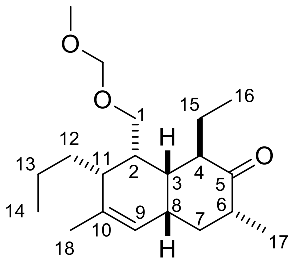

29

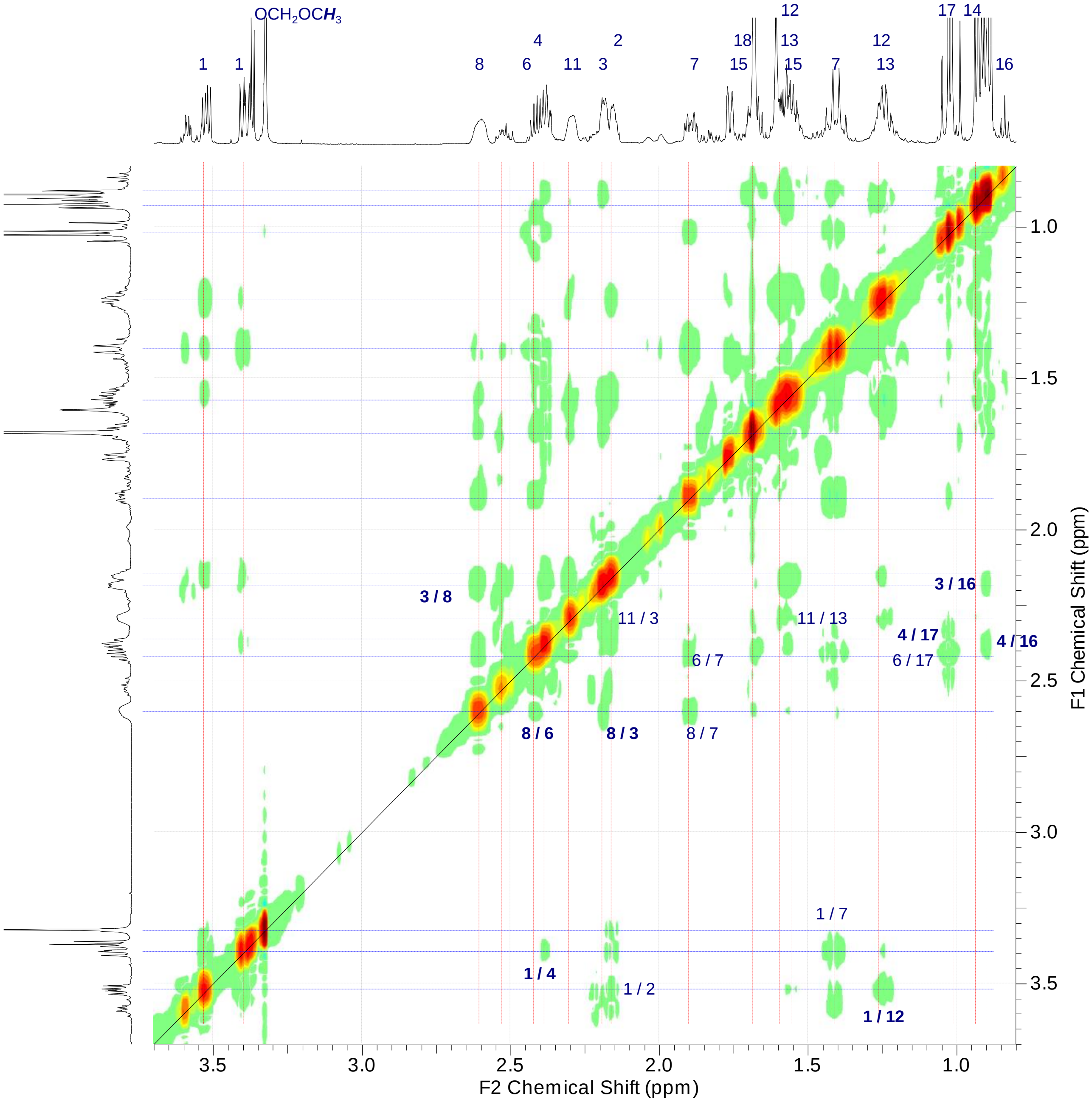

NOESY spectrum of decalinone **29** in $CDCl_3$ (0.8 – 3.7 ppm)

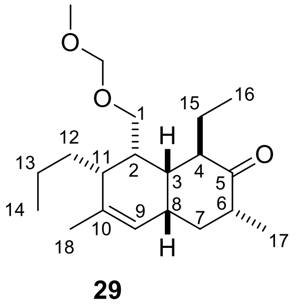

29

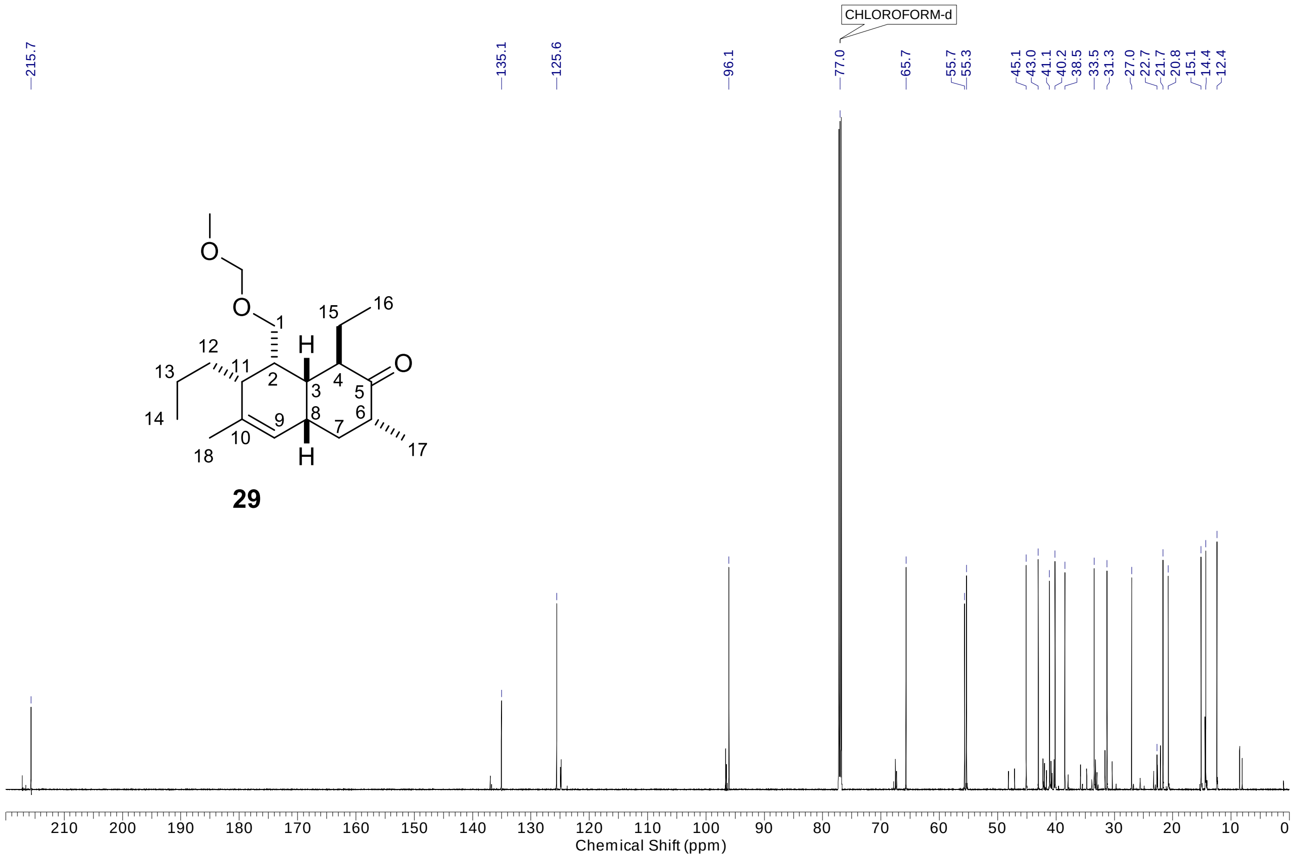

^{13}C NMR (150 MHz) spectrum of decalinone **29** in $CDCl_3$ (0 – 220 ppm)

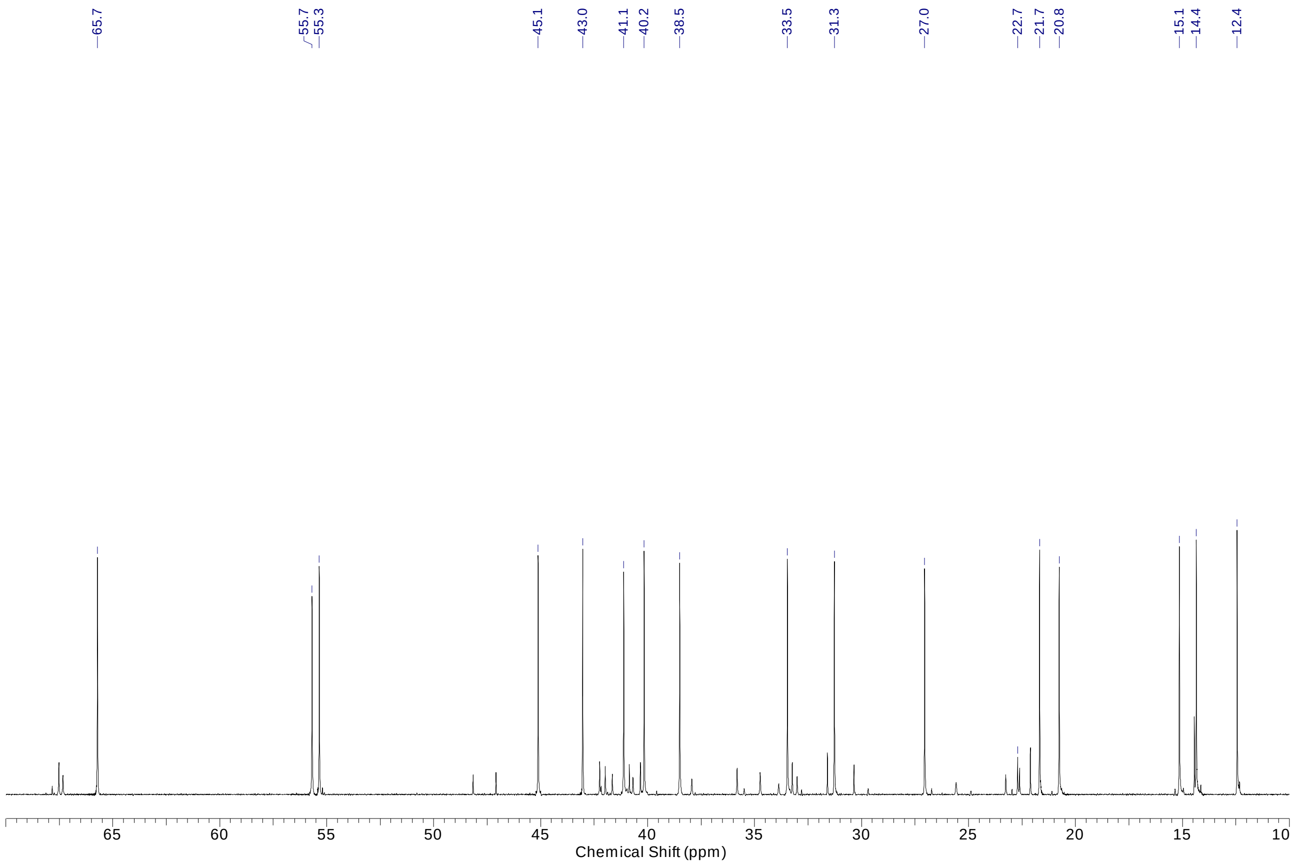

^{13}C NMR (100 MHz) spectrum of decalinone **29** in $CDCl_3$ (10 – 70 ppm)

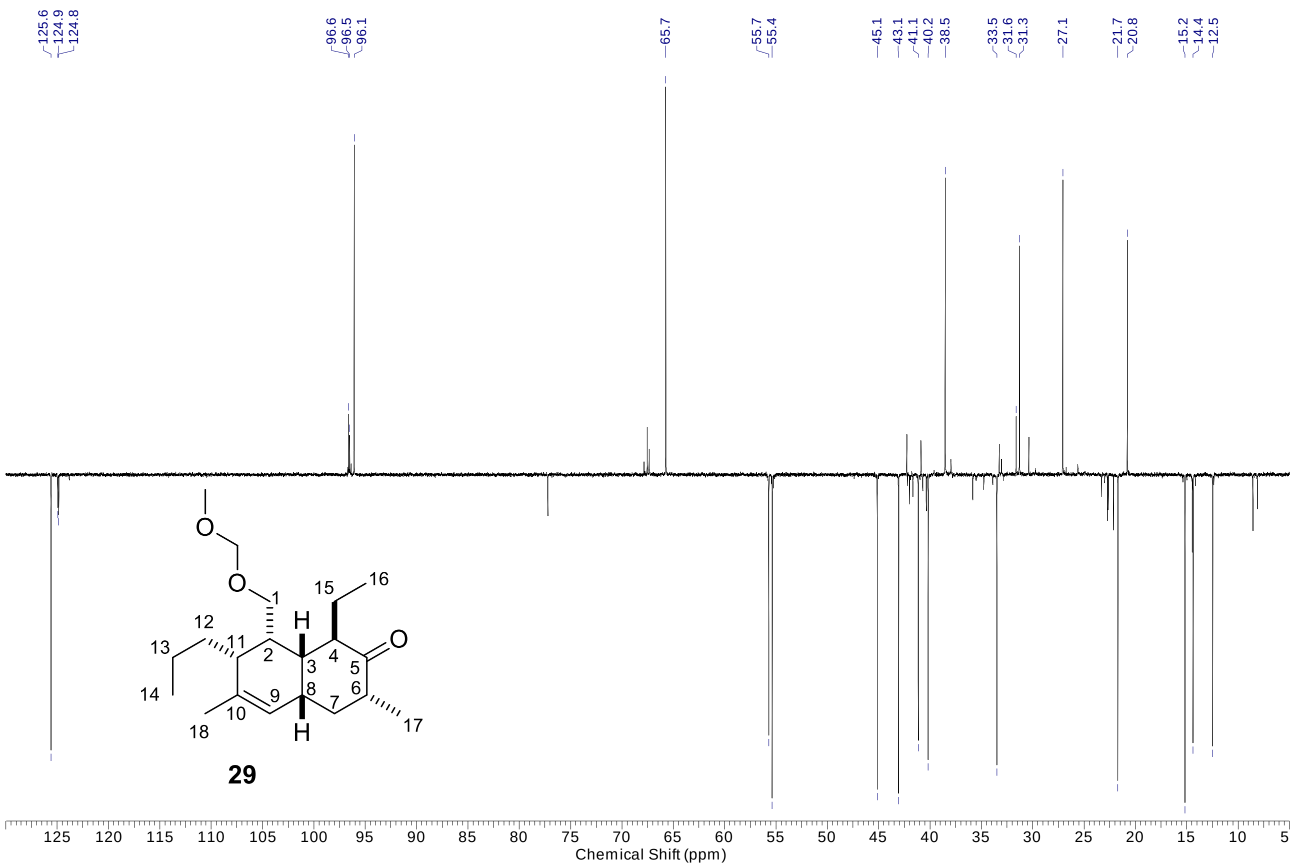

DEPT (100 MHz) spectrum of decalinone **29** in $CDCl_3$ (5 – 130 ppm)

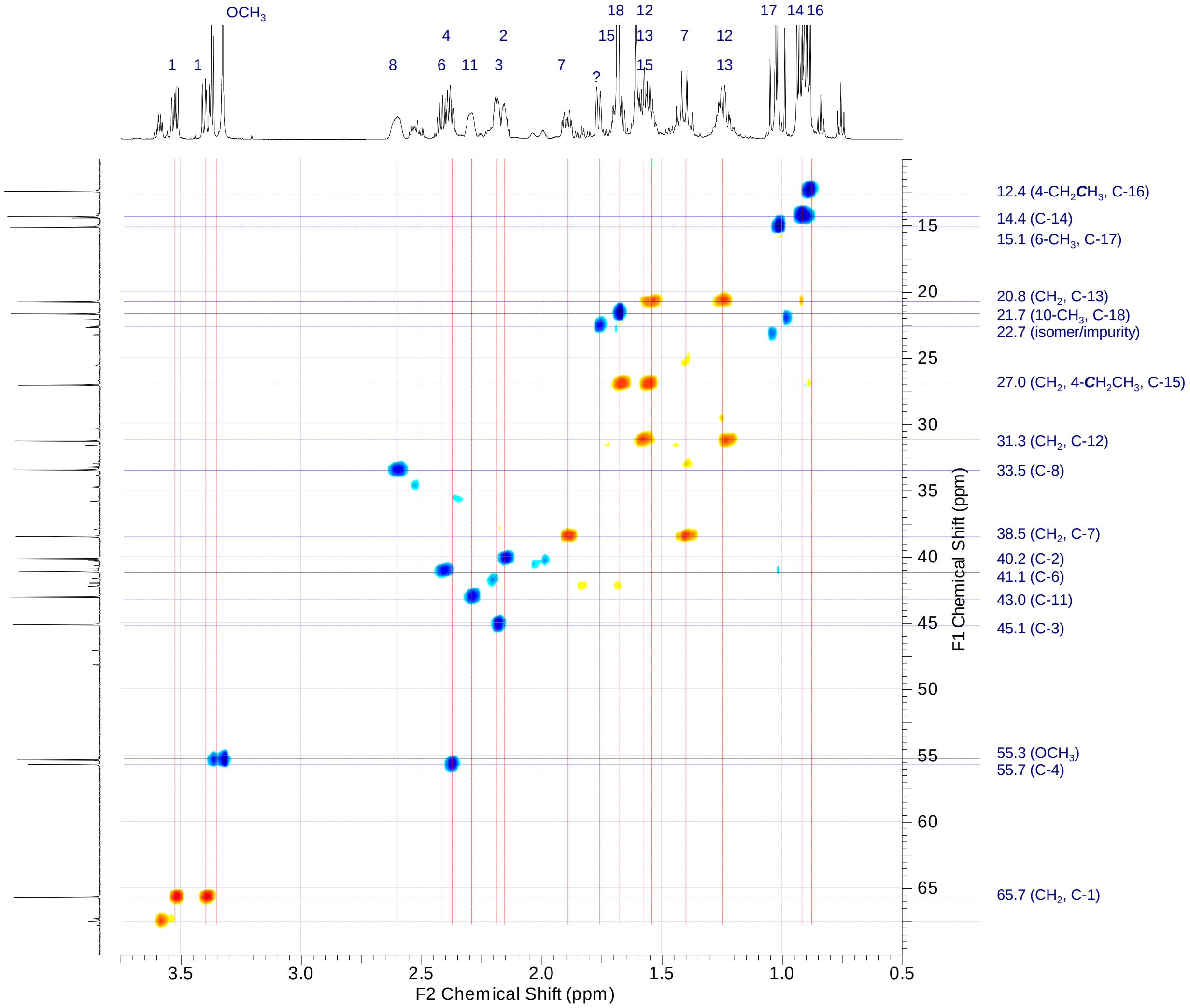

HSQC spectrum of decalinone **29** in $CDCl_3$ (0.5 – 3.75, 10 – 70 ppm)

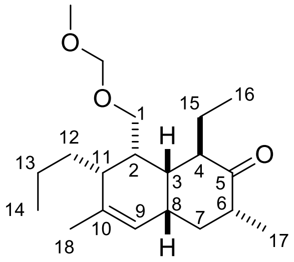

29
